# Rose without prickle: genomic insights linked to moisture adaptation

**DOI:** 10.1093/nsr/nwab092

**Published:** 2021-05-22

**Authors:** Mi-Cai Zhong, Xiao-Dong Jiang, Guo-Qian Yang, Wei-Hua Cui, Zhi-Quan Suo, Wei-Jia Wang, Yi-Bo Sun, Dan Wang, Xin-Chao Cheng, Xu-Ming Li, Xue Dong, Kai-Xue Tang, De-Zhu Li, Jin-Yong Hu

**Affiliations:** CAS Key Laboratory for Plant Diversity and Biogeography of East Asia, Kunming Institute of Botany, Chinese Academy of Sciences, Kunming 650201, China; University of Chinese Academy of Sciences, Beijing 100049, China; CAS Key Laboratory for Plant Diversity and Biogeography of East Asia, Kunming Institute of Botany, Chinese Academy of Sciences, Kunming 650201, China; University of Chinese Academy of Sciences, Beijing 100049, China; Germplasm Bank of Wild Species, Kunming Institute of Botany, Chinese Academy of Sciences, Kunming 650201, China; CAS Key Laboratory for Plant Diversity and Biogeography of East Asia, Kunming Institute of Botany, Chinese Academy of Sciences, Kunming 650201, China; University of Chinese Academy of Sciences, Beijing 100049, China; CAS Key Laboratory for Plant Diversity and Biogeography of East Asia, Kunming Institute of Botany, Chinese Academy of Sciences, Kunming 650201, China; University of Chinese Academy of Sciences, Beijing 100049, China; Flower Research Institute, Yunnan Agricultural Academy of Sciences, Kunming 650231, China; CAS Key Laboratory for Plant Diversity and Biogeography of East Asia, Kunming Institute of Botany, Chinese Academy of Sciences, Kunming 650201, China; University of Chinese Academy of Sciences, Beijing 100049, China; CAS Key Laboratory for Plant Diversity and Biogeography of East Asia, Kunming Institute of Botany, Chinese Academy of Sciences, Kunming 650201, China; University of Chinese Academy of Sciences, Beijing 100049, China; Biomarker Technologies Corporation, Beijing 101300, China; Biomarker Technologies Corporation, Beijing 101300, China; CAS Key Laboratory for Plant Diversity and Biogeography of East Asia, Kunming Institute of Botany, Chinese Academy of Sciences, Kunming 650201, China; Germplasm Bank of Wild Species, Kunming Institute of Botany, Chinese Academy of Sciences, Kunming 650201, China; Flower Research Institute, Yunnan Agricultural Academy of Sciences, Kunming 650231, China; CAS Key Laboratory for Plant Diversity and Biogeography of East Asia, Kunming Institute of Botany, Chinese Academy of Sciences, Kunming 650201, China; Germplasm Bank of Wild Species, Kunming Institute of Botany, Chinese Academy of Sciences, Kunming 650201, China; CAS Key Laboratory for Plant Diversity and Biogeography of East Asia, Kunming Institute of Botany, Chinese Academy of Sciences, Kunming 650201, China

**Keywords:** *Rosa wichuraiana* ‘Basye's Thornless’, prickle-free, water storage, gene-regulatory-network (GRN), QTL

## Abstract

Prickles act against herbivores, pathogens or mechanical injury, while also preventing water loss. However, whether prickles have new function and the molecular genetics of prickle patterning remain poorly explored. Here, we generated a high-quality reference genome assembly for ‘Basye's Thornless’ (BT), a prickle-free cultivar of *Rosa wichuraiana*, to identify genetic elements related to stem prickle development. The BT genome harbors a high level of sequence diversity in itself and with cultivar ‘Old Blush’ (*R. chinensis*), a founder genotype in rose domestication. Inheritance of stem prickle density was determined and two QTL were identified. Differentially expressed genes in QTL were involved in water-related functions, suggesting that prickle density may hitchhike with adaptations to moist environments. While the prickle-related gene-regulatory-network (GRN) was highly conserved, the expression variation of key candidate genes was associated with prickle density. Our study provides fundamental resources and insights for genome evolution in the Rosaceae. Ongoing efforts on identification of the molecular bases for key rose traits may lead to improvements for horticultural markets.

## INTRODUCTION

Understanding the molecular genetic mechanisms driving morphological variation remains a great challenge. When we say, ‘there are no roses without thorns,’ we employ a metaphor meaning we are willing to endure something unpleasant because it is associated with something good. In fact, rose prickles are acuminate protuberances formed by the deformation of plant trichomes that are often mistaken for true spines or thorns. Prickles are usually interpreted as epidermal adaptations protecting plants from herbivores, pathogens or mechanical injury. In the children's book, *Wild Animals I have Known* (1898), author Ernest S. Thompson depicted the rose bush as a protector of cottontail rabbits while the same prickles harmed people and other animals. However, prickles also increase the thickness of epidermis which reduces heat and water dissipation [1,2]. Many plants in the families Rosaceae, Araliaceae, Fabaceae and Rutaceae bear prickles on their leaves, stems and fruits. Trichomes serve as developmental models to study the control of cell proliferation and growth in *Arabidopsis and Gossypium*, thus allowing the patterning gene-regulatory-network (GRN) to be investigated in detail [1,3–6]. However, the molecular and genetic mechanisms underlying stem prickle development have not been studied systematically.

With more than 36 000 cultivars, roses have long been revered for their beauty and fragrance, despite the presence of abrasive prickles on their stems. Roses offer far more morphological novelty, including continuous flowering (CF), prickles, multi-petal corollas and complicated floral scents absent in such current model systems as *Arabidopsis*, rice and poplar. Therefore, the rose serves as an additional woody plant model for studying important traits with commercial values [7,8]. However, rose genetics are unusually complex because of past histories of natural inter- and intra-specific hybridization, polyploidy and unbalanced meiosis, which is further exaggerated under human selection [9–11]. Determination of a highly heterozygous genome for roses remains challenging, as seen by the very fragmented assembly trials on three species [12,13]. Currently, two versions of chromosome-level genome sequences for doubled-haploid materials of one cultivated founder genotype (*Rosa chinensis* ‘Old Blush’, haploOB) have been described [12,14]. Enormous sequence variations can exist between rose cultivars as in maize, and they may contribute to key agricultural traits [15]. Therefore, a complete picture of genome assembly for diploid roses is necessary for comparative genomics to identify the molecular genetic bases of key morphological traits pursued after centuries of breeding.

**Figure 1.**
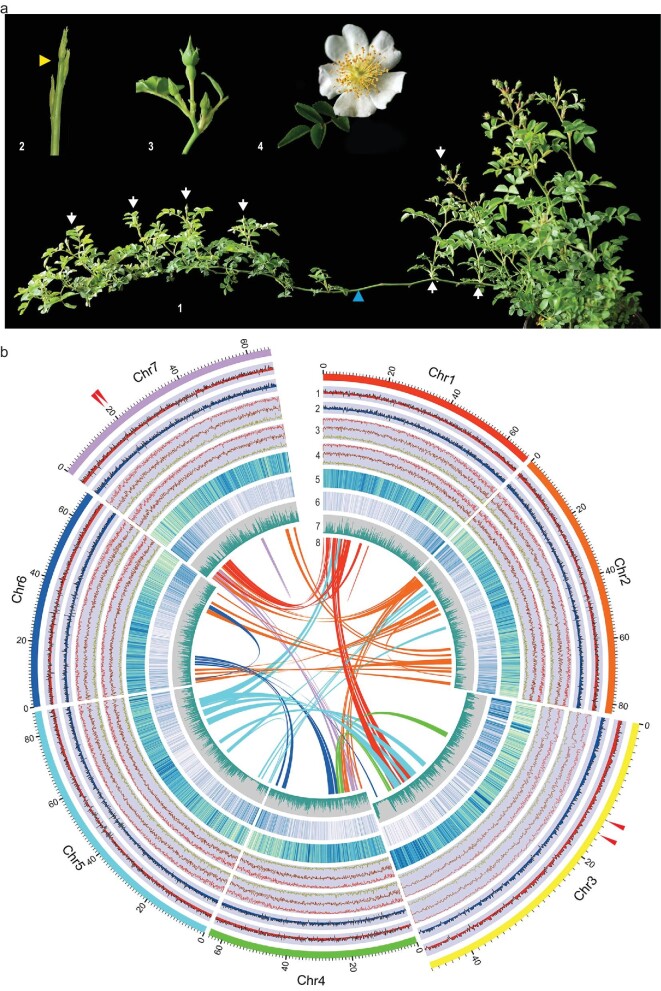
Anatomical and genome features of *R. wichuraiana* ‘Basye's Thornless’ (BT). (a) Growth habit of flowering BT plant in April 2020. 1, Branches bearing flowering buds erect/sub-erect (white arrows) while vegetative stems remain prostrate (blue arrowhead). 2, Early flowering stage (orange arrowhead). 3, Flower bud prior to anthesis. 4, Fully-open flower. (b) Landscape of genome features for BT genome. 1, Gene expression profiles for leaf materials grown in March 2017. 2, Gene expression profiles for leaf materials grown in November 2016. 3 and 4, DNA methylation patterns for samples in 1 (3) and 2 (4). Lines in red, dark red and brown mark the CG, CHG and CHH methylation, respectively. 5, Contents of transposable elements (TEs) in each chromosome. 6, Gene density in each chromosome. 7, SNP density along chromosomes in every 100 kb bins. 8, Internal syntenic blocks within BT chromosomes. Arrow heads mark the two QTL regions.

The glandular-trichome-based prickles on rose stems [16] are inconvenient in floriculture and commercial nurseries. Although most rose genotypes harbor prickles, the ‘prickle-free’ cultivar known as ‘Basye's Thornless’ (BT) is a variant of *R. wichuraiana* Crep., a diploid founder species (2n = 14) in rose domestication (Fig. 1a) [8,17–19]. It has been used to create hundreds of commercial hybrids, with ‘Pink Roamer’ being the first [18]. BT grows in a relatively moist environment. The BT cultivar has at least six traits differing from the OB cultivar, a founder in hybridization used even more frequently. Compared with OB, the BT flowers only once a year, bears prickle-free stems and shows prostrate growth. While each flower bears only a single whorl of white petals, it continues to show a higher resistance to black spot [18]. This explains why BT is often crossed with OB. Unfortunately, molecular information on BT is limited [14,20–26]. Here, we determined the chromosome-level genome sequences of BT. We systematically analyzed the prickle QTL and GRN. We hypothesize that the characteristic of being ‘prickle-free’ may hitchhike on genomic segments selected for adaptation to relatively warm and humid climates.

## RESULTS AND DISCUSSION

### *De novo* chromosome-level assembly of the BT genome

Flow cytometry estimated the genome size of BT to be 525.9 (±5.91) Mb. The initial genome survey analysis with ∼267x Illumina reads predicted a genome size of 525.5 Mb with ∼1.03% heterozygosity. We generated ∼93x PacBio Sequel and ∼90x Oxford Nanopore Technology (ONT) reads and used a *CANU* based hybrid strategy to construct contigs (Fig. S1; Tables S1 and S2). We obtained 1554 contigs of 530 Mb (Table S3 and S4). We built a chromosome-level assembly using 34.64 million unique Di-Tags read pairs produced via Hi-C analysis (Table S5), which generated 2711 contigs, among which 857 (52.97% in 1618 clustered) contigs were ordered and oriented into seven pseudo-molecules spanning 481.77 Mb (92.41% of 521.32 Mb total sequences clustered; Table S6). There were 46.18 Mb sequences not ordered/oriented. After manual correction, we obtained the final assembly of the BT genome (530.07 Mb). Chr5 was the longest (87.44 Mb) while Chr3 the shortest (46.04 Mb) (Fig. 1b, Fig. S2, and Tables S6 and S7).

### Reference-quality and high completeness of the BT genome

**Figure 2.**
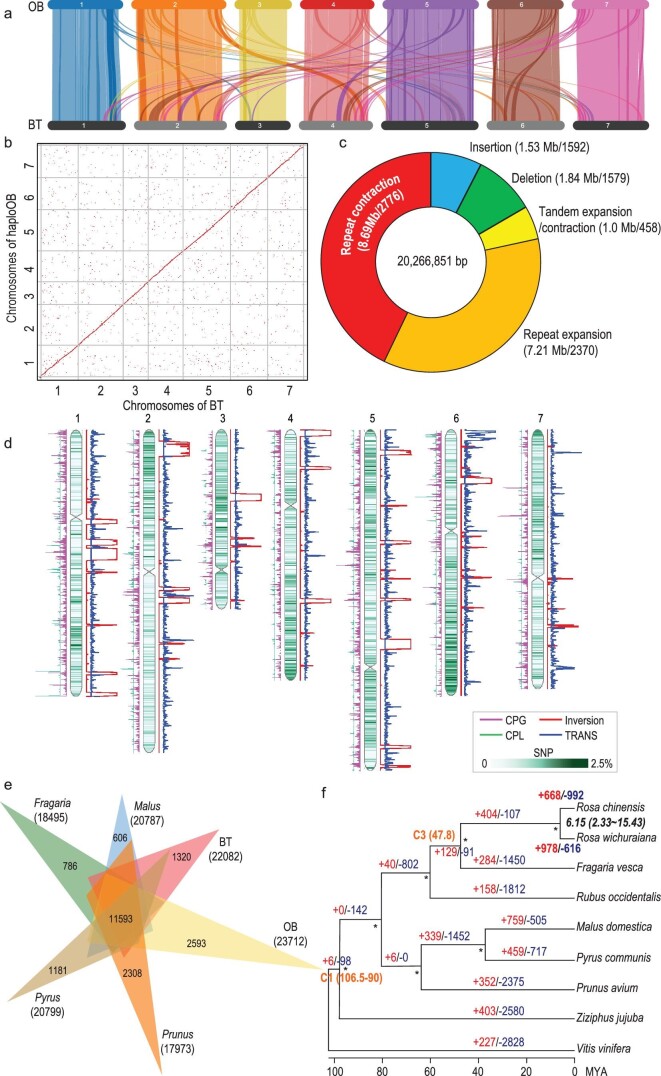
Comparative genomic analyses between BT, OB and other members of the Rosaceae. (a) Gene collinearity (minimum 10 syntenic gene pairs) between the BT and Raymond's haploOB genomes. High level of collinearity between chromosome pairs indicated by light color lines/blocks. Dark color lines mark genome segments with relatively lower collinearity. (b) Macrosynteny patterns between BT and haploOB (Raymond *et al.* 2018) genomes along each chromosome. Dots closest to the diagonal line represent collinearity between the two genomes with <10 Kb fragments filtered out. (c) Doughnut chart showing large-size sequence variation between BT and haploOB. Numbers in brackets indicate the total length of each type of variation (before/) and the number of events (after/). (d) Genome alignment between BT and haploOB identifying significant structural variants including genome inversions (red lines), translocation (TRANS; blue lines), copy gain (CPG; cyan lines; in OB with BT as reference) and copy loss (CPL; green lines). Data plotted as per type of variation in 1 Mb of genomic region with step size of 100 kb by length density. SNP density plotted on the chromosomes with 2.5 SNPs per 100 bp length at the highest level. (e) Comparison of the numbers of gene families identified via OrthoMCL. Venn diagram showing shared homologous gene families among genomes of BT, haploOB, *Fragraria*, *Malus*, *Pyrus* and *Prunus*. Numbers in brackets indicate total gene families. Only numbers of genome-specific and shared among six genomes shown. Numbers in brackets show total gene families for each species. (f) Phylogenetic relationships of BT with other taxa in the Rosaceae based on 1 230 shared single-copy orthologous genes with *Ziziphus jujuba* and *Vitis vinifera* (outgroups). Estimated divergence time between BT and OB following the fossil calibrations of nodes C1 (106.5–90 MYA) and C3 (47.8 MYA) (in orange, according to Zhang *et al.*, 2018). Numbers on branches indicate the counts of gene family expansion (+, in red) and contraction (−, in blue) along each lineage. ^*^ gives the bootstrap supports above 95% in 500 times simulation.

We used several methods to evaluate the quality and completeness of the BT genome. First, this genome was subjected to Benchmarking Universal Single-Copy Orthologs (BUSCO, embryophyta_odb10) analysis. Overall, 93.9% (2184; complete) and 3.0% (69; fragmented) of the 2326 expected plant gene models were identified (Table S8). Two independent genetic maps based on different populations were used to assess the quality of the BT genome. The collinearity with the K5 genetic map [27] was very high, with most of the association efficiency above 95% (Table S9; Fig. S3). Chr4 was an exception in the OBxBT genetic map, in which the distribution of molecular markers was obviously skewed by a high proportion of distorted markers [25]. The remaining six chromosomes showed collinearity at ∼90% correlation rate (Table S9; Fig. S4). We next aligned our BT assembly to the recently reported haploOB genome assemblies [12,14], and identified a very high collinearity (Fig. 2a and b; Fig. S3). This revealed a high synteny level and gene order between the genotypes. We re-mapped and obtained high mapping rates of the Illumina (98.16%) and PacBio (96.39%) reads (Table S10). A per-base estimate of the quality-value (QV) reached 34.61. Finally, the genome-wide LTR Assembly Index value of BT was 20.04, a level of golden reference (Fig. S5). Collectively, these indicated that the BT genome was of high quality and completeness, comparable with the two haploOB genomes (Table S11).

### A high proportion of *Copia* type LTRs in the BT genome

Repeat sequences represented about 65.19% (345.58 Mb) of the BT genome, in which LTR retrotransposons were about 45.09% (or 69.16% of all repeats; Fig. 1b and Table S12). In contrast to haploOB [12,14], but similar to the *Musa balbisiana* assembly [28], the BT genome had many more *Copia* elements (28.29% of the genome, or 62.74% of all LTRs) than *Gypsy* (16.39%). The distribution of *Copia* type LTRs was antagonistic to that of the *Gypsy* type on almost all BT chromosomes (Fig. S6). The ∼0.58 *Gypsy*-to-*Copia* ratio was one of the lowest in the sequenced species. This suggests that *Copia* amplification contributes much more than other types of transposonable elements (TEs) to BT genome evolution (Table S13 and Fig. S7). In general, the dense distribution of TEs in the pericentromeric regions correlated inversely with gene density on the BT chromosomes (Fig. 1b).

### The BT genome has 32 674 protein-coding genes

Gene annotation of the BT genome generated a total of 32 674 protein-coding genes, with an average gene length of ∼3.28 kb, an average exon length of ∼251 bp and a mean intron length of 390 bp (Fig. 1b and Tables S14–16 and Fig. S8). Protein-coding genes showed focused distribution and high expression levels on chromosome arms, on which highly variable levels of DNA methylation were also observed (Fig. 1b). Furthermore, 15 357 genes were annotated with gene ontology (GO) information (Fig. S9; Table S16). Among these, 1703 genes (∼5.21%) encode for transcription factors (TFs) in 69 families while 401 genes (∼1.23%) encode for transcriptional regulators in 24 families (Table S17). We predicted 557 tRNAs in 24 families, 78 rRNAs in 4 families, 71 miRNAs in 20 families and 4254 pseudo-genes (12 490 896 bp or 2.58%; Table S18).

### The BT genome features a high proportion of allelic sequence polymorphisms

Rose genomes are highly heterozygous [9–14]. Therefore, we next examined sequence polymorphisms within the BT genome. We found 2.3 million SNPs (0.44% of BT genome), in which 1.45 million were transitions and 0.85 million were transversions (Table S19, S20 and Figs S10–S12). About 99.7% of the SNPs were heterozygous, with half in intergenic regions. These SNPs resulted in loss/gain of their start or stop codons in 5204 genes. We further identified 0.51 million indels, in which 520 lead to loss/gain of start/stop codons. Around 44.38% indels were located in intergenic regions (Table S21). These sequence polymorphisms will be valuable in investigation of allele-specific expression, epigenetic regulation, genome structure and in evolutionary analyses of roses.

### BT diverges significantly from the haploOB genomes

To understand genetic variation associated with the contrasting traits between BT and OB, we identified 95 synteny blocks covering 87.59% of BT (464.31 Mb) or 80.59% of haploOB (415.5 Mb) genomes. We detected 20 inversion events (covering 1464 genes of BT or 1718 genes of haploOB) (Fig. 2a, Table S22 and S23). We detected 7.31 million SNPs and 5.16 million one-nucleotide indels as well as 20.27 Mb large structural variants including indels. These included sizes above 50nt (3171x events) and tandem (458x) or repeat (5146x) expansion/contractions (Fig. 2c and d; Fig. S13; Table S24). We detected 17 034 genomic rearrangement events (∼65.8 Mb) not randomly distributed along chromosomes (Fig. 2d and Table S25). Of greater interest, genes related to DNA integration and metabolic processes were significantly enriched in copy-gain-regions (Table S26). This was in line with the strong requirement of maintaining genome stability during frequent hybridization and genomic rearrangement during the long-term domestication of OB [12]. Taken together, a strong sequence divergence occurred between the two genotypes, and this divergence could be used for genetic dissection of important rose traits.

### BT experiences significant gene family expansion/contraction profiles

We identified 11 593 homologous gene families shared among BT, haploOB and in the genera *Prunus*, *Pyrus*, *Fragaria* and *Malus*. We detected 1320 gene families with 1999 genes specific for BT, and found 2593 families with 4586 genes unique for haploOB (Fig. 2e). The BT-specific genes were significantly (FDR < 0.05) enriched in GO terms for nucleic acid binding, RNA-dependent DNA biosynthesis and DNA integration processes but proved significantly less for oxidation-reduction genes (Table S27). By comparison, the haploOB-specific genes were functionally over-represented in DNA metabolic, organic substance metabolic, hetero- and organic-cyclic compound binding. These results suggested that BT and OB harbored different and specific gene profiles. However, BT and OB had a very similar within-genome duplication pattern (Fig. S14). Rose and strawberry shared an ancient whole-genome-duplication (WGD) event, but without the recent one featured by apple (Figs S15 and S16) [29].

To understand the phylogenetic position and identify the expanded/contracted gene families in BT, we searched for the conserved orthologous gene families in genomes of BT, haploOB, *Malus domestica* Borkh*.*, *Fragaria vesca*, *Prunus avium* L., *Prunus persica* (L.) Batsch., *Pyrus communis* L. and *Rubus occidentalis* L. (all members of the Rosaceae), with *Vitis vinifera* L. (Vitaceae) and *Ziziphus jujube* Mill. (Rhamnaceae) as outgroups. We identified 1 220 single-copy orthologous groups and used them to construct a maximum-likelihood tree (Fig. 2f). BT and OB were sisters and *Fragaria* was the closest genus. Following the fossil calibrations of nodes C1 and C3 for the Rosaceae [30], the estimated divergence time of BT and OB was ∼6.15 MYA (95% highest posterior density: 2.33–15.43 MYA). A total of 978 and 616 gene families were apparently expanded and contracted, respectively, in the BT genome (Fig. 2f). This was significantly different from that of OB (Yates’ Chi-square test, *P* < 0.0001; Fig. S17 and Table S28). Being consistent with its high resistance to black spot [18], BT significantly expanded the *NAC* family genes (146 against 114 in OB; *P* = 0.05; Table S17) especially on clade V (72 in BT and 41 in haploOB, *P* = 0.004; Fig. S18). The lineage-specific expansion and expression divergence of *FAR1/FRS-like* genes correlated well with transition of shoot growth behaviors upon flowering in BT [31]. These corroborated with the fact that BT and haploOB had a high level of sequence divergence and morphological variation because of the time of divergence between them (Fig. 2a–d).

### Genetic patterning of stem prickle density in BTxOB populations

**Figure 3.**
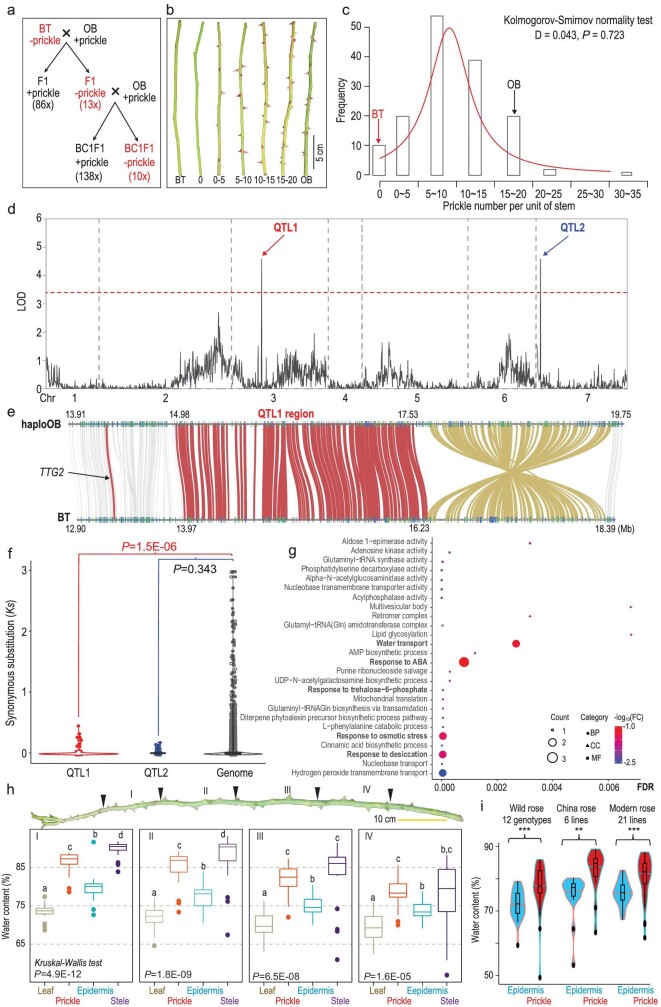
Genetic analysis of stem prickle inheritance in OB × BT populations. (a) Inheritance of prickle distribution on stems of F1 and BC1F1 populations. Plants without prickles on stems indicated with a ‘−’ in red color while a ‘+’ labeled plants have prickles. Numbers in brackets mark the plant number featuring each trait category. (b) Typical phenotypes of prickle density on stems of the parents (BT and OB) and the BC1F1 population. Numbers indicate the range (0, >0–5, >5–10, >10–15, >15–20; the same for (c) of prickle density per 20 cm unit of stem. (c) Normal distribution of stem prickle density in BC1F1 population. The X-axis indicates prickle density per unit of stem length. Y-axis marks the frequency of BC1F1 individuals showing the range of prickles. Arrows show prickle features of both parents (BT in red and OB in dark). (d) QTL analysis for prickle density on shoot in BC1F1 population using multiple QTL mapping (MQM) method. The red line indicates the significance threshold ( = 3.4). Red and blue arrows mark the two QTL over threshold on Chr3 and Chr7, respectively. (e) Syntenic gene pairs (in red lines) between OB and BT surrounding the QTL1. This QTL neighbored a genome conversion (brown lines) between OB and BT. Potential *TTG2* labeled with a dark red line outside of QTL1. Numbers on/under chromosomes show physical positions in Mb. (f) Comparison of synonymous substitutions (*Ks*) between the QTL regions and the rest of the genome. (g) GO enrichment pattern for differentially expressed genes located in the two QTL. X-axis shows FDR values; FC, enrichment fold change against genome level; BP, biological process; CC, cellular components; MF, molecular function. GO terms in bold relate to water usage. (h) Relative water contents are significantly higher in prickles (red) than in epidermis (blue), steles (dark purple) or leaves (brown). Data for one modern rose genotype shown. I, II, III and IV represent every three nodes marked by dark arrow heads. Letters (a, b, c and d) above each boxplot indicate pairwise significances (*P* < 0.01; non-parametric Wilcoxon test) for eight biological replicates. (i) Prickles (red) with relatively higher water contents than epidermis (blue; *P* < 0.001, Student's t-test) in a collection of randomly selected roses (12 wild rose, 6 China rose and 21 modern cultivated genotypes). ^*^^*^^*^, *P* < 0.001; ^*^^*^, *P* < 0.01. For (h) and (i), prickle number does not co-vary with water content for either prickle, epidermis or stele.

We scored stem prickle density in the 99-individual F1 and 148-individual BC1F1 populations. Both were generated via crossing the prickle-free BT and prickly OB (Fig. 3a and Figs S19–S21) [22,25]. In F1s, the number of prickle-free vs. prickly individuals was 13 : 86, a ratio not deviating from 1 : 7 (three loci hypothesis, Chi-square test, *P* = 0.96) or 1 : 15 (four loci, *P* = 0.43). The BC1F1 plants, based on back-crossing one prickle-free F1 individual to a prickly OB, showed a normal distribution of prickle density (Kolmogorov-Smirnov normality test, D = 0.043, *P* = 0.723; Fig. S22). Ten plants were prickle-free and the remaining 138 had prickles (Fig. 3b and c). Stem prickle density is thus best interpreted as a quantitative trait regulated by three to four loci, with ‘being prickly’ as the incomplete dominant (Fig. S23).

### Stem prickle density is regulated by two main QTL

We conducted a QTL analysis using the 2160 SNP markers for the BC1F1 population [25]. Three and two QTL were identified using interval mapping (IM; Fig. S24) and multiple QTL mapping (MQM; Fig. 3d) methods, respectively. The QTL1 (the logarithm of the odds ratio, LOD = 4.59; 12.3% variance explained) and QTL2 (LOD = 4.58; 12.4% variance) were identical (LOD threshold 3.4). An additive effect was identified for OB alleles in both QTL1 (3.98) and QTL2 (4.23). QTL1 was located on Chr3 next to a genome inversion event (Fig. 3e). This 2.26 Mb QTL (13.97–16.23 Mb) harbored 153 genes in BT and covered a 2.55 Mb fragment (14.98–17.53 Mb; 254 genes) in the haploOB genome. QTL1 was close to, but did not co-locate with, the QTL regions regulating continuous flowering, double flower or self-incompatibility traits (Table S29) [14]. However, this QTL was indeed within the ∼15 Mb segment linked with prickle density on stems as reported previously by HibrandSaint-Oyant *et al.* [14] and Zhou *et al.* [32] (Table S30). QTL1 had 117 syntenic genes between BT and haploOB. The *TTG2-like* (Rw3G013440/Chr3G0468221), a candidate proposed previously [14], was located ∼0.6 Mb outside of QTL1 vicinity. QTL2 was on Chr7 with 60 syntenic genes between BT (BT, 19.03–20.28 Mb, 73 genes) and OB (haploOB, 21.23–22.34 Mb, 65 genes; Fig. 3d; Fig. S25). The weak QTL3 was located at the beginning of Chr7 (BT, 0.98–2.64 Mb, 204 genes; haploOB, 1.08–2.73 Mb, 211 genes; 177 syntenic, Figs S26 and S27). QTL2 and QTL3 were not identified previously in genetic analyses with F1 populations [14,32], suggesting that populations in different generations might provide different powers in genetic analyses.

Genes in the QTL1 and QTL3 regions showed a significant reduction in synonymous substitutions (*Ks*) compared with the rest of the genome (Fig. 3f, Fig. S27, Data S1 and Table S31). Additionally, the genes in the QTL1 region displayed a lower level of non-synonymous to synonymous substitution ratios (*Ka/Ks*) compared with the rest of Chr3 (Fig. S28 and Table S31), implying a purifying selection. A detailed examination of the syntenic genes in the QTL regions showed that genes involved in water usage and desiccation responses were significantly and differentially expressed in the young shoot tips (<0.5 cm in length) between BT and OB (Fig. 3g and Fig. S29). Two genes encoding for aquaporin PIP2s, known factors involved in coordination of cell growth and drought adaptation [33,34], featured substantial expression reduction in shoot tips of OB than in BT (Fig. S30). Our results raised a possibility that genes for prickle patterning might hitchhike on the sequences related to physiological adaptations for water accumulation and storage, or vice versa. Prickles may have a relatively high water content.

We measured the relative water content in prickles, epidermis, steles and leaves at different sections of the stems of two genotypes (*R.* ‘Cinderella’, and *R.* ‘Tianshan Xiangyun’; Fig. 3h and Figs S31–S34). Prickles always had higher water content than epidermis and leaves in the four sections of seasonal shoots. In contrast, relative water content dropped sharply in prickles on older shoots, while maintaining similar levels in leaves compared with young shoots (Fig. S32 and Table S32). This modifies the original hypothesis. Prickles may serve as water storage structures at early phases, graduating to defense mechanisms with stem maturation. Prickles might have different functions over the life span of these woody plants. A further examination of water contents in prickles and epidermis for a collection of 39 lines (12 wild, 6 China roses and 21 modern genotypes) revealed a similar pattern (Fig. 3i, Figs S35 and S36, and Table S33). Taken conjointly, prickle patterning may hitchhike on the ‘guild’ of genes involved in water maintenance in rose stems [18]. Consequently, prickles remain absent in natural populations of *R. wichuraiana* as shrubs grow in moist, mesophytic habitats with monsoon climates [17,18]. Additionally, a preliminary phylogenetic analysis with chloroplast genome sequences indicated independent origins of prickle-free roses (Table S34 and Fig. S37).

### Expression variation of candidate genes in QTL and trichome GRN associates with stem prickle patterning

**Figure 4.**
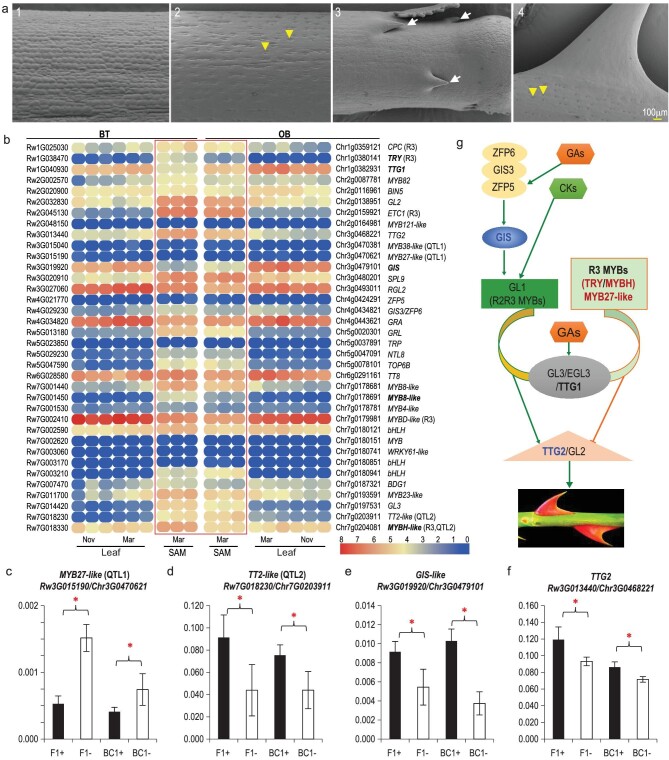
Identification of candidate genes involved in stem prickle patterning. (a) SEM analysis of early (1 and 3) and older (2 and 4) stems for BT (1 and 2) and OB (3 and 4). Prickles and stomata are marked with white arrows and yellow arrow heads, respectively. (b) Expression heatmap of genes involved in prickle-initiation GRN. The bar at the right bottom marks the normalized FPKM values for both BT and OB (red denotes high expression and blue indicates low expression). Names in bold mark genes showing minimum two-fold changes at *P* < 0.05 (Student's two tailed t-test). R3 type MYBs indicated with ‘R3’ in brackets. Genes in QTL marked in brackets. SAM Mar (in red box), young shoots in March; leaf Mar/Nov, with expanding leaves in March/November. (c–f) Expression comparisons for candidate genes (*MYB27-like*, c; *TT2-like*, d; *GIS-like*, e; *TTG2-like*, f) in F1 and BC1F1 pools with (F1+ or BC1+) or without (F1− or BC1−) prickles on stem. (g) A simplified GRN regulating rose prickle initiation based on information from *Arabidopsis*.

Rose prickles are deformed plant trichomes [16] and the responsible GRN has been studied in *Arabidopsis* [1,5,35–38]. Our scanning-electronic-microscopy (SEM) analyses revealed that, in contrast to the normal initiation of prickles and stomata development on OB shoots, BT maintained the ability to develop stomata but lacked prickle initiation (Fig. 4a). Therefore, we identified potential candidate genes involved in the trichome GRN, and focused on the MBW-complex, that is the MYB-, bHLH- and WRKY-like transcription factors in the QTL regions [1,5,35,37,38]. In *Arabidopsis*, this MBW complex involves the physical interaction between GL3 with GL1 and TTG1, respectively, to initiate trichome formation. *TTG2* acts genetically downstream of this complex while sharing partial activity with *GL2* to initiate trichome development [36]. A complete loss of *TTG2* and *GL2* activity caused the failure of trichome initiation [39]. Single repeat R3-MYBs act as negative regulators of trichome development via competition with the R2R3-type MYBs to form a repressor complex.

*Arabidopsis* has ∼150 trichome-related genes involved in initiation, development, birefringence and other processes (Data S2). We identified 240 BT homologous genes, with 10 and 1 being CPG (OB gain compared to BT; 1314 genes) and CPL (OB-lost; 193 genes), respectively. Among these, the OB-lost but BT-featuring *Rw2G037200* (a duplication of *Rw2G037100*) encoded potentially a TT2-like protein with the At5G35550, which encoded for TT2, as the closet homolog in Arabidopsis [40]. However, both genes were expressed at very low levels in both BT and OB (Fig. S38). The remainders were related to trichome branching (Data S2). None of these 11 genes was in the vicinity of QTL region. We next focused primarily on those 29 genes known to regulate trichome initiation and development (Fig. 4b, Data S3 and S4). In comparison to trichome GRNs of *Arabidopsis*, cucumber and cotton, we detected no presence-absence polymorphisms of genes between BT and OB. These results indicate that the trichome GRN is highly conserved among these roses [3,5,6,41].

We found four *MYB*s (*Rw3G015040/Chr3g0470381*, *Rw3G015190/Chr3g0470621* for QTL1; *Rw7G018230/Chr7g0203911* and *Rw7G018330/Chr7g0204081* for QTL2). We identified five *MYB*s (including a homolog of *WER*/*GL1/MYB23-like*), three *bHLH*s and one *WRKY* in QTL3 (Data S3 and S4; Table S35; Figs S38–S43). We detected no significant sequence variation in the means of *Ka*/*Ks* change between these orthologous genes. We found no lineage-specific expansion/contraction of these three gene families. The interpretation here is that the regulation of gene expression, not the encoding potential, of these trichome-related genes may underlie regulation of prickle-free stems in BT. Indeed, we detected five genes with at least two-fold significant expression changes in very young shoots including apical meristems between BT and OB (<0.5 cm; Fig. 4b) [20]. The expression of positive regulators *GIS* and *TTG1* decreased, while the negative regulators R3-type *MYB*s (*TRY-* and *MYBH-like*) and *MYB8-like* increased in BT compared to OB (Fig. 4b and Data S4). A qRT-PCR assay confirmed the significant expression variation of *MYB27-like* in QTL1 (Rw3G015190/Chr3G0470621), *TT2-like* in QTL2 (Rw7G018230/Chr7G0203911), *GIS-like* (Rw3G019920/Chr3G0479101) and *TTG2-like* in both F1 and BC1F1 pools with or without prickles (Fig. 4c–f). Both MYB27-like and TT2-like TFs are proposed to regulate anthocyanin synthesis in *Petunia* and *Arabidopsis* via modulation of the MBW-complex [42–44]. However, their functional roles in trichome development need further investigation. Acting downstream of the R3-MYBs, GIS promoted trichome development via transcriptional regulation of genes involved in the MBW-complex [45,46]. Featuring strong sequence variations in the upstream transcriptional regulatory regions (Fig. S45), the WRKY *TTG2* worked downstream of the MBW-complex but partially independent of *GL2*. This leads to regulation of trichome patterning and seed-coat anthocyanin biosynthesis [36,39]. It was a candidate for stem prickle patterning in roses [14]. Interestingly, variations in *cis-*regulatory mutations in MYB TFs, *TCL* and *TRY*, together with a *GL1* mutation, triggered the trichome development on *Arabidopsis* fruits. These variations were significantly associated with low spring precipitation thus may contribute to climate adaptation [47]. Taken together, we propose that gene expression variation of these candidates underlies prickle-development variation between BT and OB (Fig. 4g).

## CONCLUSION

We generated a chromosome-level genome assembly for the heterozygous diploid BT, and identified the complex inheritance pattern of stem prickles.

Production of the diploid BT genome sequence provides us with a foundation and novel resources to study rose biology and mine molecular markers associated with important traits. This offers targeted strategies for breeding new rose varieties and for studying genome evolution in the commercially important family, Rosaceae. For long-term benefits, according to the fairy tales of Jacob L.K. Grimm and Wilhelm C. Grimm (1812, *Little Brier-Rose*) and Oscar Wilde (1888, *The Nightingale and the Rose*), respectively, landscapes of prickle-free roses should reduce fatalities in populations of male royalty and birds in the genus *Luscinia.*

## METHODS AND MATERIALS

Detailed descriptions of methods are available as Supplementary data.

## Data availability

All data supporting the results of this study are included in the manuscript and its additional files. Genome assembly and annotations were deposited in the NCBI BioProject under accessions PRJNA542225.

## Supplementary Material

nwab092_Supplemental_Files
