# Supplementary material for: Rose without prickle: genomic insights linked to moisture adaptation
Source: Natl Sci Rev. 2021 May 22;8(12):nwab092. doi: 10.1093/nsr/nwab092 (PMC8694671; doi:10.1093/nsr/nwab092)
Supplement: nwab092_Supplemental_Files [file nwab092_supplemental_files.zip › Methods and Materials.pdf]

A genomic link in China roses: and they all lived prickly but water deficient ever after?

Mi-Cai Zhong [1,4], Xiao-Dong Jiang [1,4], Guo-Qian Yang [2], Wei-Hua Cui [1,4], Zhi-Quan Suo [1,4], Wei-Jia Wang [3], Yi-Bo Sun [1,4], Dan Wang [1,4], Xin-Chao Cheng [5], Xu-Ming Li [5], Xue Dong [1,2], Kai-Xue Tang [3,*], De-Zhu Li [1,2,*], Jin-Yong Hu [1,*]

Methods and Materials

Plant materials and prickle density scoring

R. wichuraiana 'Basye's Thornless' (BT) plants were grown in a glasshouse without additional lighting in Kunming. The BC1F1 population between *R. chinensis* 'Old Blush' (OB) and BT was developed as described previously [1]. F1 progeny (n=98) was produced by crossing the OB and BT plants propagated vegetatively. One prickle-free F1 plant featuring five petal flowers was pollinated with OB pollen to produce the BC1F1 progeny (n=150), of which 148 individuals were included in the mapping population [2]. Prickles were scored from the 6th-24th nodes on 4-6 main stems from at least 3 individuals for each genotype. Prickle density was arbitrarily defined as the prickle number on 20cm stems.

Scanning electron microscopy (SEM) analysis of stem prickles

SEM analysis was carried out for BT and OB young stems (shorter than 1cm) with a Zeiss Sigma 300 microscope (Germany). At least five stems from independent individuals for each genotype were examined (Fig. 4a).

Genomic DNA extraction, genome size and heterozygous level estimation

High-quality genomic DNA for sequencing using the Illumina platform (Illumina Inc., San Diego, CA) and PacBio Sequel sequencing (Pacific Biosciences of California, Menlo Park, CA) was extracted from fresh leaf materials of a single BT plant. Genome size of BT was estimated with C-value measurement using a BD *FACScalibur*. Two short-insert libraries (260bp and 360bp) were constructed and sequenced to produce a total of 140.34 Gb of 150 bp paired-end raw reads using the standard protocol provided by Illumina NextSeq 500 platform. Following the removal of low-quality and redundant reads, we obtained 135.19 Gb (96.3%) of clean data (Table S1). All clean reads were subjected to 17-mer frequency distribution analyses with *JELLYFISH* (v1.1.10). This analysis revealed two clear peaks with one at about 120x (heterozygous peak) and the other one at about 240x (homozygous peak). The heterozygosity level of BT was estimated to be about 1.03%. A pilot genome assembly with *SOAPdenovo* pipeline produced approximately 456.86 Mb. Corroborating with its ~65% repeat contents (Table

S7), this assembly contained a very high N rate of 57.98%, indicating that the use of only Illumina reads was not feasible for assembling the genome of BT due to high levels of heterozygosity.

Genome assembly using PacBio and ONT long reads

We sequenced the BT genome with both PacBio and ONT platforms with two 20Kb genome DNA libraries. Around 5.69 million reads (48.77 Gb) with a read of N50 length of 12.83 kb after removing adaptor and low-quality reads were generated with PacBio platform and used for genome assembly. We combined *CANU* (v1.4; the Corrected-Error-Rate parameter was set at 0.025) [3] and *WTBDG2* [4] methods followed by *Quickmerge* analysis [5] to generate the initial assembly. The 47.58 Gb ONT reads with N50 of 21,531 bp were corrected with themselves and Illumina reads using *NextPolish* [6]. The polished ONT reads longer than 60 kb were used to fill the gaps of PacBio contigs by *TGS-GapCloser* [7]. The resulting assembly was polished again using PacBio reads and Illumina reads that had been used for survey analysis.

Chromosome level assembly using Hi-C approach

To generate physical scaffolds of BT genome, we constructed Hi-C fragmented libraries of ~350bp and generated 19.48Gb clean reads via Illumina NovaSeq-6000 platform. After mapping of the Hi-C reads and assignment to restriction fragments [8], we used aligner *BWA* (version 0.7.10-r789; *aln*; other parameters, default) and identified the unique reads (~52.28%). We mapped them to the above BT assembly. About 81.03% of the uniquely mapped reads were valid interaction pairs (28.07M). A total of 1,967 contigs was generated after error correction. *LACHESIS* (parameters: *cluster_min_re_sites=52; cluster_max_link_ density=2; cluster_ noninformative _ratio=2; order_min_n_res_in_trun=46; order_min_n_res_in_ shreds=42*) [9] was used to assign the order and orientation of each contig. Finally, 521.32 Mb (99.00%) of the assemblies were anchored onto the 7 pseudomolecules, of which 481.76 Mb (92.41%) was ordered, oriented, and evaluated with *HiC-Pro* (v2.10.0) with manual correction for the contigs showing potential error in order/orientation.

Genome quality evaluation

The BT genome was evaluated for completeness and quality with the following approaches. First, we subjected the assembly to Benchmarking Universal Single-Copy Orthologs (BUSCO, v4.1.0, eudicots_odb10) [10]. Second, the quality was assessed using two independent genetic maps. We used the integrated K5 map based on 172 tetraploid individuals [11] followed by a recently developed map for 150 diploid individuals for a BC1F1 population between OB and BT (OB x BT map) [2]. Genetic markers were aligned against the assembled BT genome with *blastn* (*e-value<=1e-5*) and association rates were evaluated with Pearson's correlation test in *R*. Third, we aligned the BT assembly to both the Raymond's and Hibrand Saint-Oyant's haploOB genome

sequences using *MUMmer4* pipeline (*nucmer –t 20, -L 12000*) [12]. Fourth, we judged the assembly quality by mapping our Illumina and PacBio reads back to the genomes. We estimated the quality value (QV) with *FreeBayes* as described [13]. Finally, the BT assembly was examined for its *LTR assembly index* (*LAI*) following procedures suggested [14].

Annotation of repeat sequences in BT genome
To dissect the molecular makeups of the BT genome, we constructed a *de novo* repeat database with *LTR FINDER* v1.05, *RepeatScout* v1.0.5, *PILER-DF* v2.4 programs. We classified these repeats with *PASTEClassifier* and merged them with the *Repbase data*. *RepeatMasker* (version 4.0.6) was applied to identify repeat sequence against the *de novo* repeat library.

Gene annotation
Gene annotation of the BT genome was completed using *de novo*, homology-based, and transcriptome-based predictions. We employed *Genscan*, *Augustus* v2.4, *Glimmer HMM* v3.0.4, *GeneID* v1.4, *SNAP* (version 2006-07-28) tools to predict protein coding genes. We used *GeMoMa* v1.3.1 to identify genes with homology to *Arabidopsis thaliana* (L.) Heynh, *Oryza sativa* L., *Fragaria vesca* L., *Malus domestica* Borkh., and *Pyrus x bretschneideri* Rehder. gene models. We next mapped Illumina transcriptome short reads onto the genome assembly and predicted gene models with the *PASA* v2.0.2, *transDecoder* (http://transdecoder.github.io) and *GeneMarkS-T* approaches. The transcriptome data was produced by Illumina sequencing of a pool of materials including leaves, shoots, flowers and roots and from Li et al. [15]. All gene models were then integrated using *EVidenceModeler* v1.1.1 to generate a consensus gene set.

We performed functional annotation of protein-coding genes via aligning to the NR, GO, KEGG, KOG, TrEMBL databases with *BLAST* (v2.2.31) with an *e-value cutoff of* 10^{-5}. We also used *Blast2GO* software to search the GO and KEGG databases.

Additionally, four types of non-coding RNAs (microRNAs, transfer RNAs, ribosomal RNAs, and small nuclear RNAs) were annotated using the *tRNAscan-SE* and *Infenal* v1.1 against the *Rfam* as well as the *miRbase* databases. *GenBlastA* was also used for pseudogene predication by scanning the BT genome for sequences homologous to the known protein coding genes it contained. *GeneWise* was employed to search for premature stop codons or frame shift mutations in those sequences.

Gene family identification and phylogenetic relationships within the Rosaceae

OrthoMCL (v2.0.9) was applied to identify the conserved orthologous gene families within genomes of BT, OB, *R. multiflora* Thunb., *Malus domestica*, *Fragaria vesca*, *Prunus avium*, *Prunus persica*, *Pyrus communis*, and *Rubus occidentalis* L. *Vitis vinifera* and *Ziziphus jujube* were used as outgroups. A total of 1,220 single-copy orthologous groups was detected and used for sequence concatenation and alignment with *MUSCLE* [16]. The concatenated sequences were then applied in *PHYML* (v20151210) to construct a maximum-likelihood tree with 500 times of bootstrap supports tested. To estimate the divergence time between BT and OB, the C1 and C3 fossil nodes were used for dating [17]. Gene family expansion/contraction analysis along lineages on the phylogenetic tree was carried out with *CAFE* (v4.2).

Whole genome duplication (WGD) history prediction

To examine the WGD history of BT, we first searched for duplicated genes using Large-Scale Genome Alignment Tool (LAST; http://last.cbrc.jp) with default parameters. All *Ks* distribution patterns were performed with the *WGD* tool [18] for BT and apple [19] as well as strawberry [20]. The complete set of paralogues within and between of these genomes were identified by all-vs-all BLASTP with e-value at $1x10^{-10}$. Pairwise co-linearity analyses between BT, apple and strawberry were conducted with i-ADHoRe v3.0 [21]. Within genome synteny analyses were based on the syntenic paralogous families inferred with WGD. The 4DTv values for these species were also obtained via the maximum likelihood method (ML) implemented in CodeML of the PAML package. The distribution of 4DTv values were plotted to show the WGD events in these genomes as described [22].

Identification and phylogenetic analyses of resistant genes and *MYB/bHLH/WRKY-like* genes

To identify the potential homologous genes of Arabidopsis in BT and haploOB, we first obtained the Arabidopsis sequences related to diseases/defense and *MYB/bHLH/WRKY-like* genes. We then applied the *iTAK* pipeline (*-f 6*, with other parameters as default) to look for potential homologous genes in roses [23]. Sequences related to specific gene family were extracted with custom per scripts and aligned using *MAFFET* using default parameters [24]. Phylogenetic reconstruction was performed with Neighbor-Joining method in *RAxML 8.2.11* including 1000 times of bootstrap simulations [25].

Alignment, sequence variation and synteny analyses between BT and haploOB genomes

Genome alignment between BT and haploOBs was performed using *MUMmer* program (v4.0) with parameters *–maxmatch –c 90 –l 40*. The alignments were filtered by delta-filter with parameter *−1*. SNPs. Indels in the two species were extracted with *show-snp* in the one-to-one alignment blocks. The clean Illumina reads were mapped to the BT genome with *BWA* (v0.7.10, *- r789*).

SNPs and small indels within the BT genome were identified using the *Genome Analysis Toolkit* (*GATK*, v4.0.9), *Samtools* (v0.1.19), and FreeBayes. Only the variants supported by at least two tools were used for further analysis. All these variants were annotated using the *ANNOVAR* program. One All-vs-All *blastp* (*e-value <1e-10*) was carried out for each genome pair. Homologous genes were identified using the *MCScanX* with default settings except *gap_penality* as *-3*. Syntenic blocks were defined as the genome region featuring at least ten collinearity genes. Linear parallel plots in *SynVisio* (*https://github.com/kiranbandi/synvisio*) were used to visualize the conserved regions in the collinearity data generated with *MCScanX*. We used default parameters except *e-value* (=1e-5) and *maximum gaps* (=25) with contigs not anchored/oriented on chromosomes ignored.

We applied *minimap2* [26] (*-ax asm5 –eqx*) to generate the local pairwise alignment between BT and haploOB genomes. *SyRI* [27] (*-k -F S*) was used to identify the rearrangement events including inversion region (INV), translocated region (TRANS), copy gain (CPG) and copy loss (CPL) in OB in reference to the BT genome. Data was plotted as rearrangement length per 100 kb of step size in 1 Mb window in *R*.

Linkage map and QTL analysis

A previously published genetic map was used in this study [2]. The map was constructed with JoinMap 4.0 [28] with high quality genotyping data (152 individuals, missing data rate < 6%) developed by RAD-seq. A QTL analysis was performed using *MapQTL* 6.0 software (Kyazma) by combining the linkage map with the phenotypic identification data. A Permutation Test (1000 repetitions, α= 0.05) was performed to identify 3.4 as the LOD threshold for the presence of a QTL. The QTL were identified with both Multiple QTL Model (MQM), and Interval Mapping (IM) methods. Positions with the highest LOD values on the linkage group were taken as the location of the QTL. The interval of 2-LOD was used here as the 95% confidence interval. We performed forward selection with interval mapping followed by automatic cofactor or covariate selection starting with fixation of the cofactor markers at the detected significant LOD peaks, and extended with all markers on a single linkage group till no new cofactor marker was detected. We did this for all linkage groups. We identified candidate genes and analyzed their function with *BLAST* search (*blast+, $e \leq 10^{-10}$*) of the markers near the QTL intervals within reference genomes.

Identification of prickle genes using *Arabidopsis* homologs

We collected 148 genes related to trichome development and formation in the *Arabidopsis* genome (TAIR10). Protein sequences of these genes were extracted and used as seed sequences to *BLASTp* the BT and haploOB genomes (*e-value 10^{-7}*, *minimum identity 0.35, minimum length coverage 0.6*).

Ortholog gene pairs were then identified using *OrthoFinder2* [29] in combination with the *blastp* results and manual correction based on their genomic synteny. To further investigate the relationship of these gene pairs, we constructed a Neighbor-Joining (NJ) phylogeny with *FastTree* 2.1 based on sequence alignments using *ClustalW* 2.1. Phylogenetic position and syntenic information of these gene pairs were combined to examine the sequence variation between orthologous gene pairs by testing the following types of mutations: a) protein and nucleic sequence completeness, b) small insertions and deletions shifting reading frame, c) large frame-preserving insertions (those creating premature stop codons), d) in-frame stop codons. We further validated these mutation events with Illumina reads to minimize sequencing and assembly errors in these gene pairs. Finally, we identified 240 genes in BT potentially related to prickle development and patterning (Data S2). This list was further used to look for genes also fallen in the CPL and CPG lists or in the QTL regions.

Pairwise selection pressure analysis

Non-synonymous (*Ka*) and synonymous (*Ks*) substation rates were tested for selection pressure acting on orthologous genes as based on *MCScan* results (see above). *MUSCLE* was used to generate the nucleotide and protein sequences alignments. They were further used to calculate the pairwise *Ka/Ks* values of *KaKs Calculator2* with the Yang-Nielsen (YN) model [30]. Only those gene pairs featuring a significant deviation of *Ka/Ks* from 1.0 were treated as positively selected genes. *Ks* values in the QTL1 region were plotted in *R* in comparison to other parts of the whole genome.

Gene expression analyses

Fresh leaves and young shoot (shorter than 0.5cm) tissues were harvested and sequenced with Illumina platforms as described by Li et al. [15]. Briefly, the shoot materials with length shorter than 0.5cm were selected according to the SEM analysis shown in Figure 4a. At this time point, OB shoots featured obvious initiation of prickles, while BT shoots did not. In contrast, both OB and BT shoots had normal development and distribution of stomata. For each tissue per developmental stage, three biological replicates were sampled and sequenced. Clean reads were mapped to BT and haploOB genomes with *HISAT2* and assembled with *StringTie* [31]. Uniquely mapped reads (default parameters expect for *--stranded = no*) were used to calculate the relative expression in FPKM with *ballgown* pipeline in *R*. To estimate whether FPKM values can be directly used for comparison of differential expression between OB and BT, we identified 2,229 and 1,847 housekeeping genes in BT and OB, respectively. The mean FPKM values for these genes were around 295.77 (BT) and 284.97 (OB) with standard deviation below 5, indicating that FPKM values were justified to estimate the relative expression. Next, we identified 17,619 collinear gene pairs between BT and OB based on sequence similarity and

synteny. The differential expression of genes in QTL regions were identified with the fold change (FC) as two folds at significantly different level ($p<0.05$). With these criteria, we detected 2,962 genes up/1,489 genes down in BT over OB. Among the 233, 61, and 179 syntenic gene pairs, 22 up/5 down, 14 up/0 down, and 29 up/4 down regulated genes in SAM tissues were identified for QTL1, 2, and 3, respectively. The two *PIP2*s were located in QTL1 and featured differential expression between OB and BT. The data was included in Data S1. The expression pattern shown in Fig. S17, S37, S39, S41 was plotted as heatmaps with iTOL (v4) [32]. The heatmap in Fig. 4b was plotted using *TBtools* with normalized FPKM values using *ZeroToOne* method and scaled with log2 [33].

To evaluate the relative expression of prickle related genes, total RNA was extracted with RNAprep Pure kit (DP441, Tiangen, Beijing) from the epidermis tissue pools of young stems (1-2cm without apex) in F1 and BC1F1 populations. For each population two groups, one prickle-free and one prickly, were collected. For each pool in each group, four to five independent F1 or BC1F1 lines were used. For the prickly group, lines were arbitrarily selected despite of the prickle density. Three to four pools for each group were collected as biological replicates. Once enough materials were collected, the pool was immediately frozen in liquid nitrogen and stored at -80°C deep freezer. After removing DNA contamination, total RNAs were used for reverse transcription with HiScript II Q Select RT SuperMix (R223-01; Vazyme, Nanjing). Quantitative PCRs were performed with ChamQ Universal SYBR qPCR Master Mix (Q711-02; Vazyme, Nanjing) on QuantStudio^{TM} 7 Flex System (ABI). Relative expression of prickle genes was normalized to *UBC* using primers listed in Table S36.

Relative water content measurement and statistical analysis
Two modern rose genotypes (C220 and C336) were first used to examine the relative water content in prickle, epidermis, stele and leaves all derived from the same stem sections. Four sections, with each containing three nodes, on one young stem (one season of growth) *versus* old stems (produced the previous year) were measured. Each genotype contained at least eight shoots. Prickles were quickly sectioned with a razor blade. The fresh weight of sectioned prickles, epidermis, stele samples and leaves, all on the same stem section, were recorded immediately. All tissues were dried for three days at 80 C in an oven supplied with a wind circulator. Dry weight was measured and the relative water content was calculated as (fresh weight – dry weight)/fresh weight. To compare the relative water content in our rose collection we sampled 12 wild species, 21 modern cultivars and 6 China rose genotypes. For each genotype, two to three sections (each containing three nodes) on one shoot were cut and analyzed. Statistical comparisons were performed with *Wilcoxon* and *Kruskal-Wallis* tests in *R* or *Student's t-test*.

GO enrichment and other statistical analysis

Gene ontology enrichment was analyzed by comparing genes with the genome-wide gene lists of BT or haploOB using a custom *perl* script. The p-values generated with a two-sided *Fisher's exact test* were further corrected for multiple comparisons using *Benjamini and Hochberg correction*. Only GO terms with a false discovery rate of less than 0.05 (FDR <0.05) were retained. A non-parametric two-sided *Wilcoxon test* was used in Fig. 3, and *Kruskal-Wallis test* was used in multi-sample comparisons.

Chloroplast genome assembly and phylogenetic analysis

To investigate the origin of prickle-free roses, we identified three genotypes featuring no prickles on their stems out of approximately 200 genotypes growing in the Kunming Botanical Garden (KIB, CAS). These include a genotype from *R. banksiae* W.T.Aiton (f. *lutea*), and two cultivated genotypes (*R*. cv Emira and Star 'n' Strips; Table S34). We generated minimum 15 Gb Illumina reads for both prickle-free and eight prickly genotypes featuring various levels of prickle density on stems. The chloroplast genome sequences were reconstructed for the three prickle-free and eight prickly genotypes following previously published methods [34]. Final genomes were adjusted and annotated using *Geneious* 9.1.4 (https://www.geneious.com), with the BT chloroplast genome as a reference. Sequences of other rose genotypes were obtained from NCBI. *Rubus crataegifolius* Bunge and *Potentilla freyniana* Bornmuller, Mitth. were used as outgroups. Sequences were aligned using *MAFFT*, and input to *RAxML* 8.2.11 for phylogenetic reconstruction using maximum-likelihood method (*GTRGAMMAI* substitution model, 100 times of bootstrap simulation).

References

1. Li, S., et al., *Inheritance of perpetual blooming in Rosa chinensis 'Old Blush'.* Horticultural Plant Journal, 2015. **1**(2): p. 108-112.
2. Li, S., et al., *The development of a high-density genetic map significantly improves the quality of reference genome assemblies for rose.* Scientific Reports, 2019. **9**(1): p. 5985.
3. Koren, S., et al., *Canu: scalable and accurate long-read assembly via adaptive k-mer weighting and repeat separation.* Genome Research, 2017. **27**(5): p. 722-736.
4. Ruan, J. and H. Li, *Fast and accurate long-read assembly with wtdbg2.* Nat Methods, 2020. **17**(2): p. 155-158.
5. Chakraborty, M., et al., *Contiguous and accurate de novo assembly of metazoan genomes with modest long read coverage.* Nucleic Acids Research, 2016. **44**(19): p. 12.
6. Hu, J., et al., *NextPolish: a fast and efficient genome polishing tool for long-read assembly.* Bioinformatics, 2020. **36**(7): p. 2253-2255.
7. Xu, M.Y., et al., *TGS-GapCloser: A fast and accurate gap closer for large genomes with low coverage of error-prone long reads.* Gigascience, 2020. **9**(9).

8. Servant, N., et al., *HiC-Pro: an optimized and flexible pipeline for Hi-C data processing.* Genome Biology, 2015. **16**: p. 11.
9. Burton, J.N., et al., *Chromosome-scale scaffolding of de novo genome assemblies based on chromatin interactions.* Nature Biotechnology, 2013. **31**(12): p. 1119-1125.
10. Simão, F.A., et al., *BUSCO: assessing genome assembly and annotation completeness with single-copy orthologs.* Bioinformatics, 2015. **31**(19): p. 3210-3212.
11. Bourke, P.M., et al., *Multi-environment QTL analysis of plant and flower morphological traits in tetraploid rose.* Theoretical and Applied Genetics, 2018. **131**(10): p. 2055-2069.
12. Marcais, G., et al., *MUMmer4: A fast and versatile genome alignment system.* Plos Computational Biology, 2018. **14**(1): p. 14.
13. Garrison, E. and G. Marth *Haplotype-based variant detection from short-read sequencing*. 2012. arXiv:1207.3907.
14. Ou, S.J., J.F. Chen, and N. Jiang, *Assessing genome assembly quality using the LTR Assembly Index (LAI).* Nucleic Acids Research, 2018. **46**(21): p. 11.
15. Li, S., et al., *Comparative transcriptomics identifies patterns of selection in roses.* BMC Plant Biology, 2018. **18**(1): p. 371.
16. Edgar, R.C., *MUSCLE: multiple sequence alignment with high accuracy and high throughput.* Nucleic Acids Res, 2004. **32**(5): p. 1792-7.
17. Zhang, S.-D., et al., *Diversification of Rosaceae since the Late Cretaceous based on plastid phylogenomics.* New Phytologist, 2017. **214**(3): p. 1355-1367.
18. Zwaenepoel, A. and Y. Van de Peer, *wgd-simple command line tools for the analysis of ancient whole-genome duplications.* Bioinformatics, 2019. **35**(12): p. 2153-2155.
19. Zhang, L., et al., *A high-quality apple genome assembly reveals the association of a retrotransposon and red fruit colour.* Nat Commun, 2019. **10**(1): p. 1494.
20. Edger, P.P., et al., *Single-molecule sequencing and optical mapping yields an improved genome of woodland strawberry (Fragaria vesca) with chromosome-scale contiguity.* Gigascience, 2018. **7**(2): p. 1-7.
21. Proost, S., et al., *i-ADHoRe 3.0--fast and sensitive detection of genomic homology in extremely large data sets.* Nucleic Acids Res, 2012. **40**(2): p. e11.
22. Guo, Z.-H., et al., *Genome Sequences Provide Insights into the Reticulate Origin and Unique Traits of Woody Bamboos.* Molecular Plant, 2019. **12**(10): p. 1353-1365.
23. Zheng, Y., et al., *iTAK: A Program for Genome-wide Prediction and Classification of Plant Transcription Factors, Transcriptional Regulators, and Protein Kinases.* Molecular Plant, 2016. **9**(12): p. 1667-1670.
24. Katoh, K. and D.M. Standley, *MAFFT Multiple Sequence Alignment Software Version 7: Improvements in Performance and Usability.* Molecular Biology and Evolution, 2013. **30**(4): p. 772-780.
25. Stamatakis, A., *RAxML version 8: a tool for phylogenetic analysis and post-analysis of large phylogenies.* Bioinformatics, 2014. **30**(9): p. 1312-1313.
26. Li, H., *Minimap2: pairwise alignment for nucleotide sequences.* Bioinformatics, 2018. **34**(18): p. 3094-3100.
27. Goel, M., et al., *SyRI: finding genomic rearrangements and local sequence differences from whole-genome assemblies.* Genome Biology, 2019. **20**(1): p. 13.

28. Van Ooijen, J., *JoinMap 4, Software for the calculation of genetic linkage maps in experimental populations.* Kyazma BV, Wageningen, The Netherlands, 2006.
29. Emms, D.M. and S. Kelly, *OrthoFinder: phylogenetic orthology inference for comparative genomics.* Genome Biology, 2019. **20**(1): p. 238.
30. Wang, D., et al., *KaKs_Calculator 2.0: a toolkit incorporating gamma-series methods and sliding window strategies.* Genomics Proteomics Bioinformatics, 2010. **8**(1): p. 77-80.
31. Pertea, M., et al., *StringTie enables improved reconstruction of a transcriptome from RNA-seq reads.* Nature Biotechnology, 2015. **33**(3): p. 290-+.
32. Letunic, I. and P. Bork, *Interactive Tree Of Life (iTOL) v4: recent updates and new developments.* Nucleic Acids Research, 2019. **47**(W1): p. W256-W259.
33. Chen, C., et al., *TBtools: An Integrative Toolkit Developed for Interactive Analyses of Big Biological Data.* Molecular Plant, 2020. **13**(8): p. 1194-1202.
34. Cui, W.-H., et al., *The complete chloroplast genome sequence of a rambler rose, Rosa wichuraiana (Rosaceae).* Mitochondrial DNA Part B, 2020. **5**(1): p. 252-253.
