# Supplementary material for: Rose without prickle: genomic insights linked to moisture adaptation
Source: Natl Sci Rev. 2021 May 22;8(12):nwab092. doi: 10.1093/nsr/nwab092 (PMC8694671; doi:10.1093/nsr/nwab092)
Supplement: nwab092_Supplemental_Files [file nwab092_supplemental_files.zip › SupplFigures.NSRrev1.pdf]

A genomic link in China roses: and they all lived prickly but water deficient ever after?

Mi-Cai Zhong [1,4], Xiao-Dong Jiang [1,4], Guo-Qian Yang [2], Wei-Hua Cui [1,4], Zhi-Quan Suo [1,4], Wei-Jia Wang [3], Yi-Bo Sun [1,4], Dan Wang [1,4], Xin-Chao Cheng [5], Xu-Ming Li [5], Xue Dong [1,2], Kai-Xue Tang [3,*], De-Zhu Li [1,2,*], Jin-Yong Hu [1,*]

Fig. S1-S44.

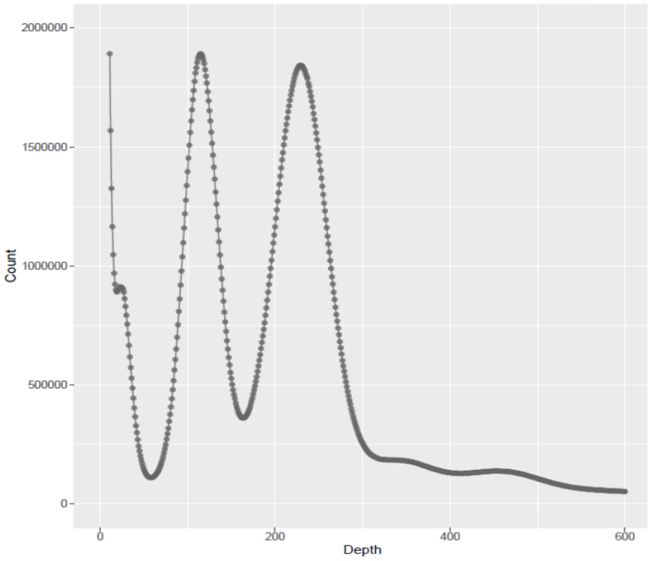

Fig. S1. 17 K-mer distribution of the BT genome.

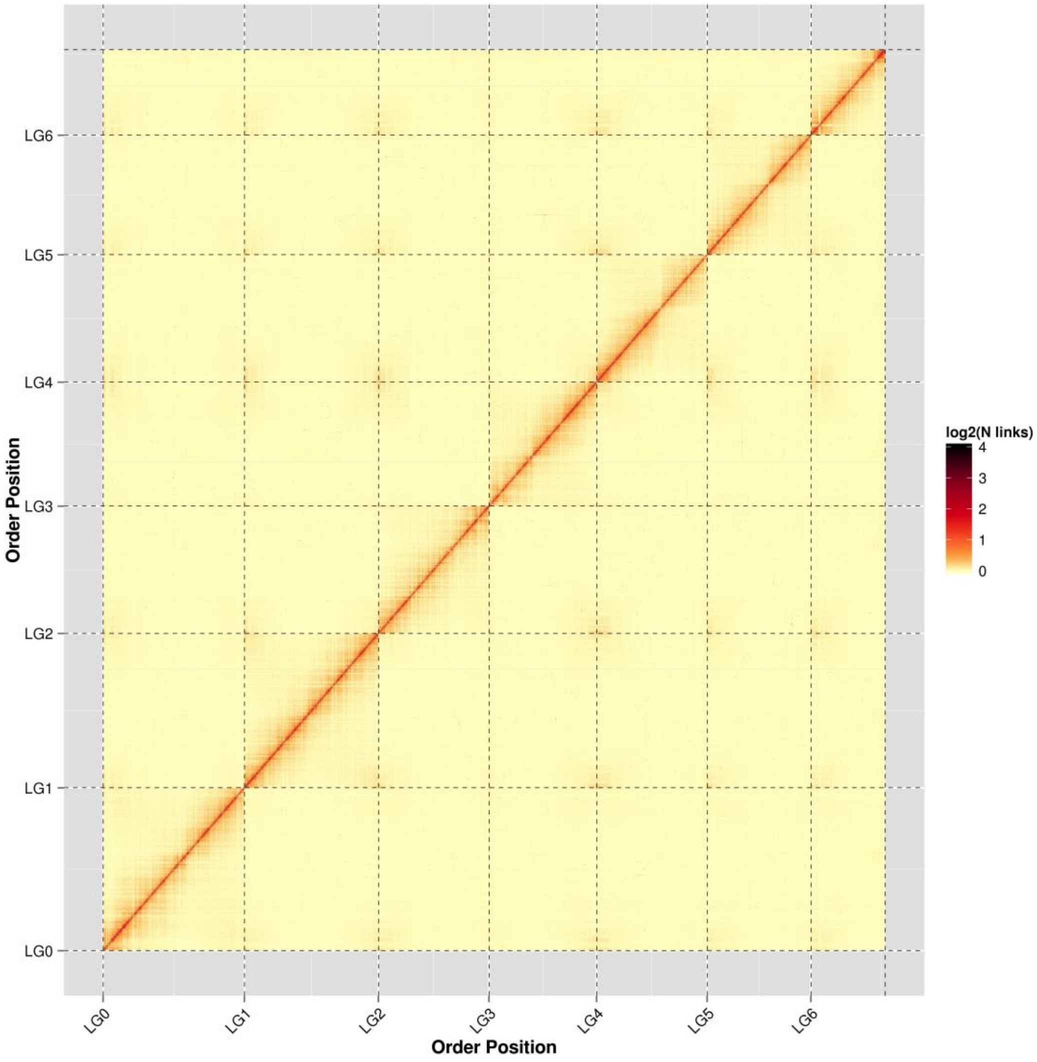

Fig. S2. Interaction frequency distribution of Hi-C links on BT chromosomes.

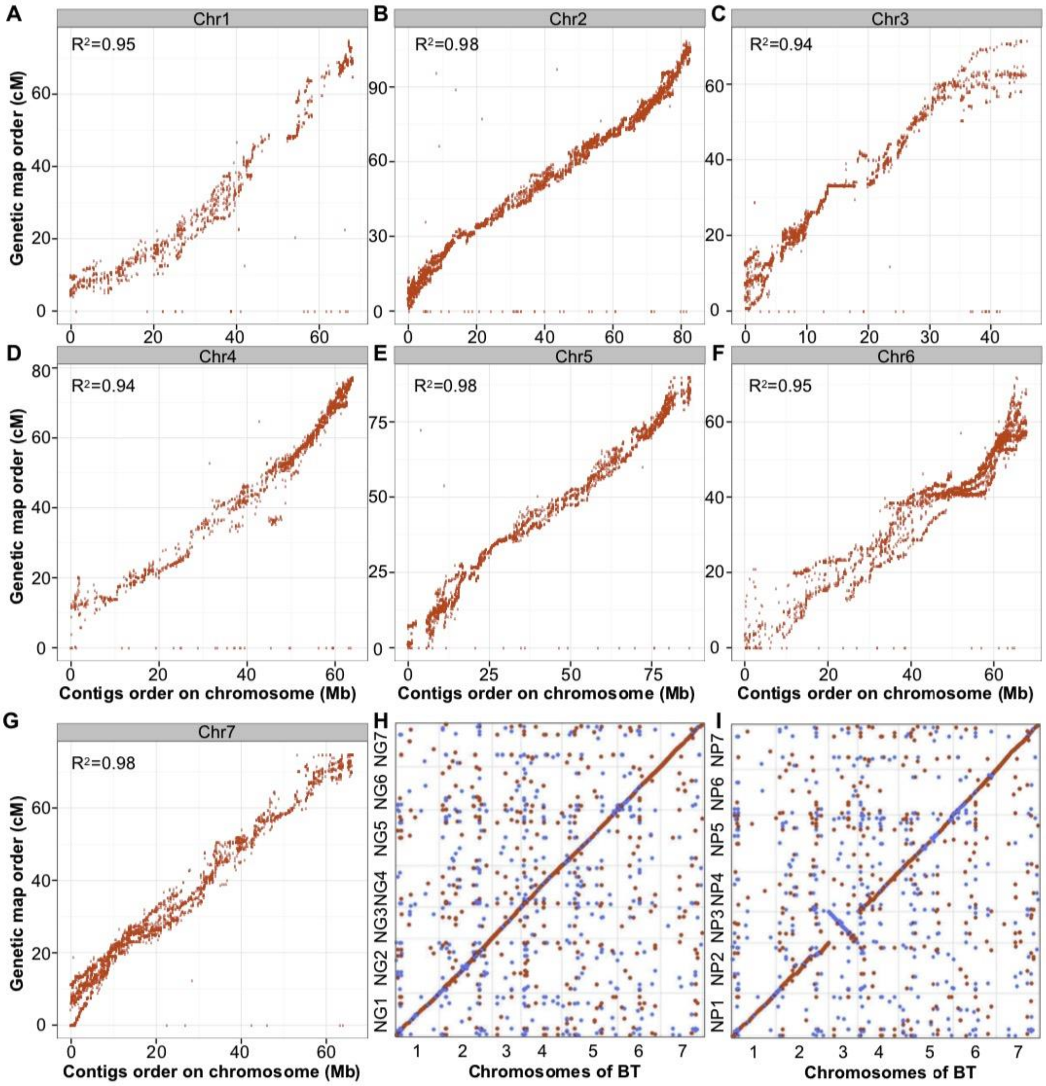

Fig. S3. Collinearity between assembled BT genome and K5 genetic map as well as the haploOB genome sequences. (A) to (G), alignment of the physical sequences of BT chromosomes (in Mb on X-axis) to the K5 genetic map (in cM on Y-axis). R^2 is the Pearson correlation efficiency with all association P-value<1e-16 (Table S8). Collinearity comparison results for BT genome and OBxBT genetic map are given in Fig. S3 and Table S8. (**H**) and (**I**), alignment of the BT chromosome sequences to the haploOB genome reported by Raymond et al. (**H**; Y-axis, designated as NG1-NG7) and Hibrand Saint-Oyant et al. (**I**; Y-axis, marked as NP1-NP7). The analysis was performed with MUMmer4.0.6. Dots closest to the diagonal line represented co-linearity between the two genomes with <7 Kb fragments filtered out. Red and blue dots indicated the collinear and anti-collinear directions, respectively.

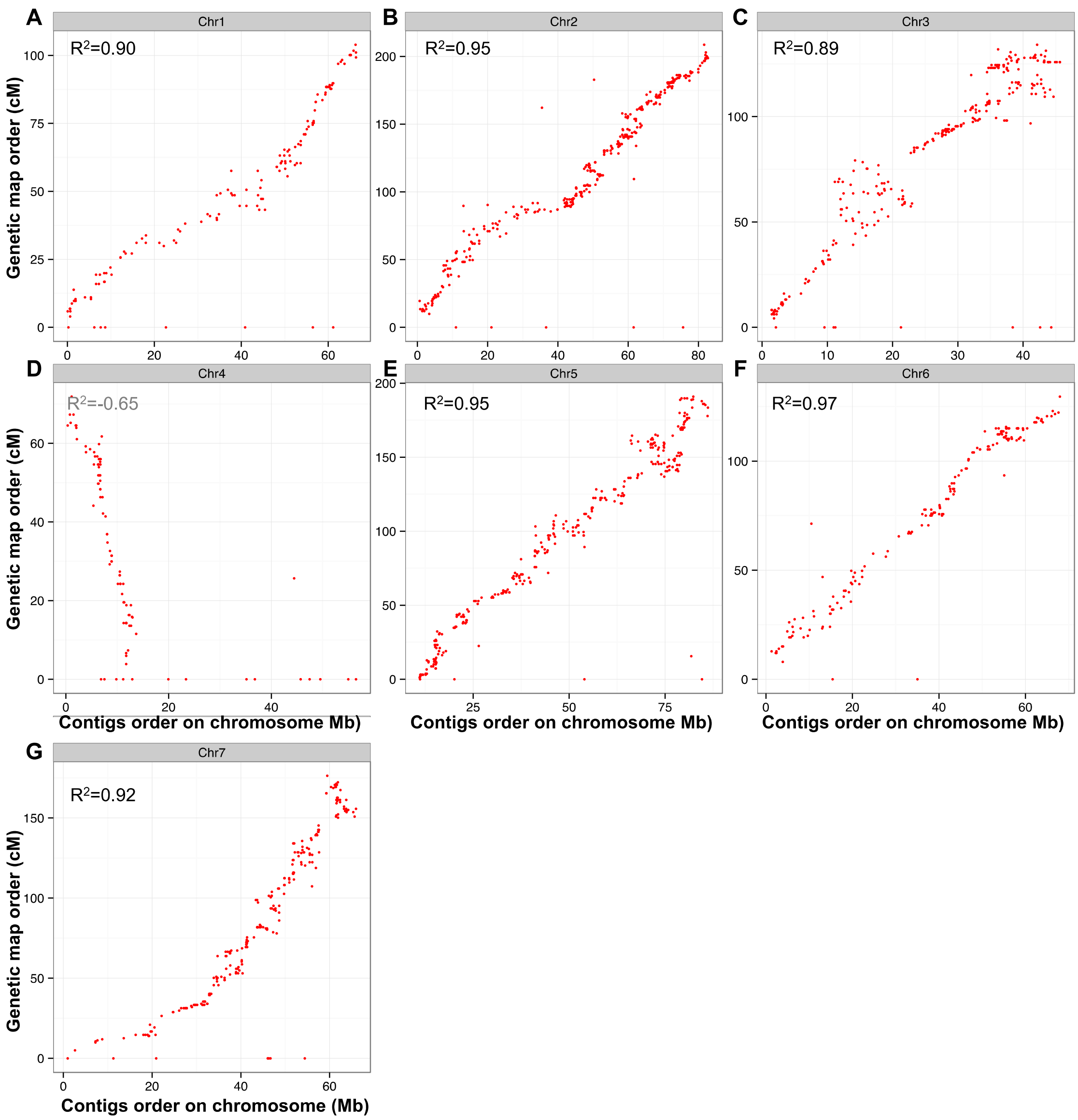

Fig. S4. Collinearity between assembled BT genome and OB x BT genetic map. (A-G) Alignment of the physical sequences of BT chromosomes (in Mb on X-axis) to the OB x BT map (in cM on Y-axis). R2 is the Pearson correlation efficiency with all association being significant (p<1e-50) except Chr4.

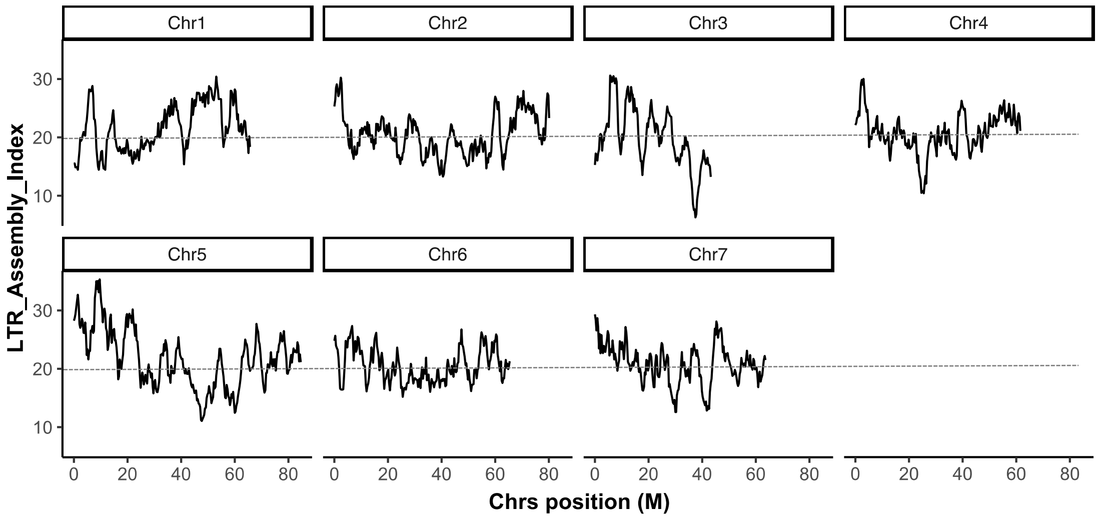

Fig. S5. LTR Assembly Index (LAI) distribution along the seven BT Chromosomes. The gray broken lines indicate LAI=20, the golden reference line, as marked by Ou, Chen and Jiang (2018).

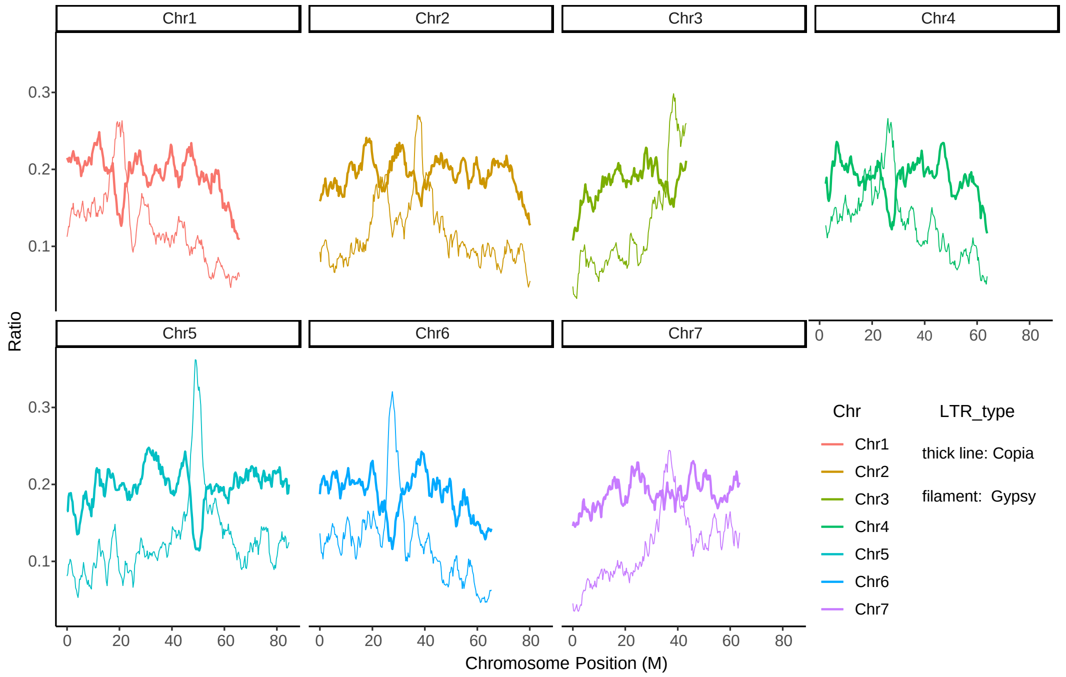

Fig. S6. Distribution of *Copia* (thick line) and *Gypsy* (fine line) types of LTR on the seven BT chromosomes. Note that almost all chromosomes show an antagonistic pattern of *Copia* to *Gypsy* distribution.

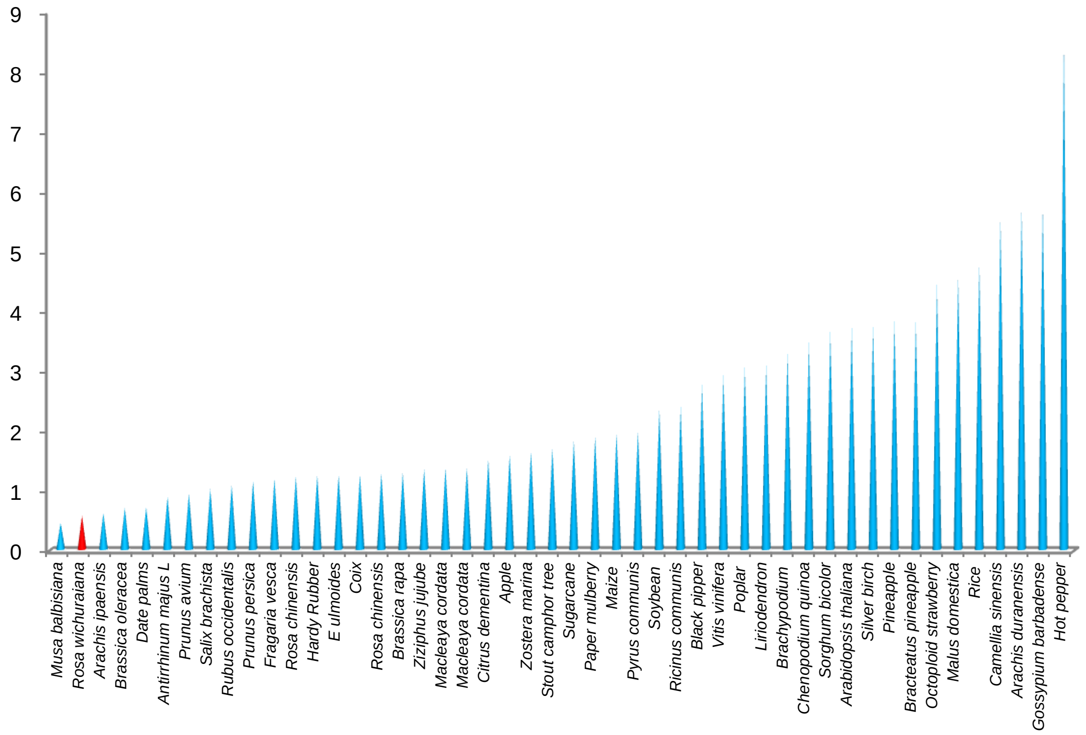

Fig. S7. Ratio (Y-axis) of *Gypsy-to-Copia* types of LTR TEs in BT (in red) and known genomes (See also Table S11).

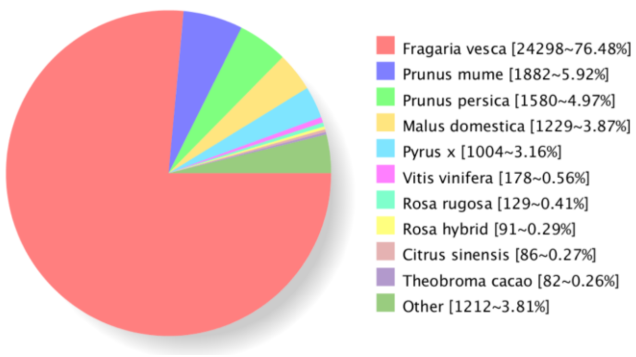

Fig. S8. Homologous gene annotation in NR database against known species. Note that the BT shares the highest number of homologs with *Fragaria vesca*, one of the known closet relatives to *Rosa*.

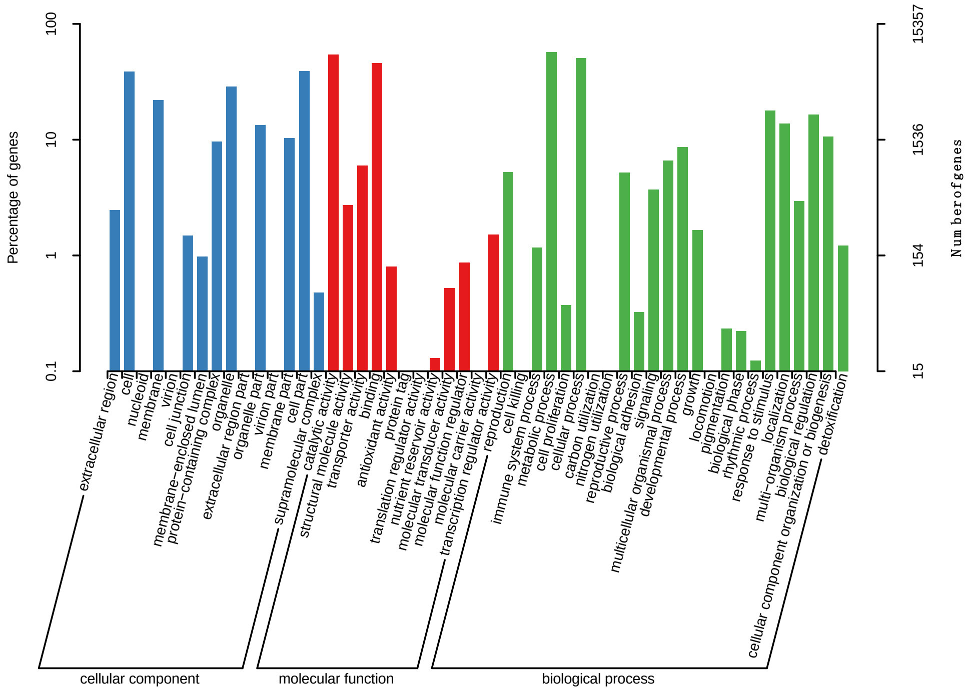

Fig. S9. GO annotation for BT genes.

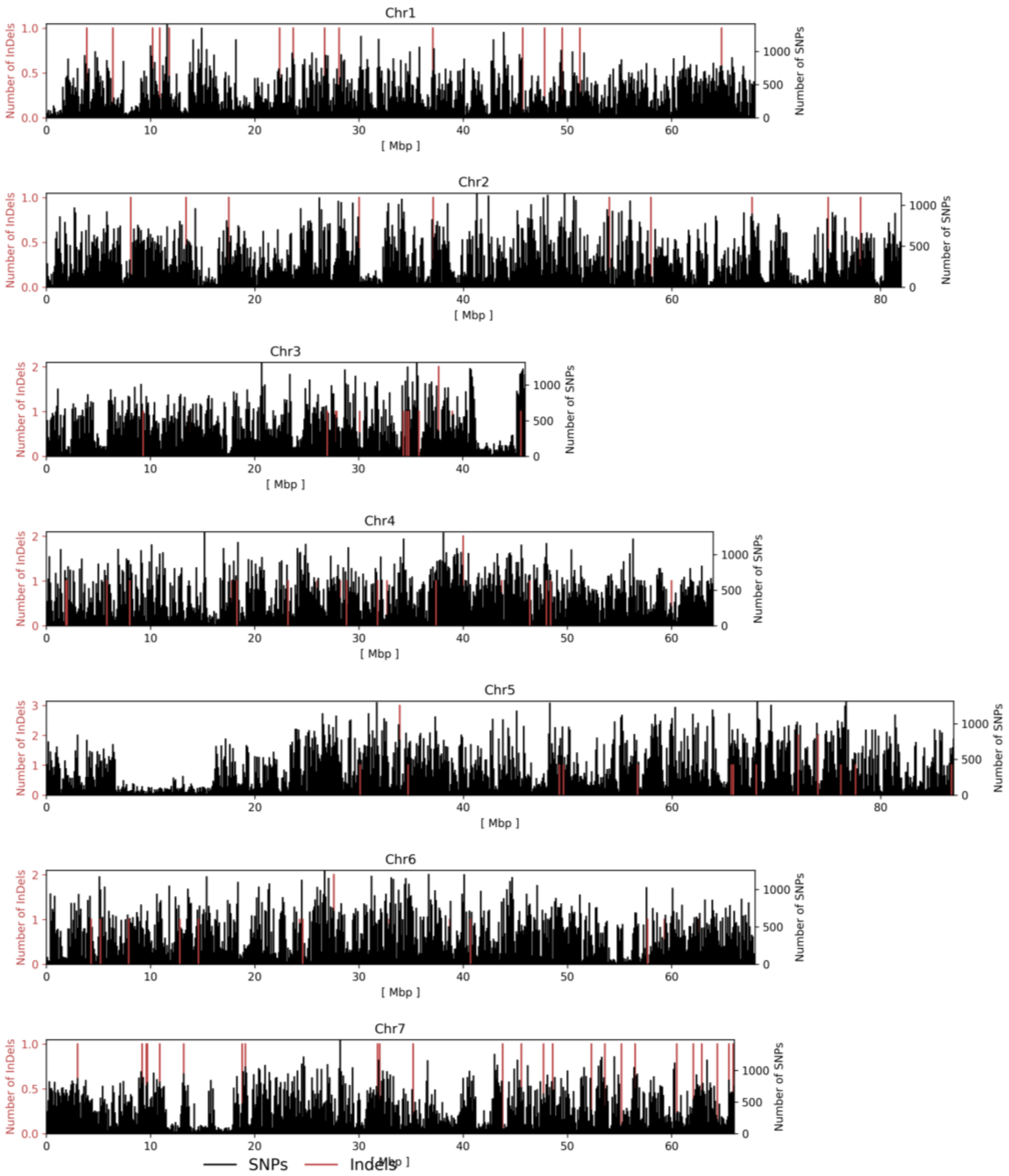

Fig. S10. Distribution of sequence polymorphisms along each BT chromosome. Values on Y-axis gave the numbers of indels (left; in red) and SNPs (right; in black) per 100Kb sequences. Data were generated via re-mapping the Illumina reads to BT genome assembly.

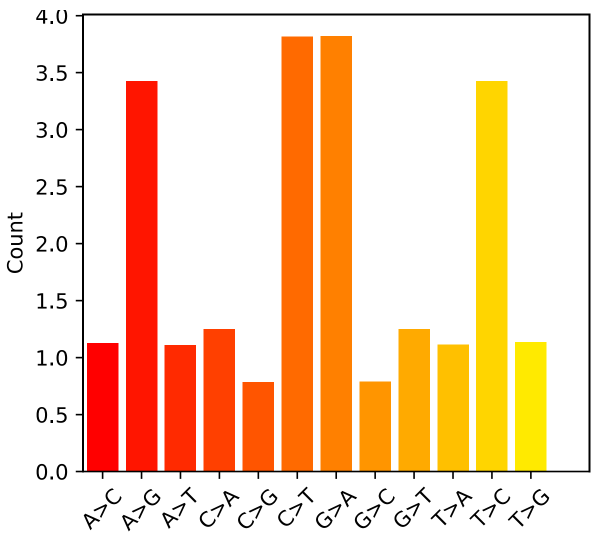

Fig. S11. SNP substitution types in BT genome. Y axis gave the count of SNPs (-1E5) while the X-axis showed the substitution types.

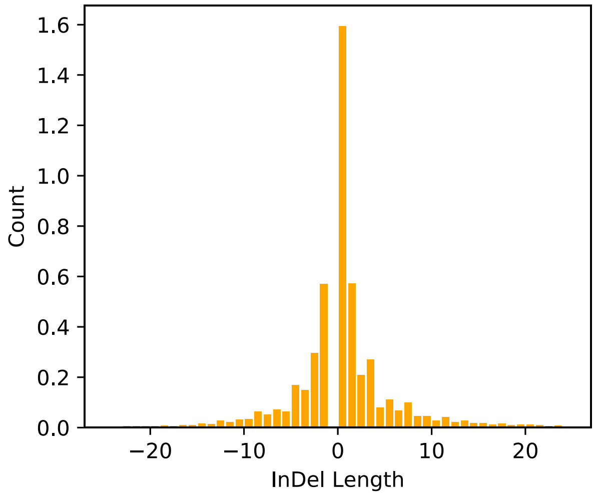

Fig. S12. Indel polymorphisms in BT genome. Y axis gave the count of indels (-1E5) while the X-axis showed the indel length in nucleotides.

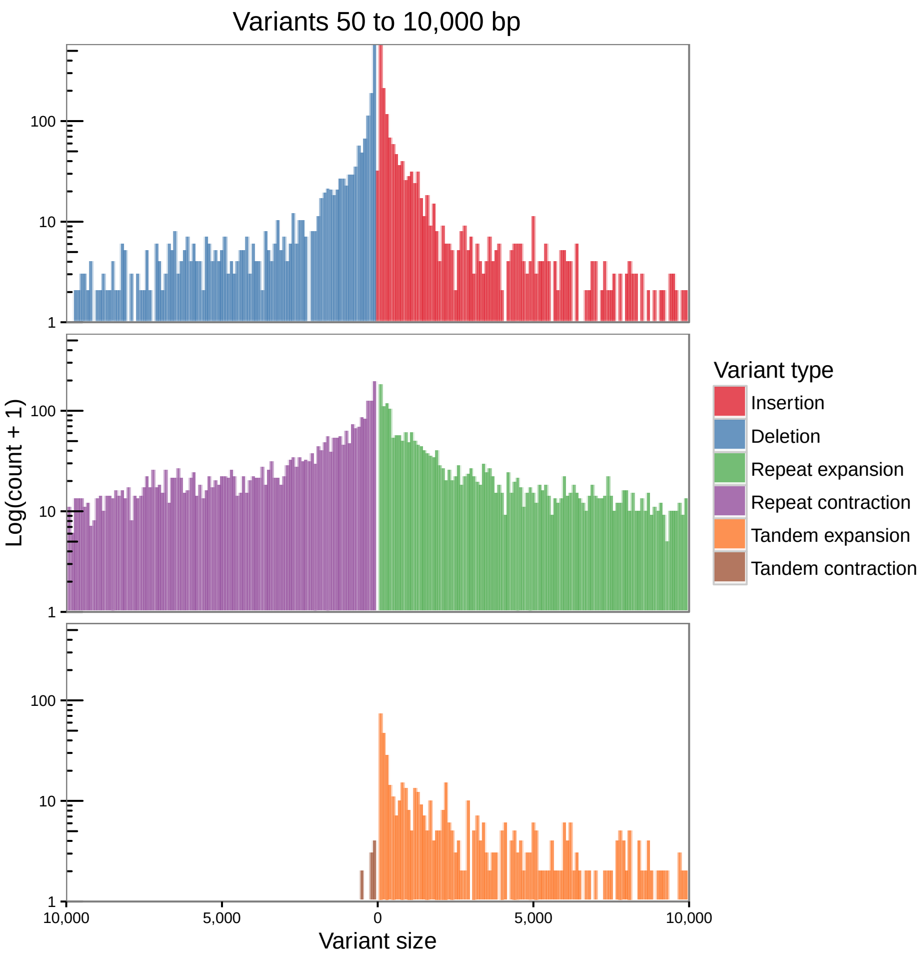

Fig. S13. Large sequence variations between BT and Raymond's haploOB genomes.

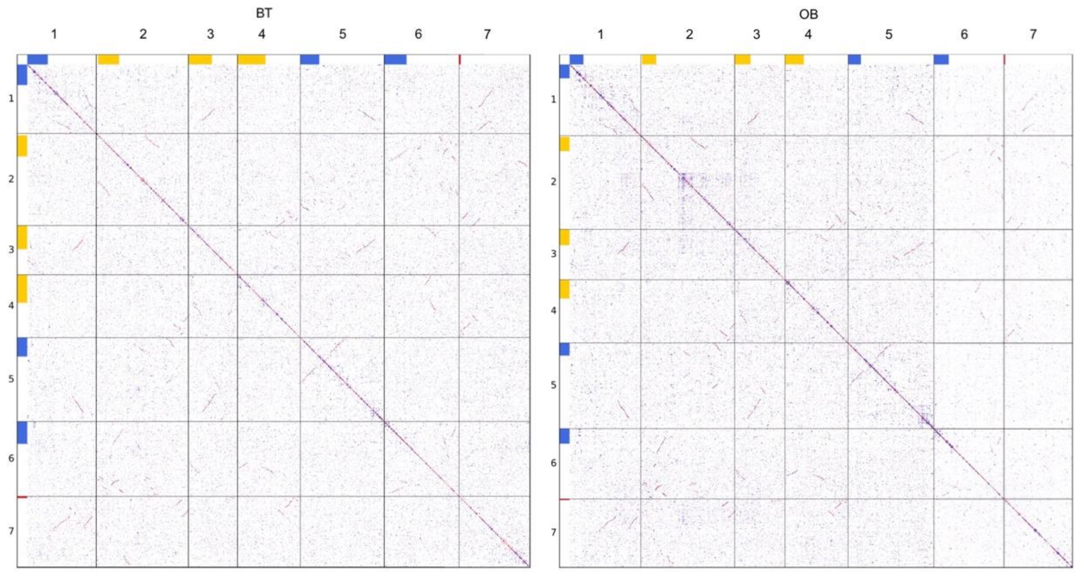

Fig. S14. BT (left) and Raymond's haploOB (right) genomes featured a similar pattern of within genome duplication as shown by a macro-syntenic dot plot.

Numbers on X- and Y-axis indicated the corresponding chromosome number within species.

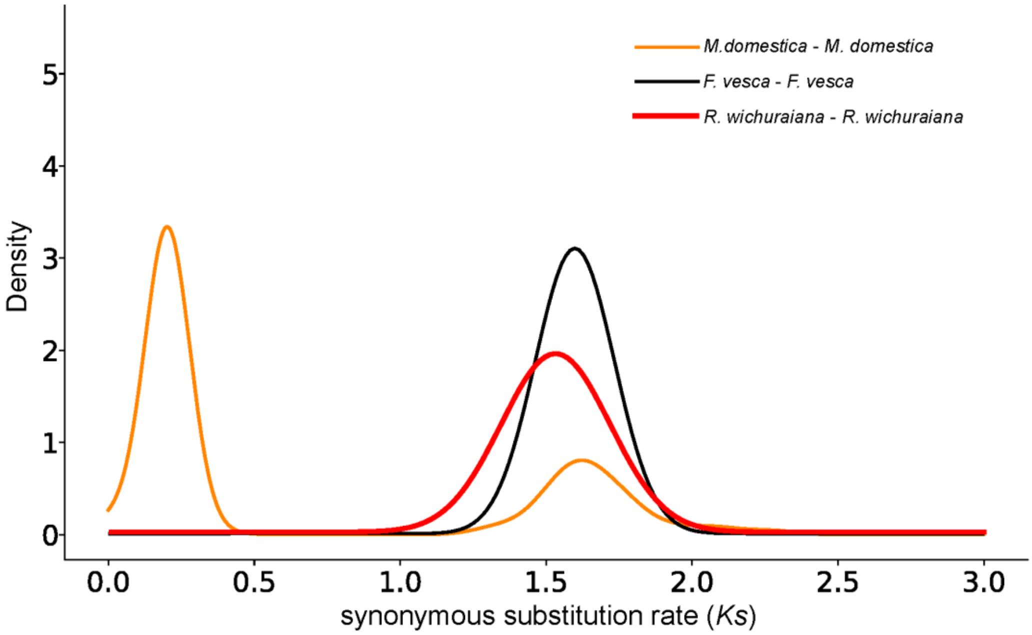

Fig. S15. The density distribution of synonymous substitutions per synonymous site (*Ks*) for syntenic genes in BT, apple, and strawberry.

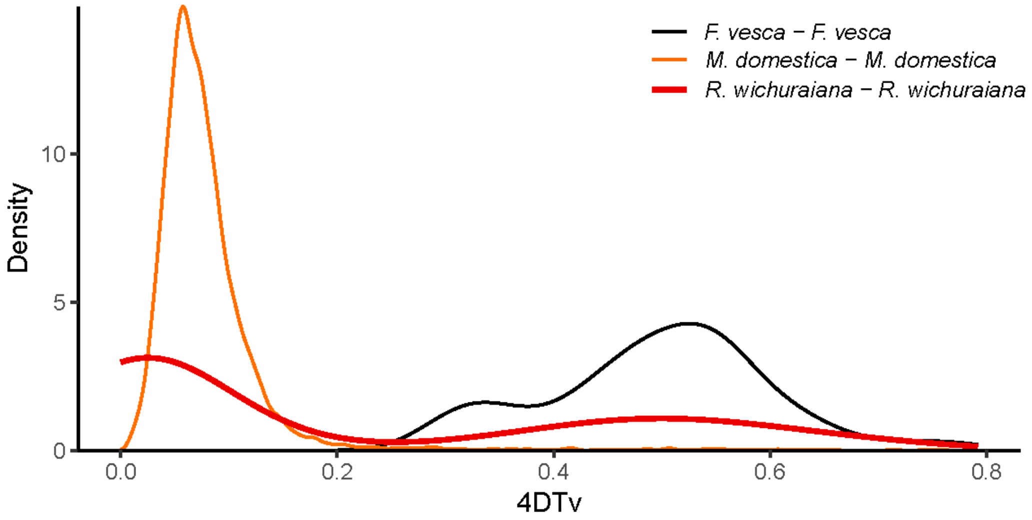

Fig. S16. The distribution of the rate of transversions on fourfold degenerate synonymous sites (4DTv) among paralogs for BT, strawberry, and apple. The ancient WGD featured by BT was shown in a red thick line.

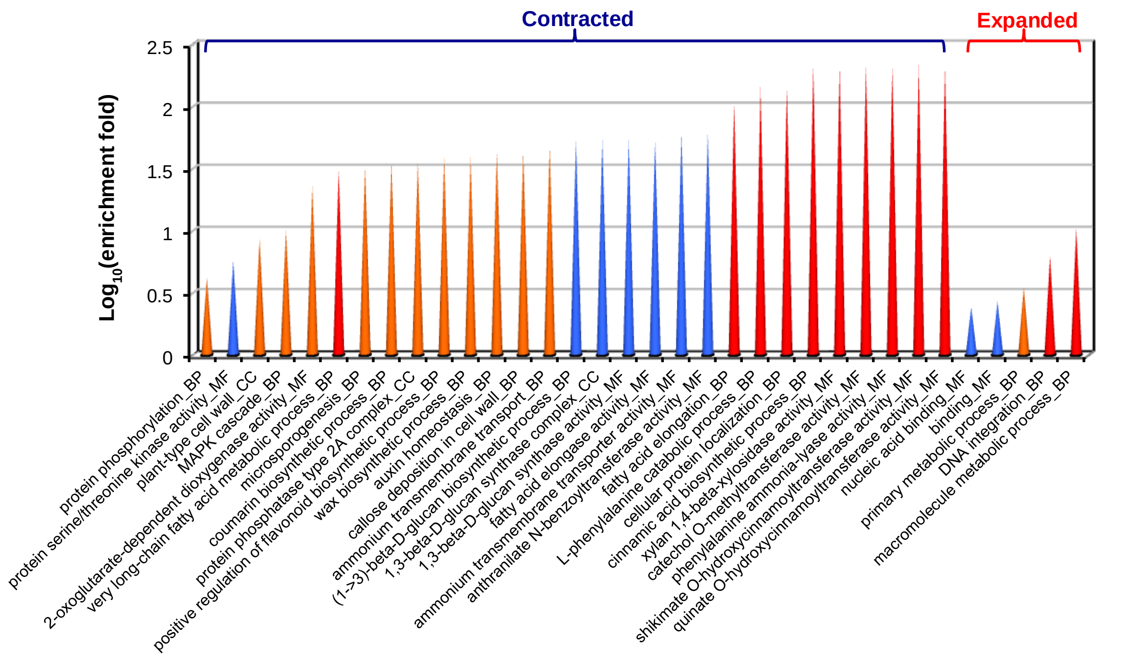

Fig. S17. The enrichment of GO terms for contracted (blue) and expanded (red) gene families in BT (see also Table S20). Cones in orange, blue, and red indicated the significance level of FDR<0.05, 0.05<FDR<0.01, and FDR<0.001, respectively. BP, biological process; CC, cellular components; MF, molecular function.

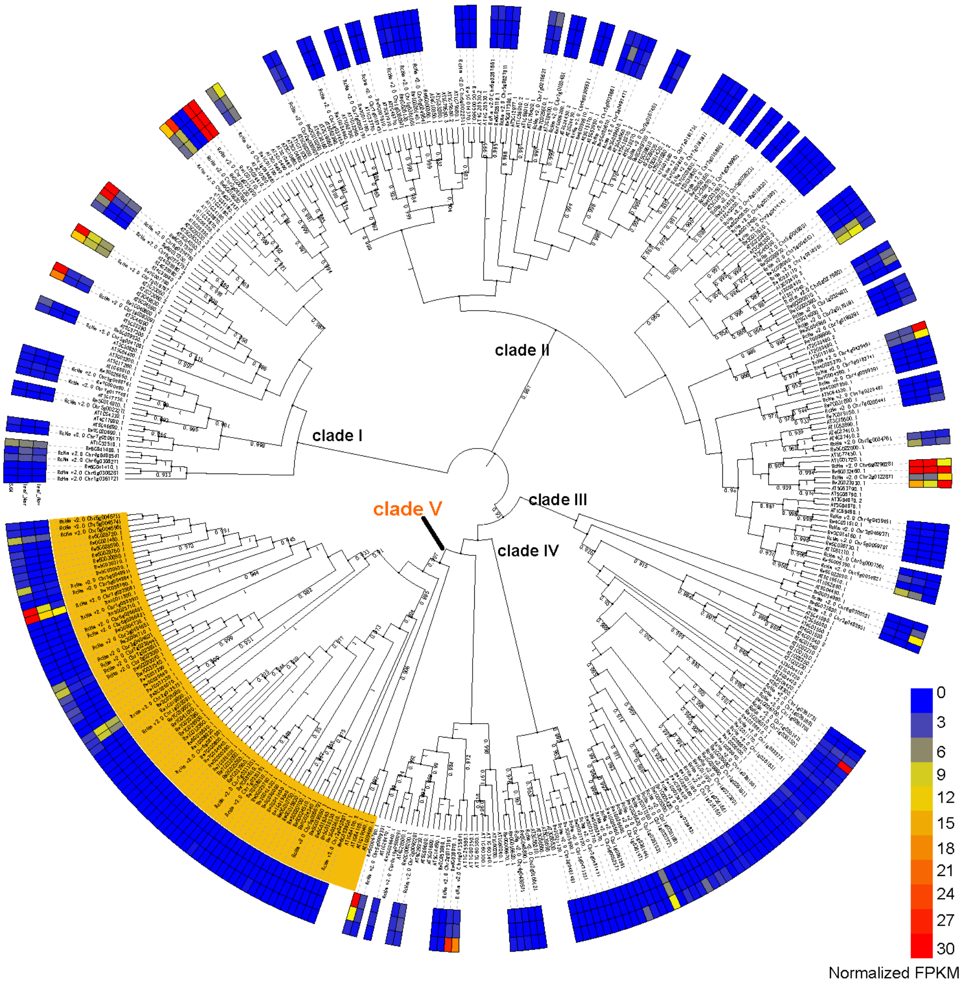

Fig. S18. A lineage specific expansion of *NAC* family genes (clade V, marked in orange) revealed by a phylogenetic reconstruction using Neighboring joining method for genes in BT, haploOB and Arabidopsis. Circles from outside to inside indicated the relative expression levels in FPKM values in shoot apical meristem (SAM), March leaves (Mar) and November leaves (Nov).

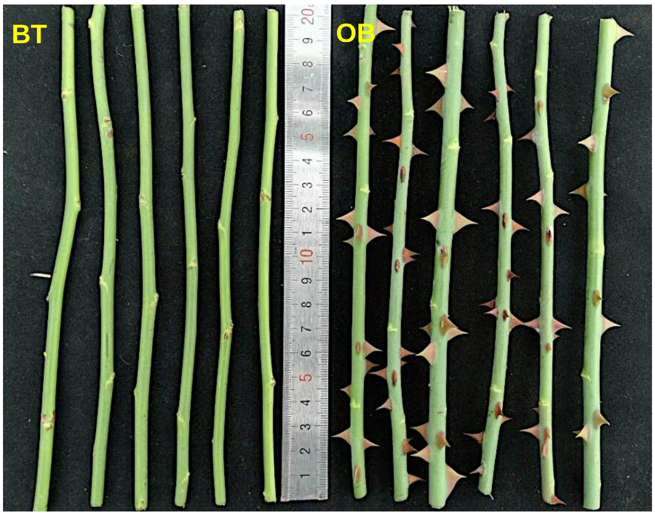

Fig. S19. Prickle distribution on stems of parental lines, BT (left panel) and OB (right), of the mapping population used in this study. Prickle density was arbitrarily defined as the prickles number on 20cm stem of each genotype.

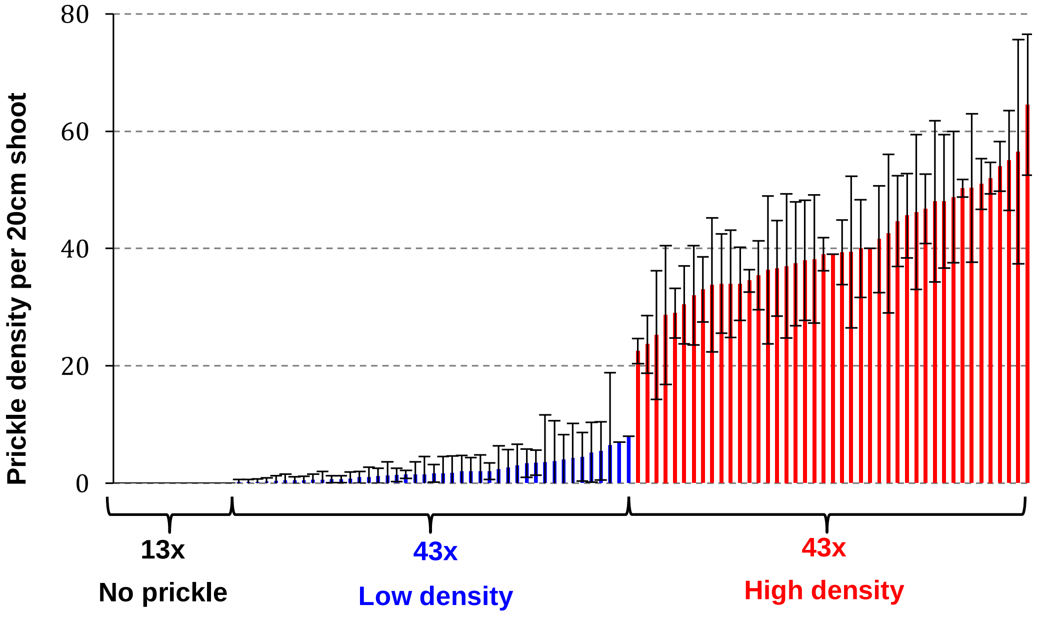

Fig. S20. Prickle density per unit in F1 population between OB x BT in 2014. Numbers under X-axis indicate the plant individuals featuring different levels (no, low density in blue bars, and high density with red colored bars) of prickle density.

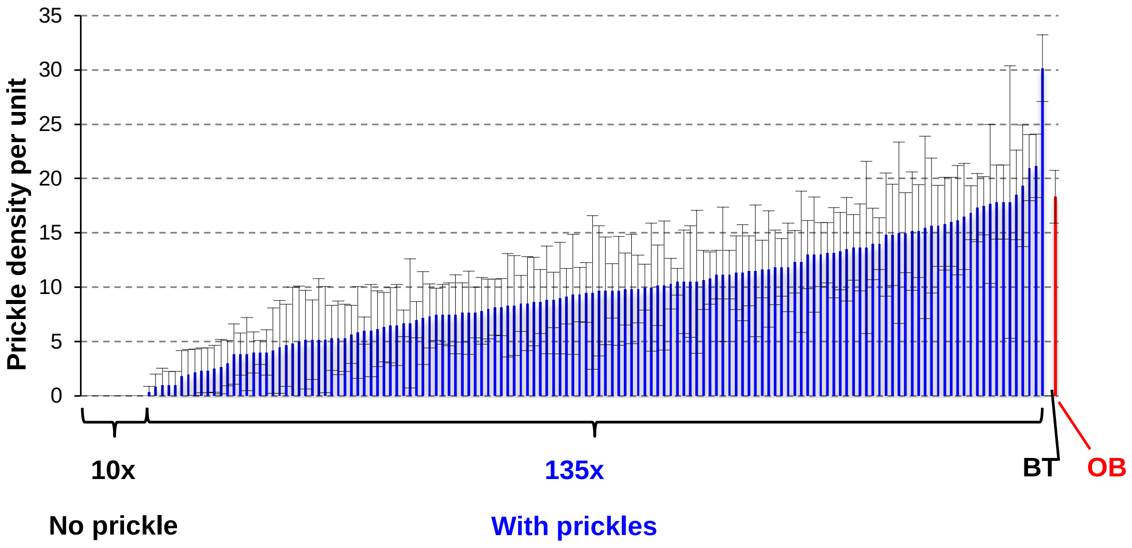

Fig. S21. Prickle density per unit in BC1F1(OB) population. Numbers under X-axis indicate the plant individuals featuring different levels (no, low-high density, and very high density) of prickles. BT shows no prickle while OB (in red bar) features much prickles.

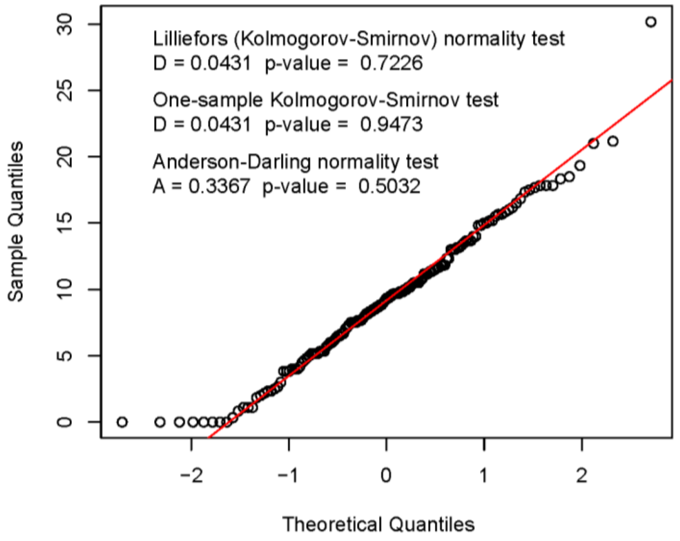

Fig. S22. Statistical tests the normality of data distribution for prickle density per unit in BC1F1(OB) population. Note that all three methods reveal that the prickle density data of BC1F1 population is normally distributed.

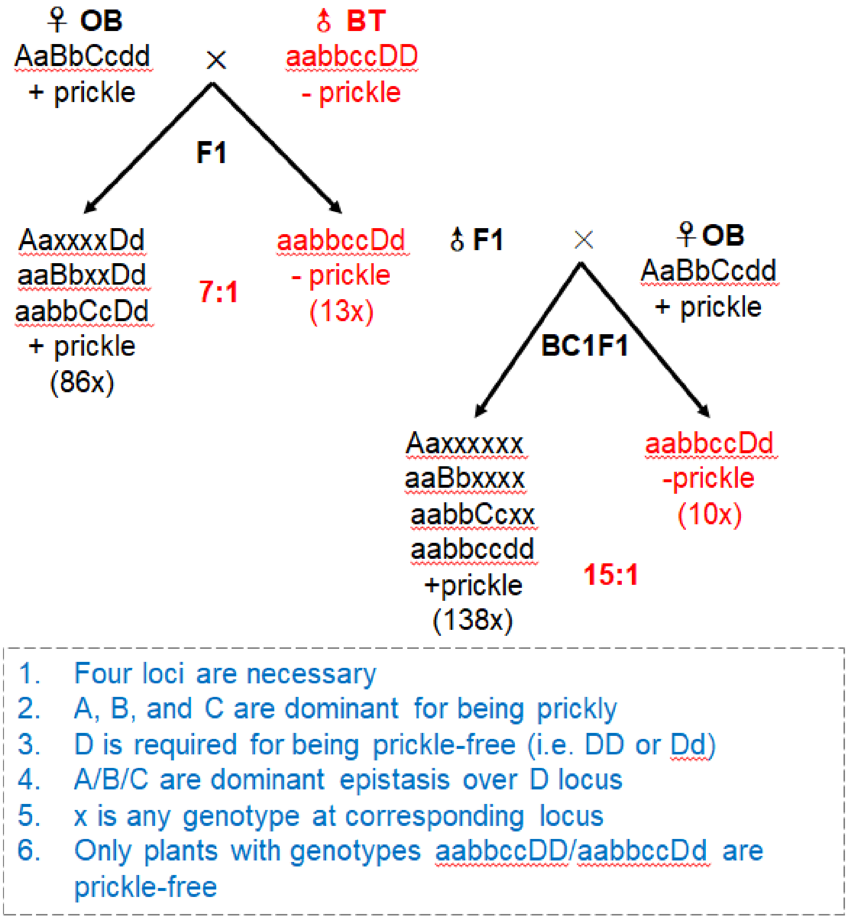

Fig. S23. A hypothetical model for genetic inheritance of prickle density in roses. Four loci were necessary for the regulation of prickle-free in roses.

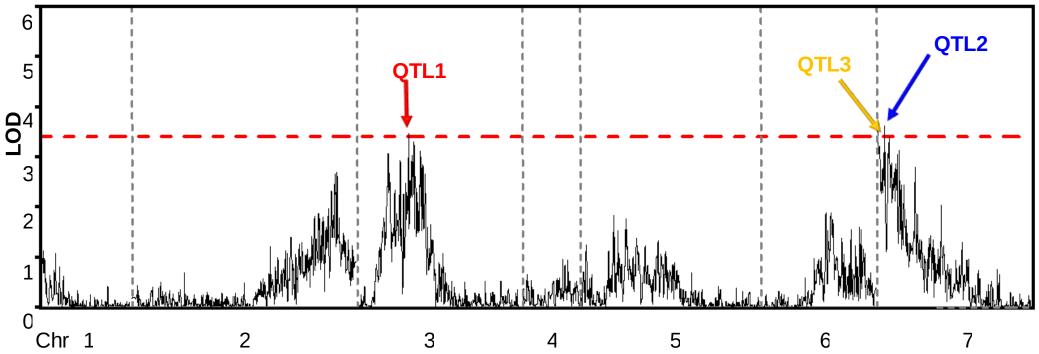

Fig. S24. QTL mapping analysis detected three QTL using interval mapping (IM) method. QTL1 and QTL2 were identical to the identified QTL1 and QTL2 on Chr3 and Chr7, respectively (Fig. 3).

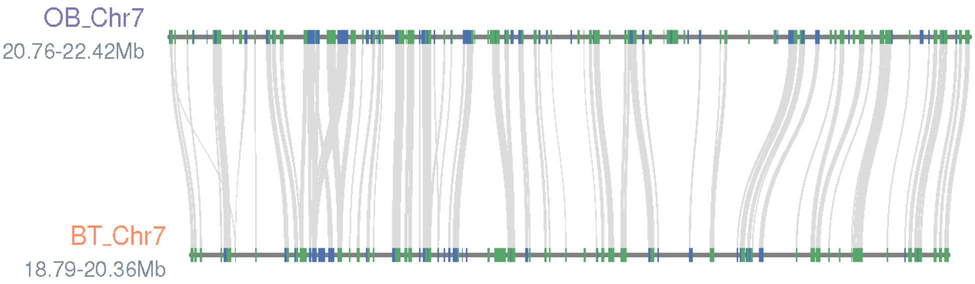

Fig. S25. Gene collinearity for QTL2 between BT and OB.

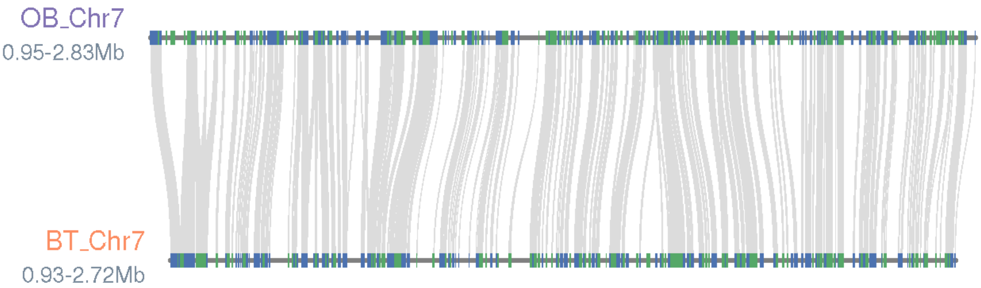

Fig. S26. Gene collinearity for QTL3 detected with IM method.

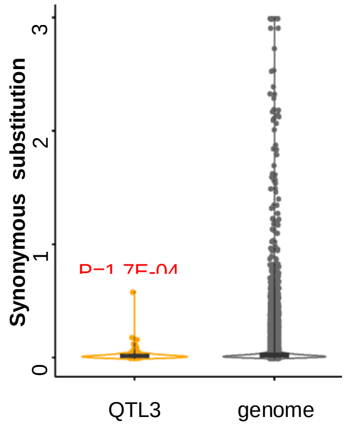

Fig. S27. Comparison of synonymous substitutions (*Ks*) between QTL3 and rest of the genome (*Kruskal-Wallis multiple-comparison test*).

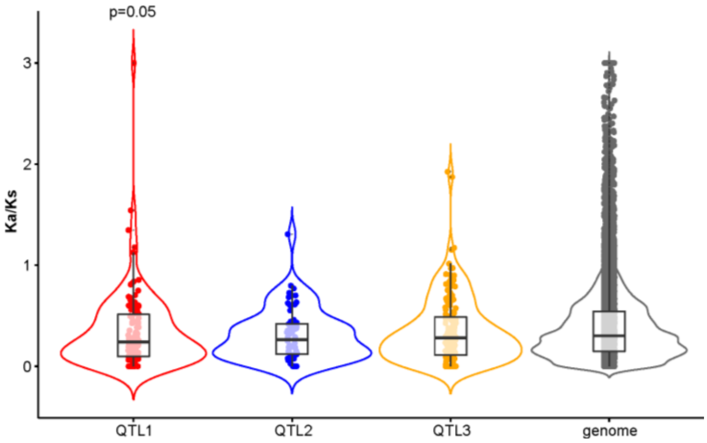

Fig. S28. *Ka/Ks* analysis revealed that genes in QTL1 region were under

purifying selection (*Wilcoxon rank sum test*, p=0.05) comparing to genes in QTL2, QTL3 and rest of the genome.

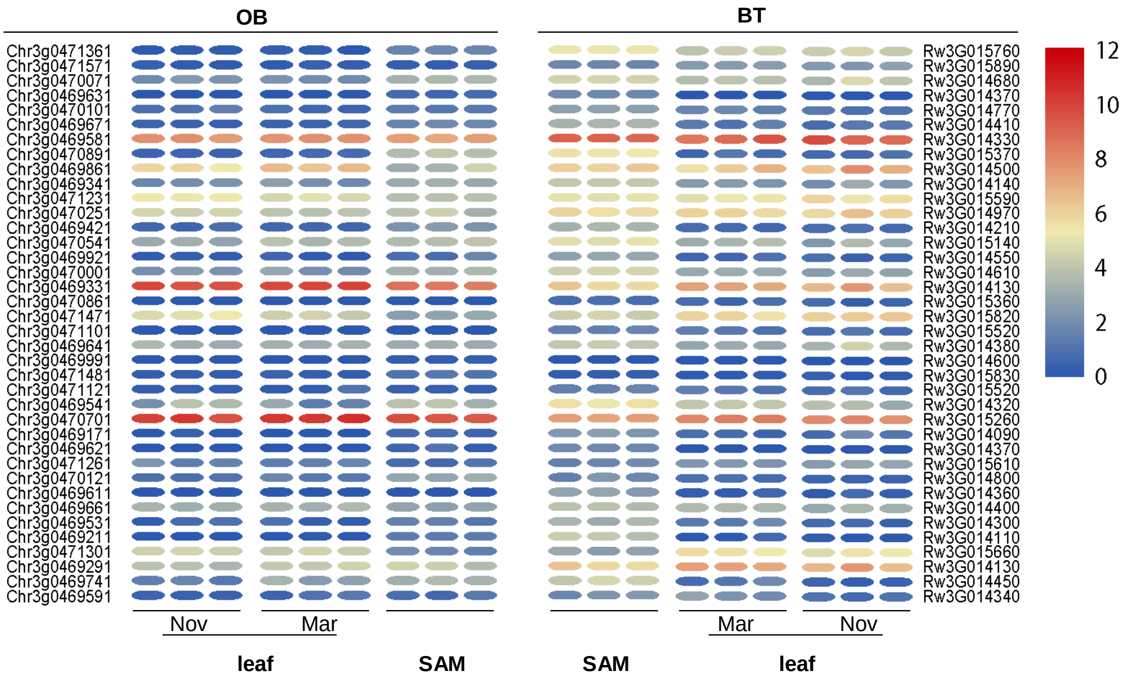

Fig. S29. Heat map showing the expression of 38x genes in QTL1 region that showing differential expression in early young shoots of OB and BT.

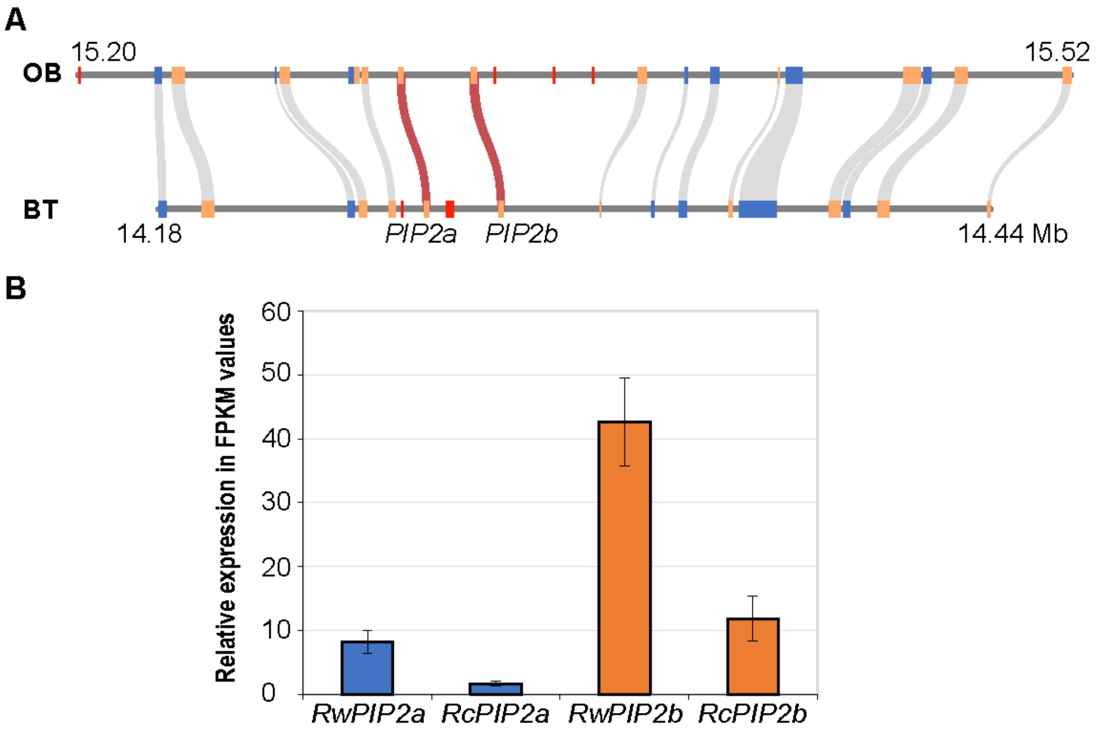

Fig. S30. Rose *PIP2* genes featured a differential expression in shoot apical meristems (SAM). **A**. Genomic structure surrounding *PIP2* genes (connected

in dark red lines) in OB and BT. Genes linked with gray lines marked the syntenic genes (bars in blue and orange) and the position for non-syntenic genes (bars in red). **B**. Relative expression in FPKM values for *PIP2*s in both OB and BT.

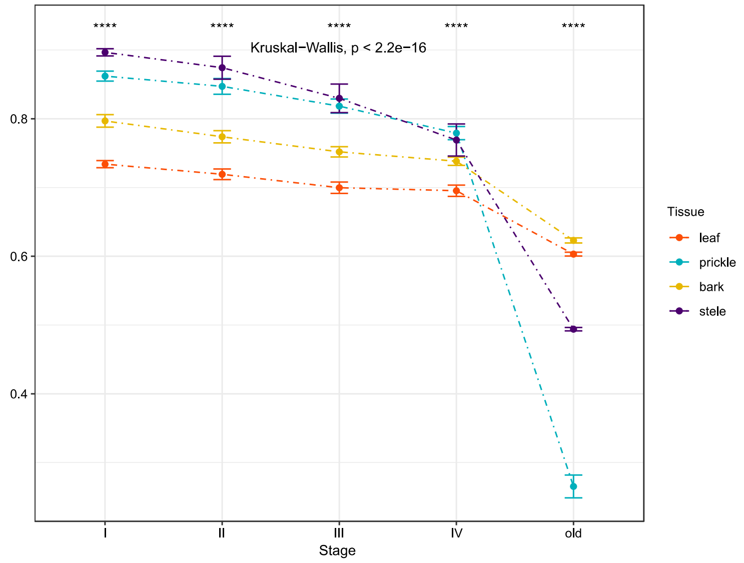

Fig. S31. Variation of relative water contents for different tissues following developmental stages in rose genotype C336 (see Fig. 3h). Bars gave the standard deviation of means. Note that relative water content in prickles (light blue) sharply dropped when shoots aged. Statistical comparison was carried out with *Kruskal-Wallis test* in *R*.

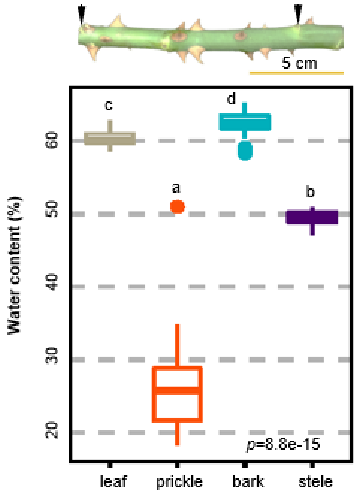

Fig. S32. Relative water content in prickles dropped sharply in old shoots. Letters (a, b, c, and d) above each boxplot indicated the pairwise significances

(p<0.01; non-parametric *Wilcoxon test*) for eight node sections.

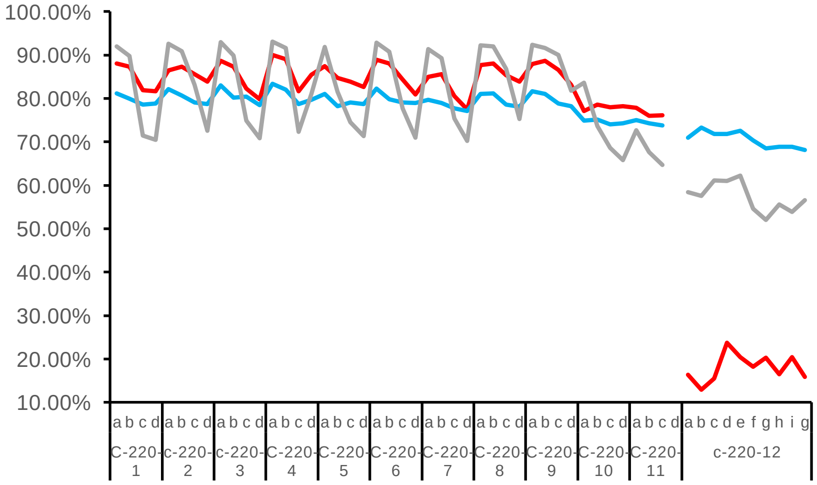

Fig. S33. Relative water content in prickle (red), epidermis (light blue), and stele (gray) of one modern rose genotype (C-220). Four sections (a, b, c, and d, from top to bottom of each shoot) were cut to measure the water content of each tissue. From shoot 1 to 9, young shoots; shoots 10 and 11, relatively old shoots, shoot 12, old shoot produced in previous season. Note that relative water content varied much less in epidermis in all shoots, while it changed significantly in both prickles and steles following developmental stages of shoots (from a to d, and from shoot 1-9 to shoot 10-11, and to shoot 12 (a to g)).

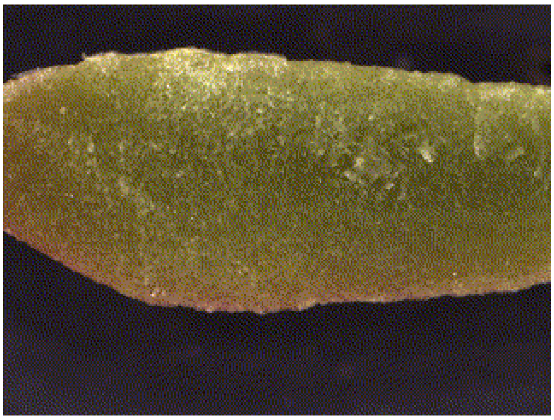

Fig. S34. A detached prickle, which was tweezed lightly by forceps, contained large amount of water (bright region).

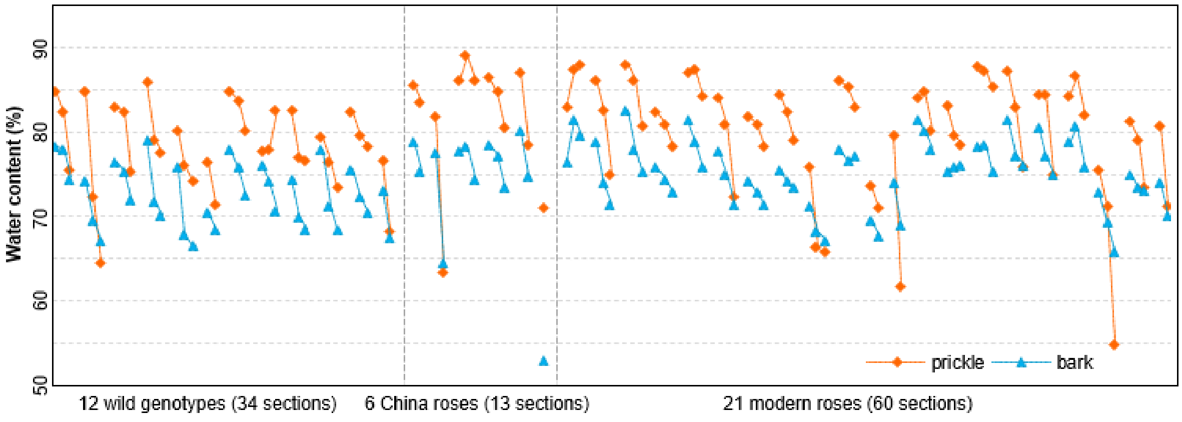

Fig. S35. Prickles (red) generally contained relatively higher water contents than epidermis (blue; p<0.001, *Student's t-test*) in a collection of randomly selected roses (12 wild rose, 6 China rose, and 21 modern cultivated genotypes). Most genotypes had two to three sections measured for one relatively young shoot. Line connection for each genotype indicated the sections from top to bottom on shoot.

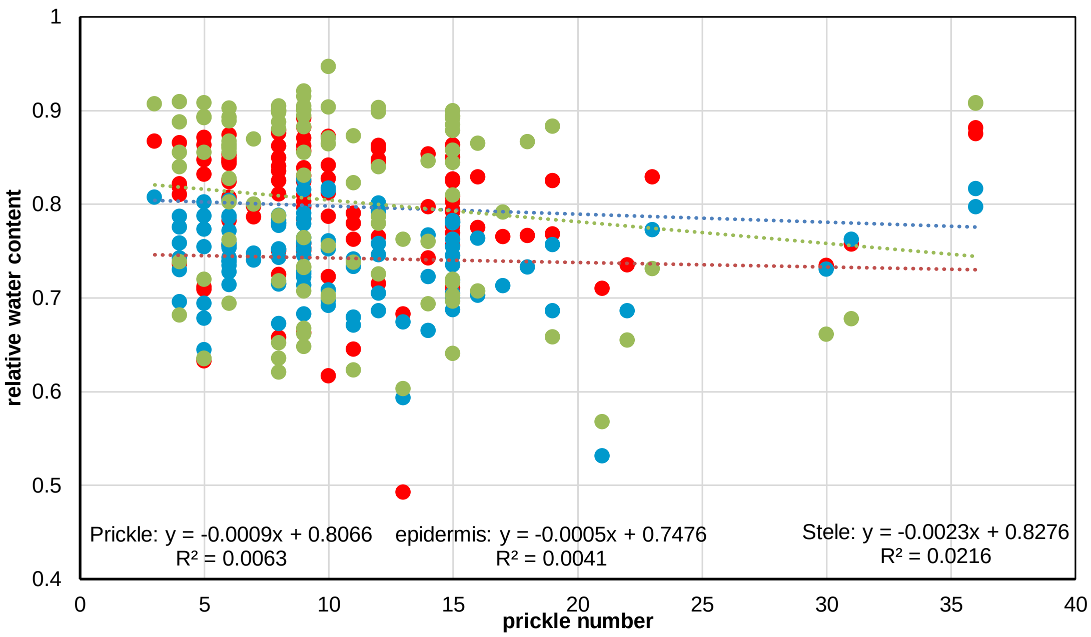

Fig. S36. Relative water content (Y-axis) in prickle (red), epidermis (light blue), and stele (gray) did not vary following prickle number per section (X-axis) of shoot in 41 rose genotypes.

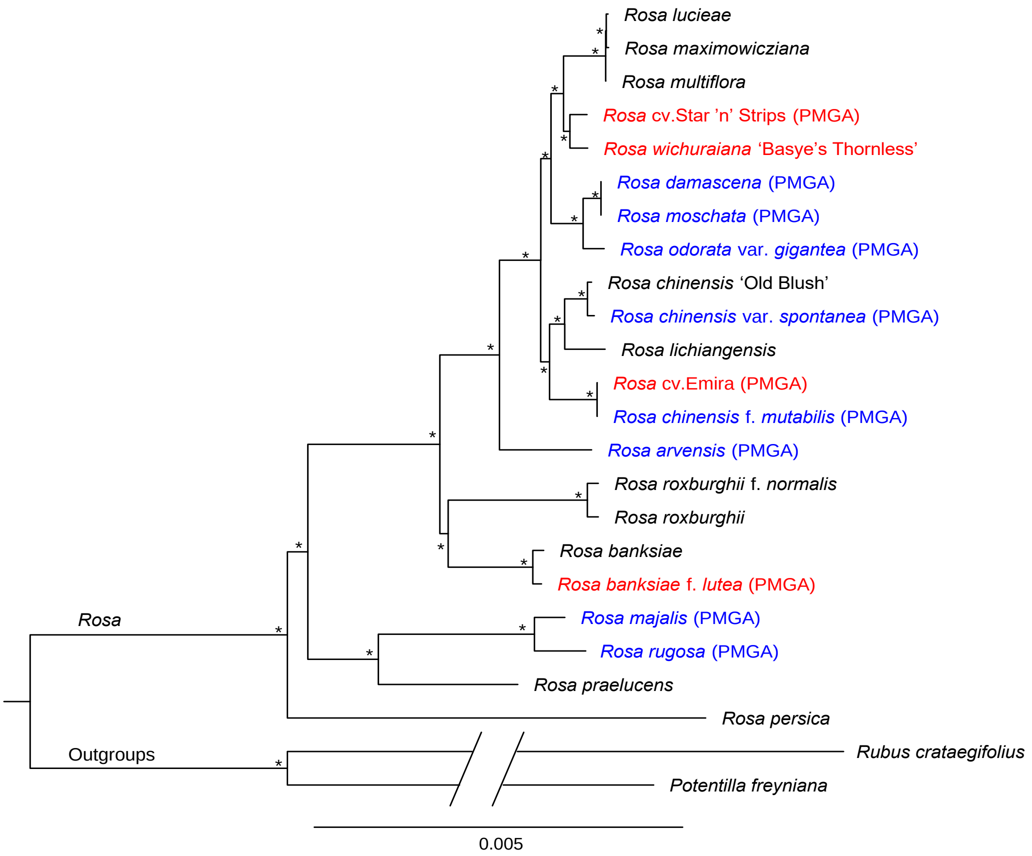

Fig. S37. Phylogenetic analysis of 22 rose genotypes based on chloroplast genome sequences. The ML tree showed relationships used in population genetic analysis of prickly *Rosa* species (in blue) with prickle free *Rosa* species (in red) using *Rubus crataegifolius* and *Potentilla freyniana* as outgroups. Bootstrap supports more than 90% were designated as *. Samples marked with PMGA indicate the chloroplast genome sequences generated in this study.

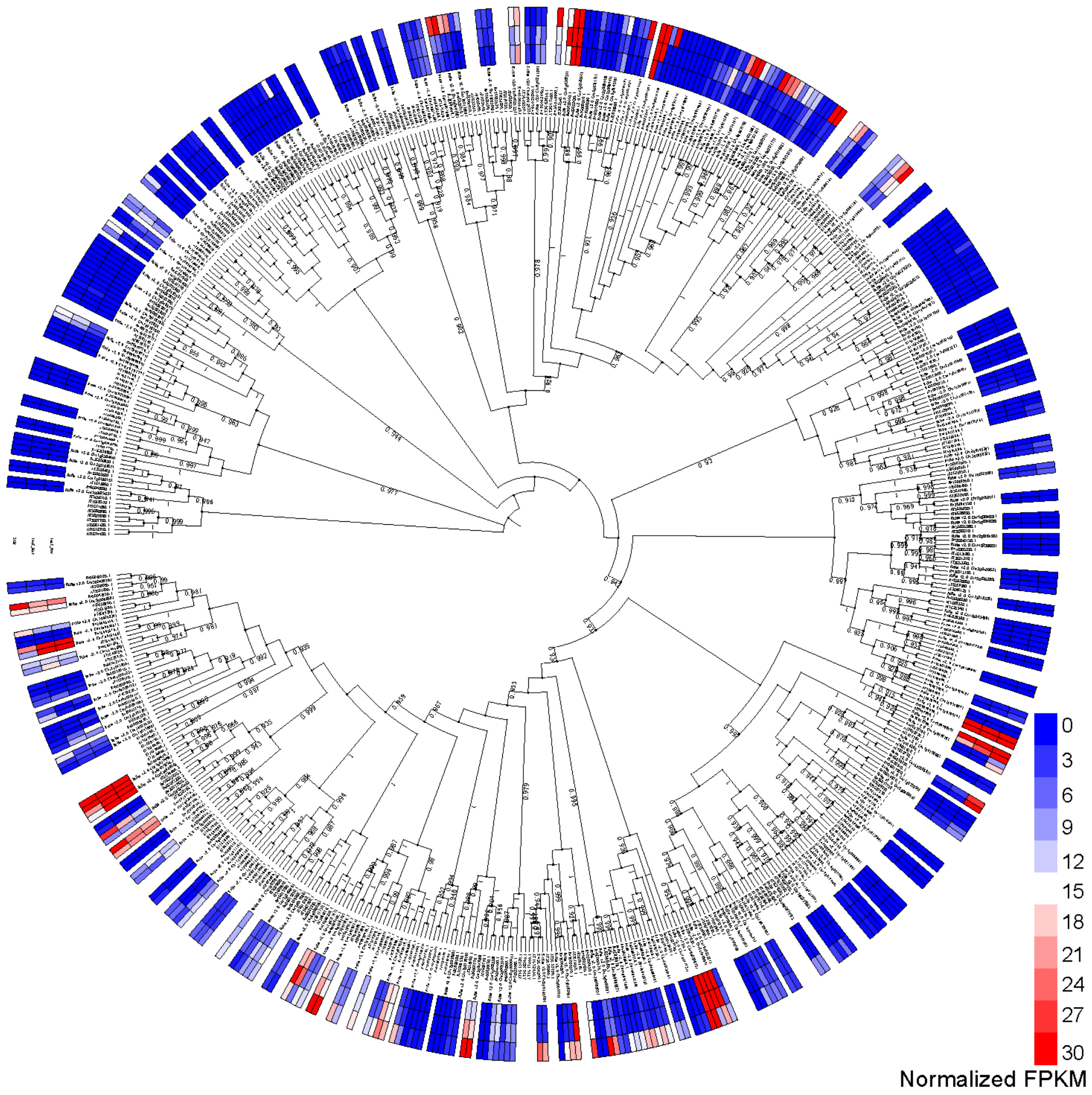

Fig. S38. Phylogenetic analysis of MYB family transcription factors in BT and OB with all MYB TFs from *Arabidopsis thaliana*. Values on branches indicated the bootstrap values for 1000 times simulation. Circles from outside to inside indicated the relative expression levels in FPKM values in shoot apical meristem (SAM), March leaves (Mar) and November leaves (Nov).

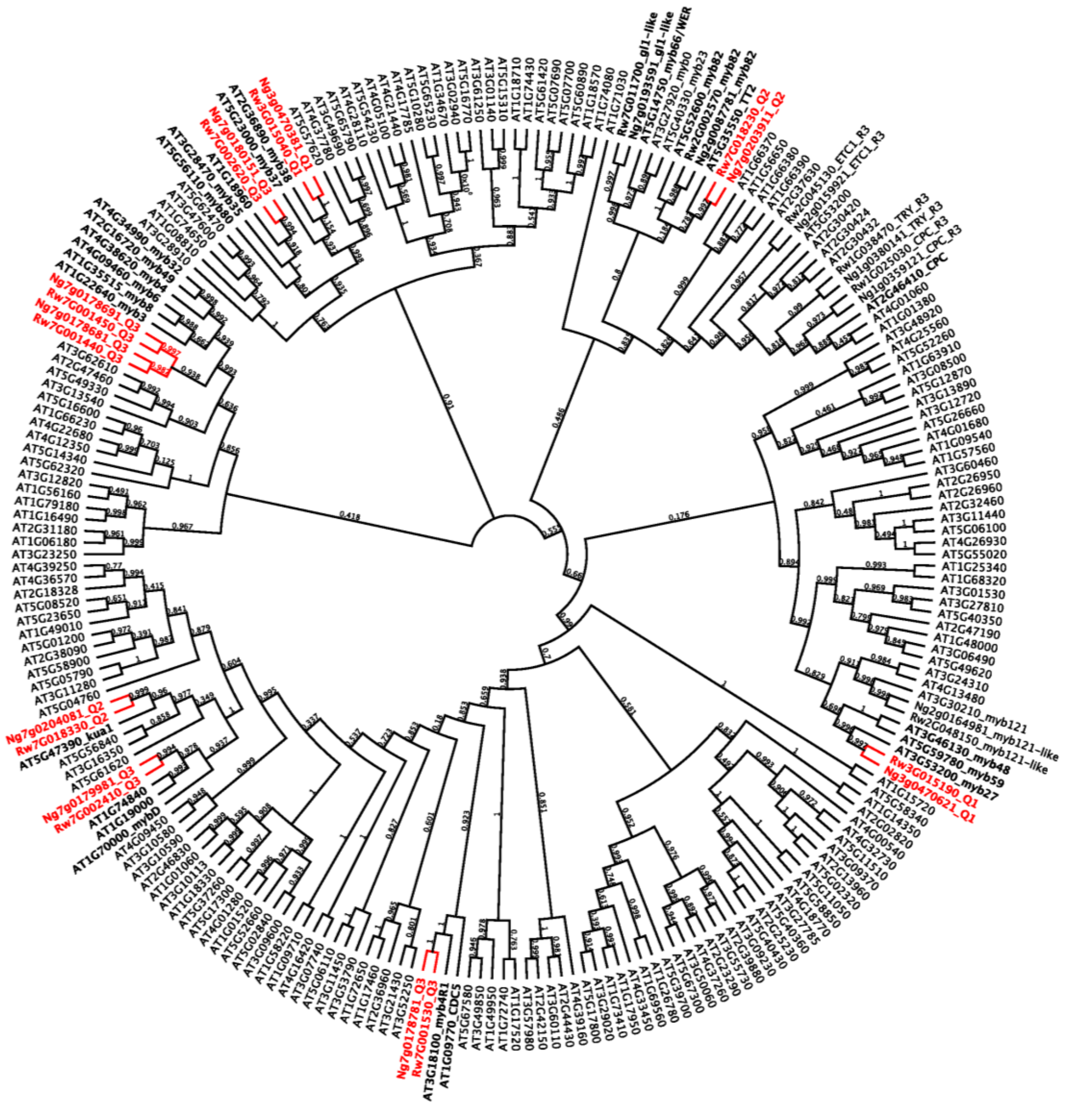

Fig. S39. Phylogenetic analysis of MYB family transcription factors in QTL regions of BT and OB (in red IDs) with all MYB TFs from *Arabidopsis thaliana*. Values on branches indicated the bootstrap values for 1000 times simulation.

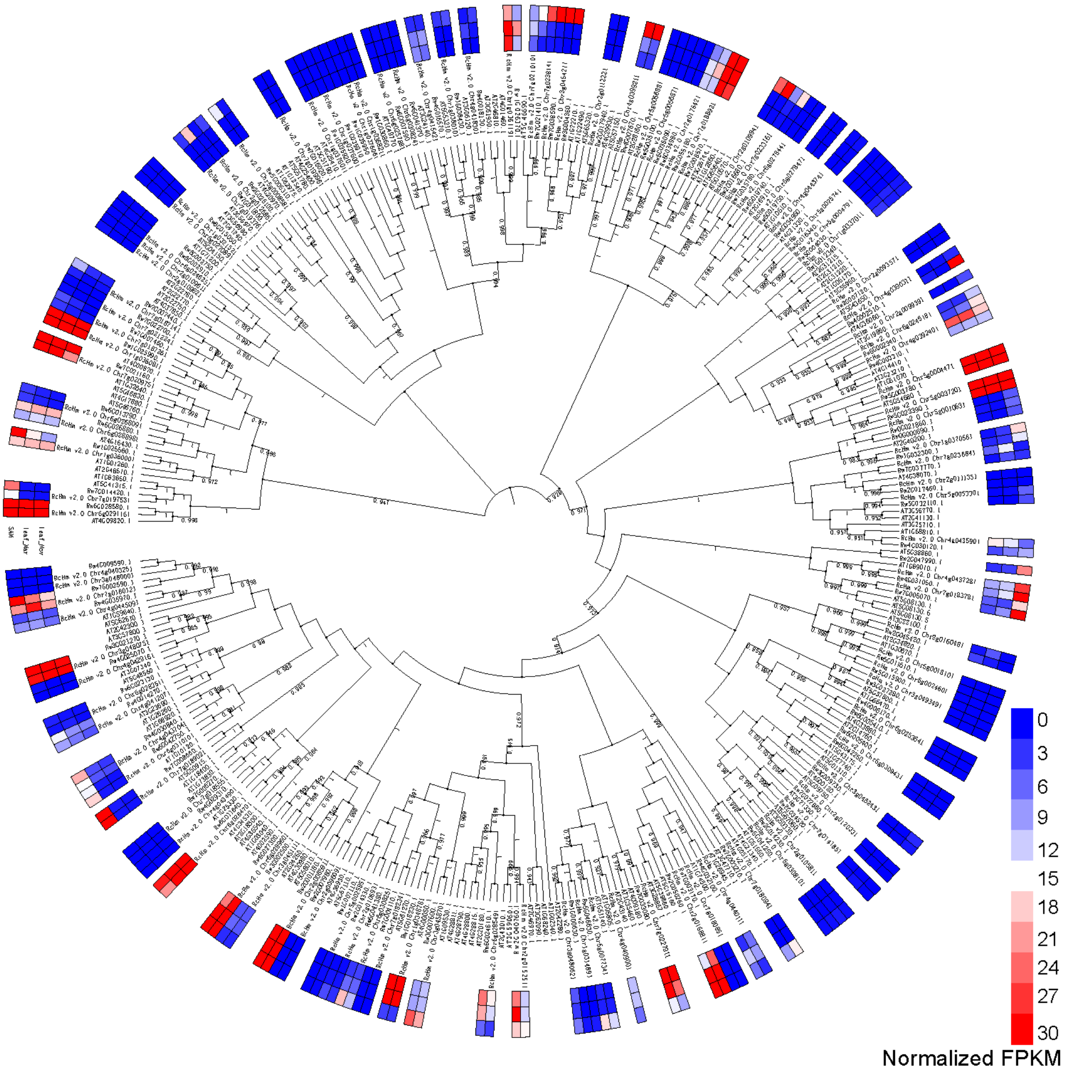

Fig. S40. Phylogenetic analysis of bHLH family transcription factors at genome-wide level in *Arabidopsis thaliana*, BT and OB. Values on branches indicated the bootstrap values. Circles from outside to inside indicated the relative expression levels in FPKM values in shoot apical meristem (SAM), March leaves (Mar) and November leaves (Nov).

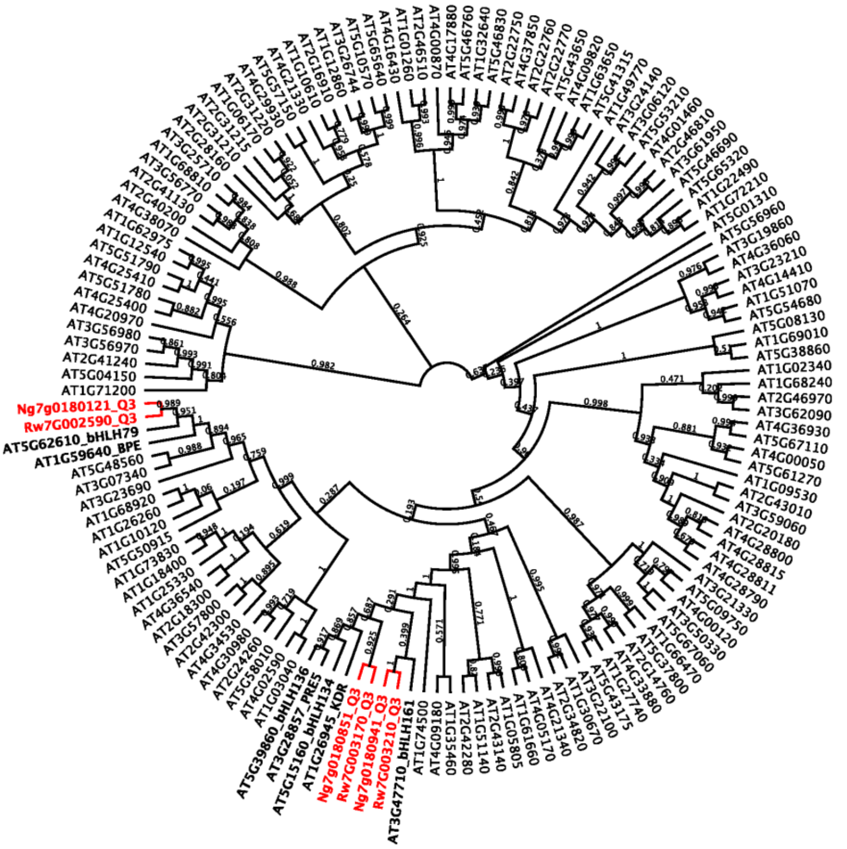

Fig. S41. Phylogenetic analysis of bHLH family transcription factors in QTL regions of BT and OB with all genes from *Arabidopsis thaliana*. IDs in red marked the genes located in QTL regions. Values on branches indicated the bootstrap values.

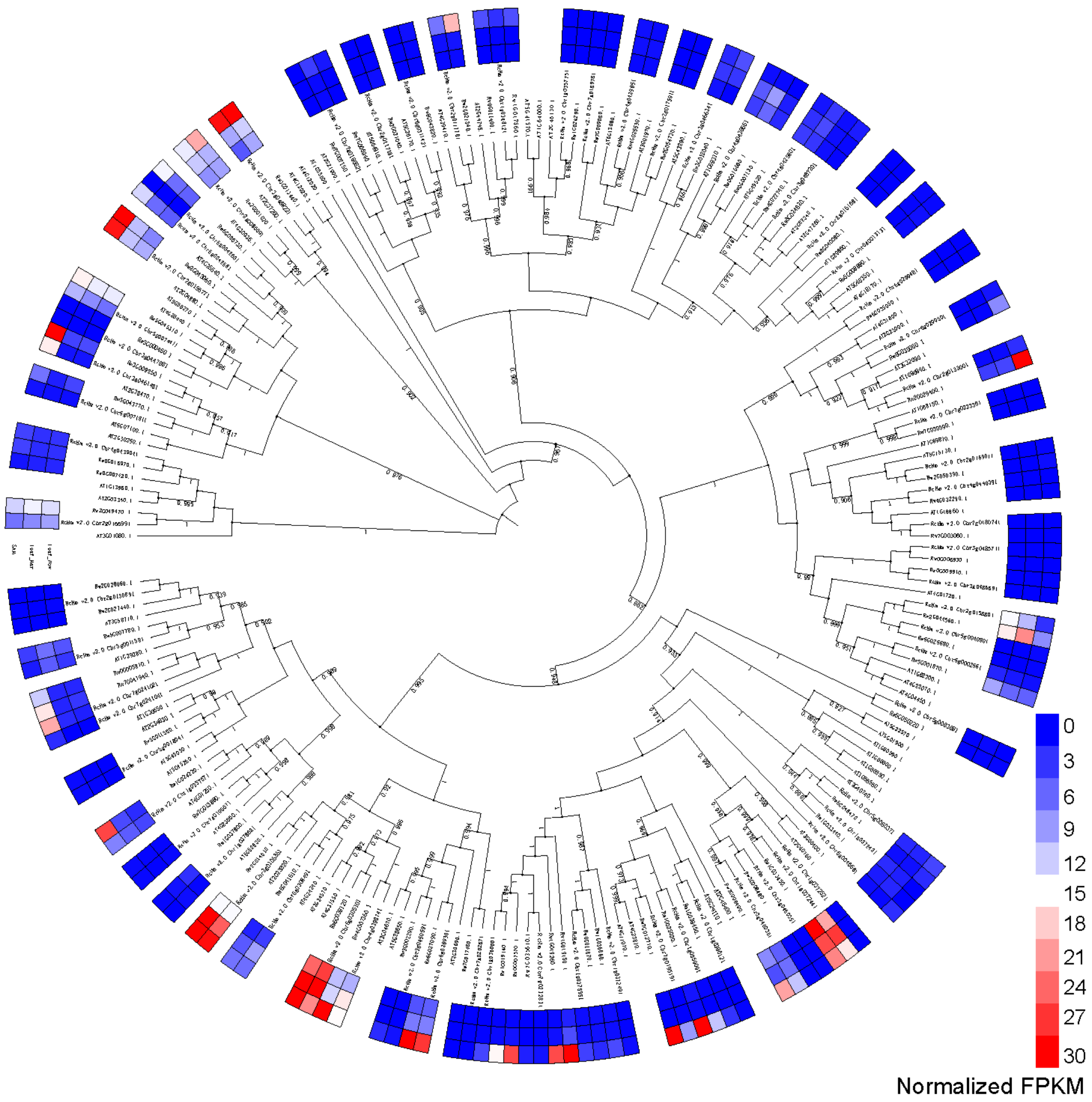

Fig. S42. Phylogenetic analysis of WRKY family transcription factors in *Arabidopsis thaliana*, BT and OB. Values on branches indicated the bootstrap values. Circles from outside to inside indicated the relative expression levels in FPKM values in shoot apical meristem (SAM), March leaves (Mar) and November leaves (Nov).

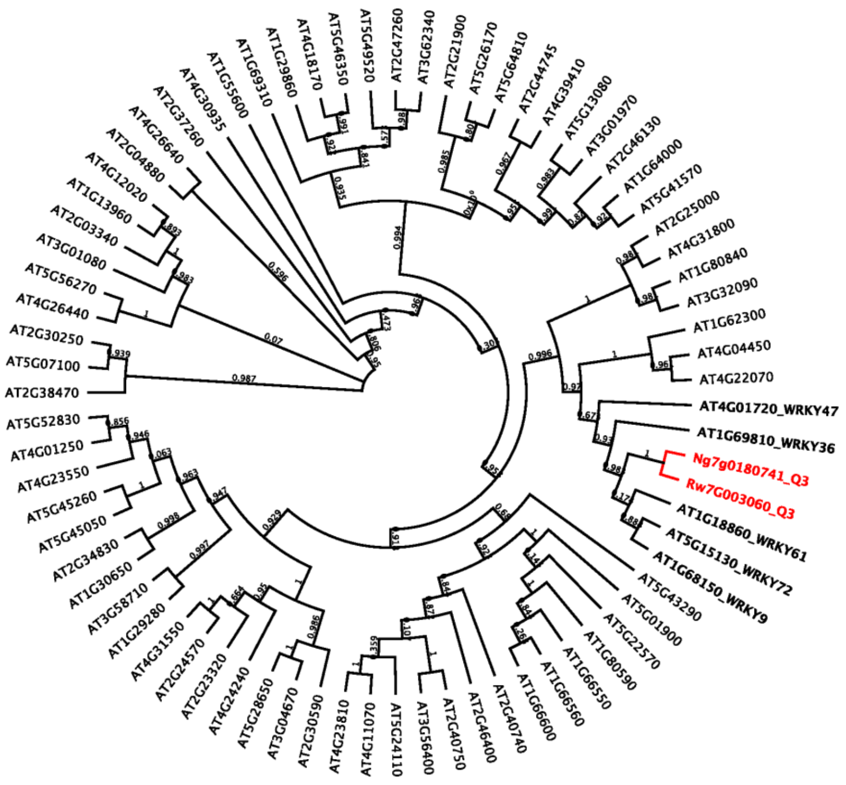

Fig. S43. Phylogenetic analysis of WRKY family transcription factors in *Arabidopsis thaliana*, BT and OB. IDs in red marked the genes located in QTL regions. Values on branches indicated the bootstrap values.

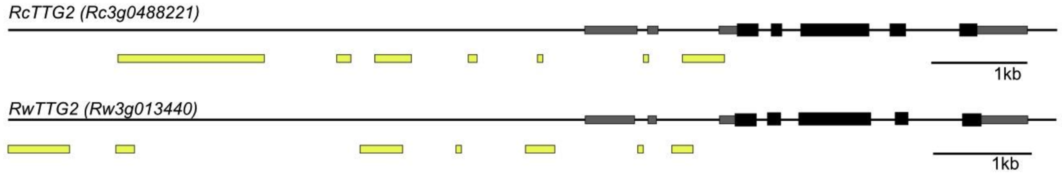

Fig. S44. *TTG2* gene structure and LTR location (yellow boxes) of BT and OB.
