# Supplementary material for: Rose without prickle: genomic insights linked to moisture adaptation
Source: Natl Sci Rev. 2021 May 22;8(12):nwab092. doi: 10.1093/nsr/nwab092 (PMC8694671; doi:10.1093/nsr/nwab092)
Supplement: nwab092_Supplemental_Files [file nwab092_supplemental_files.zip › SupplTablesv3NSRrev1.pdf]

A genomic link in China roses: and they all lived prickly but water deficient ever after?

Mi-Cai Zhong [1,4], Xiao-Dong Jiang [1,4], Guo-Qian Yang [2], Wei-Hua Cui [1,4], Zhi-Quan Suo [1,4], Wei-Jia Wang [3], Yi-Bo Sun [1,4], Dan Wang [1,4], Xin-Chao Cheng [5], Xu-Ming Li [5], Xue Dong [1,2], Kai-Xue Tang [3,*], De-Zhu Li [1,2,*], Jin-Yong Hu [1,*]

Table S1-S36.

Table S1. Summary of sequence data for *R. wichuraiana* 'Basyes' Thornless' (BT).

Molecules Type	Seq Methods	Library size (bp)	Data size (Gb)	Reads N50 (bp)
DNA	HiSeq NextSeq 500 (Paired-end)	260, 360	140.34	149
DNA	PacBio Sequel	20,000	48.77	12,833
DNA (Hi-C)	Novaseq-6000	350	19.48	150
RNA	HiSeq 2000	150	42.2	150
**DNA**	ONT	20,000	47.58	21,531

Table S2. Summary of 17K-mer analysis of BT genome.

Property	Value
K-mer number	576,274,867
k-mer depth(x)	229
Average read length (bp)	147.37
Total reads number	917,344,232
Heterozygosity (%)	1.026
Genome size (bp)	525,536,719

Table S3. Summary of the genome assembly for BT based on Illumina reads with *SOAPdenovo* pipeline.

Property	Scaffold
Max sequence length	331,360
Total sequence number	119,191
N20	17,291
N20 Number	3,392
N50	7,890
N50 Number	15,585
N90	1,868
N90 Number	61,195
N number	264,906,790
N rate	0.579843
Total sequence length	456,859,475
GC content	0.405825
Sequences greater than 1kb	79,997

Table S4. Genome assembly summary for BT.

Methods	Contig number	Contig length (bp)	Contig N50 (bp)	Contig N90 (bp)	Contig max (bp)	Plat form
CANU	5,895	863,587,669	286,345	54,897	9,021,729	PB
Falcon	9,441	869,725,664	273,058	31,331	2,886,427	PB
WTBDG	4,551	502,891,200	271,752	40,872	2,378,540	PB
CANU+Falcon	3,261	675,805,885	488,583	96,537	5,354,204	PB
CANU+Falcon+ SSPCE-longread+PBjelly	2,715	689,839,017	650,671	114,032	5,875,838	PB
CANU+WTBDG+Quickmerge	1,847	526,658,451	932,234	165,547	8,250,845	PB
NECAT	755	833,569,586	2,823,823	723,581	10,313,637	ONT
Canu+TGS-GapCloser+NextPolish	1554	530,039,516	1,637,359	200,795	10,008,337	PB+ONT+Illumina

Table S5. Statistics of Hi-C interaction data for BT.

Data Type	Number	Proportion size (%)
Total read pairs	66,260,843	100
Mapped Reads	120,305,752	90.78
Unique Mapped Read Pairs	34,639,986	52.28
Valid Interaction Pairs	28,069,999	81.03
Dangling End Pairs	2,146,546	6.2
Re-ligation Pairs	507,645	1.47
Self-cycle Pairs	2,152,456	6.21
Dumped Pairs	1,763,340	5.09

Table S6. Statistics of Hi-C assembly for BT chromosomes.

Group	Chr number	Sequence length prior to manual correction (bp/contig number)	Sequence length after manual correction (bp)
Lachesis Group0	Chr5	94,476,199/277	87,438,908
Lachesis Group1	Chr2	86,780,347/221	82,908,596
Lachesis Group2	Chr1	74,430,481/281	68,419,088
Lachesis Group3	Chr6	72,289,640/225	68,242,664
Lachesis Group4	Chr7	72,029,547/220	66,527,531
Lachesis Group5	Chr4	70,691,057/245	64,309,030
Lachesis Group6	Chr3	50,624,202/149	46,038,772
Total Sequences Clustered		521,321,473 (99%)	
Total Sequences Ordered and Oriented		481,766,853 (92.41%)/857(52.97%)	483,884,589

Table S7. Assembly statistics of *R. wichuraiana* 'Basyes' Thornless' (BT).

assembly features	statistic
estimated genome size (K-mer=17)	525,536,719 bp
number of contigs	1,554
contig N50	1,637,359 bp
longest contig length	10,008,337 bp
assembled genome size	530,067,488 bp
number of pseudo-chromosomes	7
total assembly on chromosomes	483,884,589 bp (91.29%)

contig number not ordered/oriented	1,093
contig length not ordered/oriented	46,182,899 bp (8.71%)

Table S8. BUSCO (v4.1.0; in total 2326) evaluation of the BT genome.

	Complete BUSCOs	Complete and single-copy BUSCOs	Complete and duplicated BUSCOs	Fragmented BUSCOs	Missing BUSCOs
**Count**	2,184	1956	228	69	73
**Proportion**	93.9%	84.1%	9.8%	3.0%	3.1%

Table S9. Confirmation of the BT genome assembly by K5 and OB x BT genetic maps.

	K5 map		OB x BT map	
**Chromosome ID**	**R^2 value**	**p-value**	**R^2 value**	**p-value**
chr1	0.9464398	0	0.8988454	2.732595e-51
chr2	0.9817721	0	0.9457076	1.305217e-185
chr3	0.9437266	0	0.8949968	4.106149e-99
chr4	0.944322	0	-0.6353362	7.885129e-12
chr5	0.980934	0	0.9500027	3.45689e-153
chr6	0.9500137	0	0.972675	9.668612e-110
chr7	0.9826604	0	0.9163256	1.396654e-94

Table S10. Mapping proportion of the Illumina reads and PacBio reads to the assembled BT genome.

Methods	Total reads number	Mapped reads	Mapped proportion (%)
Illumina	465,656,935	457,109,201	98.16
PacBio	2,301,273	2,218,225	96.39

Table S11. Equal or even higher quality of the new BT genome in comparison to previously published haploOBs. * For BUSCO data, C, completed; F, fragmented; M, missing. # For anchored and assembly completeness ratios, length in Mb. Numbers before and after / indicated the estimated genome size based on genome survey (*Kmer*=17), and C-value measurement. LAI, LTR-assembly-Index.

genome	Genome type	Contig N50	BUSCO*	LAI	Anchored ratio #	Assembly completeness #	Gene collinearity
haploOB1 (Raymond et al. 2018)	haploid	24 Mb	96.5% C/ 1.5% F/ 2% M	15.03	503.9/515.6 97.7%	515.6/(526/560) 92.1%~98.0%	58.03% (haploOB1 vs. haploOB2)
haploOB2 (Hibrand Saint-Oyant et al. 2018)	haploid	3.4 Mb	92.5% C/ 4.1% F/ 3.4% M	20.3	466/512 91%	512/(532.7/568) 90.1%~96.1%	58.86% (haploOB1 vs. BT)
BT (this study)	diploid	1.64 Mb	93.9% C/ 3% F/ 3.1% M	20.04	481.7/521 92.4%	521/(526/532) 97.9%~99.1%	58.12% (haploOB2 vs. BT)

Table S12. Repeat sequences in the BT genome.

Type	Number	Length (bp)	Proportion (%)
ClassI/DIRS	17,992	12,908,716	2.44
ClassI/LINE	56,115	23,018,305	4.34
ClassI/LTR	2,134	2,162,587	0.41
ClassI/LTR/Copia	217,834	149,954,347	28.29
ClassI/LTR/Gypsy	111,907	86,882,194	16.39
ClassI/PLE|LARD	134,823	55,209,811	10.42
ClassI/SINE	6,125	1,468,377	0.28
ClassI/TRIM	2,696	1,223,440	0.23

ClassI/Unknown	434	86,835	0.02
ClassII/Crypton	144	90,363	0.02
ClassII/Helitron	25,761	10,661,541	2.01
ClassII/MITE	6,895	1,523,818	0.29
ClassII/Maverick	33	18,815	0
ClassII/TIR	65,961	30,625,423	5.78
ClassII/Unknown	11,205	3,072,530	0.58
PotentialHostGene	11,141	3,534,695	0.67
SSR	828	225,813	0.04
Unknown	93,913	25,552,303	4.82
Unknown/Helitron|LARD	19	7,804	0
Total	765,960	345,577,859	65.19

Table S13. *Gypsy* and *Copia* proportion in genomes of Rosaceae and other sequenced plants.

**Species**	***Gypsy* proportion (%)**	***Gypsy* length (Mb)**	***Copia* proportion (%)**	***Copia* length (Mb)**	***Gypsy* /*Copia* ratio**	**Reference**
Rosa wichuraiana	16.39	86.88	28.29	149.95	0.58	This study
Rosa chinensis	15.7	81.4	12.63	65.5	1.24	Hibrand Saint-Oyant et al. 2018
Rosa chinensis	12.91	/	9.97	/	1.29	Raymond et al. 2018
Fragaria vesca	6.39	12.9	5.33	10.76	1.2	Shulaev et al. 2011
Rosa multiflora	3.7	27.73	6.6	49.16	0.56	Nakamura et al. 2018
Malus domestica	25.2	187.1 Mb	5.5	40.6Mb	4.58	Velasco et al. 2010
Prunus avium	/	8	/	8.4	*0.95*	Shirasawa et al. 2017
Prunus persica	9.97	/	8.6	/	*1.16*	Verde et al. 2013
Pyrus communis	14.1	84.6Mb	7.1	42.8 Mb	*1.99*	Chagne et al. 2014
Rubus occidentalis	11.6		10.6		*1.09*	Vanburen et al. 2016
Ziziphus jujube	17.32	75.82Mb	12.64	55.32Mb	*1.37*	Liu et al. 2014
Vitis vinifera	14	70.7 Mb	4.8	24.1 Mb	*2.92*	
Soybean	25.3	281.9 Mb	10.7	119.1 Mb	*2.36*	
Poplar	4.9	23.9 Mb	1.6	7.8 Mb	*3.06*	
Arabidopsis thaliana	5.2	7.0 Mb	1.4	1.9 Mb	*3.71*	
Rice	12	50.5 Mb	2.5	10.4 Mb	*4.8*	
Brachypodium	16.1	43.5 Mb	4.9	13.2 Mb	*3.29*	
Sorghum bicolor	19	140.6 Mb	5.2	38.3 Mb	*3.65*	
Maize	46.4	948 Mb	23.7	484 Mb	*1.96*	
Pineapple	17.05	87.48Mb	4.46	22.90 Mb	*3.82*	Chen et al. 2019
Coix	36.1	/	28.4	/	*1.27*	Liu et al. 2019
Black pipper	25.37	193.13 Mb	9.15	69.68 Mb	*2.77*	Hu et al. 2019
Liriodendron	40.45	704.67 Mb	13.08	227.86	*3.09*	Chen et al. 2019
Stout camphor tree	10.4	/	6.09	/	*1.71*	Chaw et al. 2019
Zostera marina	/	39.11	/	23.63	*1.66*	Olsen et al. 2016
Sugarcane	26.04	/	14.19	/	*1.84*	Zhang et al. 2018
Bracteatus pineapple	17.05	87.48	4.46	22.9	*3.82*	Chen et al. 2019
Musa balbisiana	12.88	63.49	28.04	138.17	*0.46*	Wang et al. 2019
Chenopodium quinoa	28.46	394.37	8.22	113.93	*3.46*	Jarvis et al. 2017
Date palms	6.35	49.04	8.78	67.8	*0.72*	Hazzouri et al. 2019
Macleaya cordata	/	51.3	/	37.3	*1.38*	Liu et al. 2017
Camellia sinensis	45.85	1421.48	8.24	255.55	*5.56*	Wei et al. 2018

Antirrhinum majus L	13.97	71.31	15.58	79.49	*0.90*	Li et al. 2019
Hot pepper	51.12	1566.21	6.01	184.2	*8.51*	Kim et al. 2014
Macleaya cordata	13.59	51.33	9.87	37.28	*1.38*	Liu et al. 2017
Hardy Rubber	16.7	197.28	13..20	156.2	*1.26*	Wuyun et al. 2018
E ulmoides	16.7	197.28	13.22	156.2	*1.26*	
Arachis duranensis	18.14	170.53	3.17	29.83	*5.72*	Bertioli et al. 2016
Arachis ipaensis	1.86	23.34	2.98	37.43	*0.62*	Bertioli et al. 2016
Octoploid strawberry	11.94	96.17	2.69	21.64	*4.44*	Edger et al. 2019
Paper mulberry	20.33	78.63	10.64	41.15	*1.91*	Peng et al. 2019
Salix brachista	9.44	32.06	9.15	31.07	*1.03*	Chen et al. 2019
Apple	25.41	167.42	15.86	104.48	*1.60*	Zhang et al. 2019
Ricinus communis	11.45	38.6	4.77	16.08	*2.40*	Chan et al. 2010
Silver birch	8.54	32.13	2.29	8.61	*3.73*	Salojarvi et al. 2017
Brassica oleracea	/	14.68	/	20.77	*0.71*	Belser et al. 2018
Brassica rapa	/	11.48	/	8.86	*1.30*	Belser et al. 2018
Gossypium barbadense	41.4	946.42	7.17	163.86	*5.77*	Hu et al. 2019
Citrus dementina	12.01	36.19	7.88	23.76	*1.52*	Wu et al. 2014

Table S14. Statistics of gene model prediction for BT genome.

Method	Software	Species	Gene number
Ab initio	Genscan	-	26867
	Augustus	-	26000
	GlimmerHMM	-	34542
	GeneID	-	45986
	SNAP	-	21339
Homology-based	GeMoMa	*Arabidopsis thaliana*	25552
		Oryza sativa	27157
		Fragaria vesca	33806
		Malus domestica	35056
		Pyrus bretschneideri	29890
RNAseq	PASA	-	10872
	TransDecoder	-	56188
	GeneMarkS-T	-	33014
Integration	EVM	-	32674

Table S15. Summary of protein coding gene models in BT genome.

**Gene number**	32,674
Total gene length (bp)	107,221,785
Average gene length (bp)	3,281.56
Total exon length (bp)	41,962,143
Average exon length (bp)	250.83
Total intron length (bp)	65,259,642
Average intron length (bp)	390.09

Table S16. Annotation summary for protein coding genes of BT genome.

Database	Annotated number	Percentage (%)
GO	15,357	47.00%
KEGG	11,496	35.18%
KOG	17,997	55.08%

TrEMBL	31,459	96.28%
nr	31,771	97.24%
All annotated	31,800	97.33%

Table S17. Transcription factors (TFs) and transcription regulators (TRs) in BT and haploOB genomes. na, not available.

TF/TR	family	BT	haploOB.Raymond	haploOB.Hibrand
TR	MED6	1	2	2
TR	RB	1	1	1
TR	MBF1	2	2	2
TR	SOH1	2	1	1
TR	Pseudo ARR-B	3	5	5
TR	Rcd1-like	3	3	4
TR	Coactivator p15	4	3	3
TR	SWI/SNF-SWI3	4	4	4
TR	LUG	5	5	5
TR	MED7	5	5	5
TR	IWS1	7	8	8
TR	TAZ	7	6	7
TR	HMG	9	9	9
TR	ARID	11	11	11
TR	Jumonji	17	25	23
TR	SWI/SNF-BAF60b	21	18	17
TR	AUX/IAA	22	23	23
TR	TRAF	22	18	18
TR	GNAT	28	36	32
TR	SET	39	46	45
TR	PHD	40	39	36
TR	SNF2	41	39	39
TR	mTERF	42	60	50
TR	Others TRs	65	57	60
**TR sum**	**24**	**401**	**426**	**410**
TF	BSD	1	1	na
TF	HB-PHD	1	2	2
TF	LFY	1	1	1
TF	NOZZLE	1	1	na
TF	S1Fa-like	1	1	1
TF	SAP	1	1	1
TF	ULT	1	1	1
TF	DBP	2	2	2
TF	HRT	2	2	2
TF	NF-X1	2	2	2
TF	VOZ	2	2	2
TF	BBR-BPC	3	3	3
TF	C2C2-LSD	3	3	3

TF	EIL	3	4	3
TF	CAMTA	4	4	4
TF	CSD	4	5	4
TF	NF-YA	4	7	7
TF	Whirly	4	2	3
TF	Alfin-like	5	6	6
TF	CPP	5	5	5
TF	DBB	5	4	4
TF	GARP-ARR-B	5	5	5
TF	AP2/ERF-RAV	6	5	5
TF	DDT	6	5	4
TF	LIM	6	6	6
TF	SRS	6	6	6
TF	BES1	7	7	9
TF	C2C2-YABBY	7	6	6
TF	GeBP	7	5	4
TF	HB-BELL	8	10	10
TF	C2C2-CO-like	9	8	8
TF	GRF	9	10	10
TF	HB-KNOX	9	9	9
TF	HB-other	9	14	9
TF	NF-YC	9	9	8
TF	RWP-RK	9	8	8
TF	TUB	9	8	10
TF	zf-HD	9	9	10
TF	E2F-DP	10	14	13
TF	PLATZ	11	10	10
TF	OFP	12	13	10
TF	Tify	12	12	11
TF	NF-YB	14	13	13
TF	AP2/ERF-AP2	16	15	15
TF	SBP	17	17	16
TF	B3-ARF	18	17	17
TF	C2C2-GATA	18	17	19
TF	HSF	20	18	21
TF	HB-WOX	21	na	na
TF	TCP	21	18	17
TF	C2C2-Dof	24	24	21
TF	HB-HD-ZIP	28	28	27
TF	Trihelix	30	26	26
TF	GARP-G2-like	38	31	32
TF	MADS-M-type	40	48	44
TF	LOB	41	38	38
TF	MADS-MIKC	41	34	33
TF	bZIP	52	49	51

TF	C3H	61	66	63
TF	GRAS	61	59	56
TF	MYB-related	62	57	56
TF	WRKY	73	68	67
TF	B3	90	98	94
TF	FAR1	91	52	35
TF	C2H2	99	103	93
TF	bHLH	106	107	104
TF	AP2/ERF-ERF	108	121	111
TF	MYB	137	132	131
TF	NAC	146	114	111
**TF sum**	**69**	**1703**	**1608**	**1538**

Table S18. Annotation summary of non-coding and pseudo-genes in BT genome.

Type	Count/Family
miRNA	71<br>20 families: miR156, 2; miR159, 4; miR160, 3; miR162, 2, miR164, 1; miR166, 2; miR167, 3; miR168, 1; miR169, 17; miR171, 10; miR172, 4; miR390, 2; miR393, 4; miR395, 5; miR396, 1; miR397, 2; miR398, 2; miR399, 4; miR408, 1; miR828, 1
tRNA	557<br>24 families
rRNA	78<br>4 families: 7 (18S), 19 (28S), 1 (5.8S), 51 (5S)
snRNA	83
snoRNA	274
Pseudo-gene	4254<br>(mean length 2928.7bp, counting for 12,490,896bp or 2.58% of BT genome)

Table S19. SNPs and indels counts upon mapping the Illumina reads to BT genome.

	SNP	Indels
Counts	2,303,621	506,113
Transition	1,452,119	/
Transversion	858,324	/
Ti/Tv	1.69	/
Heterozygous SNPs	2,296,379	/
Homozygous SNPs	7,102	/
Heterozygous proportion (%)	99.69%	/

Table S20. SNP and indel variants distribution among BT chromosomes.

SNP count	SNPs	Indels
Chr1	292,962	64,261
Chr2	330,460	75,914
Chr3	227,414	49,667
Chr4	326,573	69,516
Chr5	377,736	79,280
Chr6	321,340	69,031
Chr7	319,555	70,218

Other contigs	107,581	28,226

Table S21. SNPs and indels mutational effects among BT chromosomes.

mutational effect type	SNP count	SNP Percent	Indel Count	Indel Percent
3_prime_UTR_truncation	/	/	1	0%
3_prime_UTR_variant	18,711	0.49%	6,129	0.67%
5_prime_UTR_premature_start_codon_gain_variant	1,570	0.04%	/	/
5_prime_UTR_truncation	/	/	1	0%
5_prime_UTR_variant	10,063	0.26%	5,391	0.59%
conservative_inframe_deletion	/	/	780	0.09%
conservative_inframe_insertion	/	/	1,353	0.15%
disruptive_inframe_deletion	/	/	532	0.06%
disruptive_inframe_insertion	/	/	716	0.08%
downstream_gene_variant	732,471	19.15%	196,081	21.45%
exon_loss_variant	/	/	4	0%
frameshift_variant	/	/	6,624	0.73%
initiator_codon_variant	23	0.00%	/	/
intergenic_region	1,895,202	49.55%	405,672	44.38%
intron_variant	244,322	6.39%	79,223	8.67%
non_coding_transcript_variant	/	/	104	0.01%
missense_variant	77,906	2.04%	/	/
splice_acceptor_variant	421	0.01%	246	0.03%
splice_donor_variant	248	0.01%	391	0.04%
splice_region_variant	8,715	0.23%	3,470	0.38%
start_lost	98	0.00%	118	0.01%
stop_gained	2,964	0.08%	221	0.02%
stop_lost	2,144	0.06%	181	0.02%
stop_retained_variant	610	0.02%	/	/
synonymous_variant	52,308	1.37%	/	/
upstream_gene_variant	777,332	20.32%	206,886	22.63%

Table S22. Synteny blocks between BT and Raymond's haploOB tested via McScanX. "+" or "-" indicated the block matching direction.

BT_start	BT_end	Gene_num	NG_start	NG_end	Gene_num	direction
Rw1G000020	Rw1G000350	34	Chr1g0313141	Chr1g0313741	44	+
Rw1G000370	Rw1G003750	339	Chr1g0314081	Chr1g0319831	423	+
Rw1G003510	Rw1G004800	130	Chr1g0320081	Chr1g0323411	200	+
Rw1G004610	Rw1G005470	87	Chr1g0323761	Chr1g0324931	98	+
Rw1G005450	Rw1G006240	80	Chr1g0325221	Chr1g0326651	101	+
Rw1G006260	Rw1G007420	117	Chr1g0326921	Chr1g0328841	141	+
Rw1G007440	Rw1G010190	276	Chr1g0329211	Chr1g0332571	283	+
Rw1G010240	Rw1G010700	47	Chr1g0332511	Chr1g0333131	51	-
Rw1G010830	Rw1G011810	99	Chr1g0333181	Chr1g0335271	137	+
Rw1G011820	Rw1G012830	102	Chr1g0336311	Chr1g0337851	122	+
Rw1G012820	Rw1G013580	77	Chr1g0339001	Chr1g0340101	85	-
Rw1G013300	Rw1G015050	176	Chr1g0340151	Chr1g0343091	218	-
Rw1G015050	Rw1G021310	627	Chr1g0343211	Chr1g0352631	732	+
Rw1G021320	Rw1G022890	158	Chr1g0353001	Chr1g0355461	190	+
Rw1G022880	Rw1G023300	43	Chr1g0355681	Chr1g0356161	42	+
Rw1G023110	Rw1G031110	801	Chr1g0356431	Chr1g0368491	940	+

Rw1G031140	Rw1G034020	289	Chr1g0368911	Chr1g0373241	337	+
Rw1G034050	Rw1G034940	90	Chr1g0373261	Chr1g0374521	103	-
Rw1G034980	Rw1G039710	474	Chr1g0374521	Chr1g0381921	562	+
Rw1G039720	Rw1G041560	185	Chr1g0381841	Chr1g0384701	220	-
Rw2G000010	Rw2G003540	354	Chr2g0084221	Chr2g0088741	386	+
Rw2G003570	Rw2G003940	38	Chr2g0089181	Chr2g0089571	38	-
Rw2G003980	Rw2G004290	32	Chr2g0089641	Chr2g0090141	38	-
Rw2G004790	Rw2G005250	47	Chr2g0090861	Chr2g0091371	47	-
Rw2G005270	Rw2G005790	53	Chr2g0091381	Chr2g0092131	60	-
Rw2G005800	Rw2G006350	56	Chr2g0092141	Chr2g0092811	60	-
Rw2G006360	Rw2G007080	73	Chr2g0092861	Chr2g0093541	69	-
Rw2G007150	Rw2G009490	235	Chr2g0094881	Chr2g0098601	288	+
Rw2G009510	Rw2G016510	701	Chr2g0098851	Chr2g0109081	808	+
Rw2G016580	Rw2G021830	526	Chr2g0109721	Chr2g0118331	633	+
Rw2G021820	Rw2G026160	435	Chr2g0118591	Chr2g0126921	571	+
Rw2G026180	Rw2G026610	44	Chr2g0127091	Chr2g0128001	60	-
Rw2G026680	Rw2G027420	75	Chr2g0128091	Chr2g0129301	89	+
Rw2G027440	Rw2G028050	62	Chr2g0129301	Chr2g0130951	93	-
Rw2G028060	Rw2G034320	627	Chr2g0130891	Chr2g0141171	759	+
Rw2G034340	Rw2G049910	1558	Chr2g0141481	Chr2g0167781	1925	+
Rw2G049920	Rw2G052190	228	Chr2g0168251	Chr2g0171711	268	+
Rw2G052340	Rw2G055480	315	Chr2g0172171	Chr2g0176781	368	+
Rw3G000020	Rw3G007170	716	Chr3g0447341	Chr3g0457731	832	+
Rw3G007190	Rw3G008200	102	Chr3g0457971	Chr3g0459561	122	+
Rw3G008220	Rw3G011750	354	Chr3g0460021	Chr3g0465661	431	+
Rw3G011750	Rw3G015900	416	Chr3g0465911	Chr3g0471601	470	+
Rw3G015980	Rw3G016940	97	Chr3g0471771	Chr3g0473291	119	-
Rw3G016970	Rw3G020450	349	Chr3g0473761	Chr3g0479761	434	+
Rw3G020750	Rw3G025450	471	Chr3g0479901	Chr3g0488151	598	+
Rw3G025400	Rw3G027410	202	Chr3g0488401	Chr3g0493691	273	+
Rw3G027420	Rw3G029600	219	Chr3g0494021	Chr3g0497471	256	+
Rw4G000380	Rw4G000820	45	Chr4g0384721	Chr4g0385541	57	-
Rw4G000910	Rw4G002530	163	Chr4g0386821	Chr4g0390351	229	+
Rw4G002450	Rw4G006350	391	Chr4g0390741	Chr4g0397311	481	+
Rw4G006450	Rw4G010000	356	Chr4g0397571	Chr4g0404181	460	+
Rw4G010030	Rw4G012600	258	Chr4g0404601	Chr4g0408971	317	+
Rw4G012630	Rw4G013720	110	Chr4g0409241	Chr4g0410821	125	+
Rw4G013720	Rw4G019810	610	Chr4g0411101	Chr4g0421021	730	+
Rw4G019820	Rw4G020430	62	Chr4g0421111	Chr4g0422041	73	-
Rw4G020460	Rw4G031440	1099	Chr4g0422091	Chr4g0437841	1257	+
Rw4G031450	Rw4G037750	631	Chr4g0439361	Chr4g0447321	681	+
Rw5G000040	Rw5G005300	527	Chr5g0000001	Chr5g0007361	600	+
Rw5G005540	Rw5G006180	65	Chr5g0007621	Chr5g0008561	75	-
Rw5G006190	Rw5G007570	139	Chr5g0008571	Chr5g0010391	150	+
Rw5G007580	Rw5G011230	366	Chr5g0011151	Chr5g0016881	444	+
Rw5G011080	Rw5G018290	722	Chr5g0017141	Chr5g0028591	873	+
Rw5G018330	Rw5G021640	332	Chr5g0028821	Chr5g0034321	412	+
Rw5G021650	Rw5G026480	484	Chr5g0034311	Chr5g0041861	580	+
Rw5G026520	Rw5G026820	31	Chr5g0042381	Chr5g0042791	35	+
Rw5G026850	Rw5G028480	164	Chr5g0043071	Chr5g0045901	204	+
Rw5G028730	Rw5G032030	331	Chr5g0046471	Chr5g0051211	375	+
Rw5G032140	Rw5G033170	104	Chr5g0051381	Chr5g0053031	125	-
Rw5G033170	Rw5G041520	836	Chr5g0053421	Chr5g0067511	1022	+
Rw5G041530	Rw5G046760	524	Chr5g0067751	Chr5g0076831	649	+
Rw5G046770	Rw5G047210	45	Chr5g0076851	Chr5g0077431	48	-
Rw5G047270	Rw5G048440	118	Chr5g0077451	Chr5g0079511	149	+
Rw5G048170	Rw5G048540	38	Chr5g0079791	Chr5g0080261	40	+
Rw5G048570	Rw5G049820	126	Chr5g0080541	Chr5g0083141	175	+
Rw5G049860	Rw5G050450	60	Chr5g0083151	Chr5g0084181	76	+
Rw6G000160	Rw6G001600	145	Chr6g0249281	Chr6g0251951	186	+
Rw6G001730	Rw6G003060	134	Chr6g0244001	Chr6g0246521	175	+
Rw6G002850	Rw6G004710	187	Chr6g0246791	Chr6g0249101	198	+
Rw6G004720	Rw6G007010	230	Chr6g0252481	Chr6g0256041	274	+
Rw6G007080	Rw6G007660	59	Chr6g0256161	Chr6g0256701	55	+
Rw6G007550	Rw6G008200	66	Chr6g0256761	Chr6g0257951	79	-
Rw6G008200	Rw6G009940	175	Chr6g0258041	Chr6g0261051	218	+
Rw6G009760	Rw6G014740	499	Chr6g0261901	Chr6g0270191	613	+
Rw6G014750	Rw6G036000	2126	Chr6g0270441	Chr6g0300721	2416	+
Rw6G036010	Rw6G045040	904	Chr6g0301221	Chr6g0312961	999	+
Rw7G000010	Rw7G010180	1018	Chr7g0176801	Chr7g0191221	1168	+
Rw7G010180	Rw7G016330	616	Chr7g0191591	Chr7g0200491	712	+
Rw7G016340	Rw7G019860	353	Chr7g0200711	Chr7g0206621	430	+
Rw7G019690	Rw7G020320	64	Chr7g0207261	Chr7g0208371	80	+
Rw7G020350	Rw7G024000	366	Chr7g0208641	Chr7g0214161	424	+
Rw7G024080	Rw7G028290	422	Chr7g0214511	Chr7g0220361	475	+

Rw7G028520	Rw7G034960	645	Chr7g0220381	Chr7g0231641	816	+
Rw7G034830	Rw7G038870	405	Chr7g0231921	Chr7g0238561	490	+
Rw7G039020	Rw7G039840	83	Chr7g0242491	Chr7g0243971	105	+
Rw7G039810	Rw7G042680	288	Chr7g0238641	Chr7g0242051	299	+
Total genes		30138			35778	
Annotated genes in genome		32674			45469	
Proportion		92.24%			78.69%	

Table S23. Synteny blocks between BT and Hibrand Saint-Oyant's haploOB tested via McScanX. "+" or "-" indicated the block matching direction.

BT_start	BT_end	Gene_num	NG_start	NG_end	Gene_num	direction
Rw1G000020	Rw1G000350	34	RC1G0001800	RC1G0006000	37	+
Rw1G000530	Rw1G005470	495	RC1G0010300	RC1G0093300	607	+
Rw1G005450	Rw1G006240	80	RC1G0095800	RC1G0106800	87	+
Rw1G006460	Rw1G007420	97	RC1G0107100	RC1G0122900	116	+
Rw1G007440	Rw1G009730	230	RC1G0126500	RC1G0148000	210	+
Rw1G009920	Rw1G010190	28	RC1G0148200	RC1G0153100	35	+
Rw1G010240	Rw1G010700	47	RC1G0152600	RC1G0157600	47	-
Rw1G010710	Rw1G010820	12	RC1G0170900	RC1G0172000	10	-
Rw1G010830	Rw1G011810	99	RC1G0157800	RC1G0176000	128	+
Rw1G011820	Rw1G013270	146	RC1G0184700	RC1G0205200	166	+
Rw1G013300	Rw1G021310	802	RC1G0204800	RC1G0319400	900	+
Rw1G021320	Rw1G022310	100	RC1G0323100	RC1G0336900	114	+
Rw1G022380	Rw1G023300	93	RC1G0337300	RC1G0350800	105	+
Rw1G023260	Rw1G031110	786	RC1G0353300	RC1G0460200	877	+
Rw1G031140	Rw1G034020	289	RC1G0463600	RC1G0504500	328	+
Rw1G034050	Rw1G034940	90	RC1G0504900	RC1G0516600	98	-
Rw1G034980	Rw1G039700	473	RC1G0516600	RC1G0575900	512	+
Rw1G039720	Rw1G041290	158	RC1G0581200	RC1G0599800	167	-
Rw1G041300	Rw1G041560	27	RC1G0577200	RC1G0581200	31	+
Rw2G000010	Rw2G003940	394	RC2G0000200	RC2G0047500	419	+
Rw2G003950	Rw2G004290	35	RC2G0047600	RC2G0051400	35	-
Rw2G004300	Rw2G004580	29	RC2G0051500	RC2G0055100	29	-
Rw2G004600	Rw2G004780	19	RC2G0055400	RC2G0057400	19	-
Rw2G004790	Rw2G005250	47	RC2G0058000	RC2G0063400	49	-
Rw2G005270	Rw2G005790	53	RC2G0063500	RC2G0069500	53	-
Rw2G005800	Rw2G006350	56	RC2G0069600	RC2G0075800	57	-
Rw2G006360	Rw2G007080	73	RC2G0076400	RC2G0084500	75	-
Rw2G007140	Rw2G009490	236	RC2G0096300	RC2G0128900	267	+
Rw2G009510	Rw2G016510	701	RC2G0131500	RC2G0223700	765	+
Rw2G016580	Rw2G021290	472	RC2G0229100	RC2G0287900	500	+
Rw2G020690	Rw2G021830	115	RC2G0349800	RC2G0365200	126	+
Rw2G021820	Rw2G022230	42	RC2G0368100	RC2G0374500	50	+
Rw2G022520	Rw2G026730	422	RC2G0288600	RC2G0349700	471	+
Rw2G026730	Rw2G027420	70	RC2G0374900	RC2G0382600	70	+
Rw2G027440	Rw2G028050	62	RC2G0382600	RC2G0392700	74	-
Rw2G028060	Rw2G033890	584	RC2G0390000	RC2G0479100	679	+
Rw2G035620	Rw2G036360	75	RC2G0086000	RC2G0095300	81	+
Rw2G037050	Rw2G037840	80	RC2G0479300	RC2G0489700	88	+
Rw2G038320	Rw2G039740	143	RC2G0489800	RC2G0505900	144	-
Rw2G040190	Rw2G042030	185	RC2G0506100	RC2G0532200	211	+
Rw2G044030	Rw2G049910	589	RC2G0533700	RC2G0618800	666	+
Rw2G049920	Rw2G051220	131	RC2G0622200	RC2G0639000	141	+
Rw2G051760	Rw2G052190	44	RC2G0639400	RC2G0647200	56	+
Rw2G052340	Rw2G055480	315	RC2G0650100	RC2G0693600	357	+
Rw3G000010	Rw3G008150	815	RC3G0315500	RC3G0420300	890	-
Rw3G008220	Rw3G009510	130	RC3G0298400	RC3G0312900	131	-
Rw3G009520	Rw3G011750	224	RC3G0267800	RC3G0296200	243	-
Rw3G011750	Rw3G013760	202	RC3G0241100	RC3G0265300	209	-
Rw3G013790	Rw3G015890	211	RC3G0203700	RC3G0228800	222	+
Rw3G016080	Rw3G016940	87	RC3G0230800	RC3G0241000	93	-
Rw3G016970	Rw3G020450	349	RC3G0143900	RC3G0197600	404	-
Rw3G020750	Rw3G023770	303	RC3G0095700	RC3G0142400	357	-
Rw3G023690	Rw3G025400	172	RC3G0069700	RC3G0095100	198	+
Rw3G025400	Rw3G025910	52	RC3G0060000	RC3G0068200	56	+
Rw3G025910	Rw3G027410	151	RC3G0028400	RC3G0054900	185	+
Rw3G027520	Rw3G028830	132	RC3G0008800	RC3G0028300	148	+
Rw3G028840	Rw3G029540	71	RC3G0000200	RC3G0007400	64	-
Rw4G000930	Rw4G002010	109	RC4G0004700	RC4G0029100	154	+
Rw4G002700	Rw4G008700	601	RC4G0029700	RC4G0119300	692	+
Rw4G008630	Rw4G008930	31	RC4G0139200	RC4G0144200	35	+

Rw4G009470	Rw4G009600	14	RC4G0121600	RC4G0123800	15	+
Rw4G009790	Rw4G010000	22	RC4G0120100	RC4G0121300	16	+
Rw4G010030	Rw4G011220	120	RC4G0124700	RC4G0138900	123	+
Rw4G011520	Rw4G012590	108	RC4G0144300	RC4G0161900	129	+
Rw4G012630	Rw4G019810	719	RC4G0164200	RC4G0268100	815	+
Rw4G019820	Rw4G020430	62	RC4G0268500	RC4G0275100	60	-
Rw4G020460	Rw4G031440	1099	RC4G0275600	RC4G0415400	1180	+
Rw4G031450	Rw4G037750	631	RC4G0428500	RC4G0501500	654	+
Rw5G000040	Rw5G005530	550	RC5G0000100	RC5G0070000	596	+
Rw5G005540	Rw5G006180	65	RC5G0070100	RC5G0079300	76	-
Rw5G006190	Rw5G007570	139	RC5G0079400	RC5G0096200	142	+
Rw5G007580	Rw5G011080	351	RC5G0103600	RC5G0157200	416	+
Rw5G010960	Rw5G014520	357	RC5G0159900	RC5G0206500	390	+
Rw5G015150	Rw5G026330	1119	RC5G0206900	RC5G0364500	1272	+
Rw5G026340	Rw5G026490	16	RC5G0379800	RC5G0381700	17	+
Rw5G026500	Rw5G026820	33	RC5G0386200	RC5G0390500	36	+
Rw5G026850	Rw5G027080	24	RC5G0394900	RC5G0397800	24	+
Rw5G026920	Rw5G028170	126	RC5G0363100	RC5G0379400	131	-
Rw5G028580	Rw5G030930	236	RC5G0398600	RC5G0434300	268	+
Rw5G031070	Rw5G031280	22	RC5G0456500	RC5G0458700	21	-
Rw5G031300	Rw5G032030	74	RC5G0459600	RC5G0468700	77	-
Rw5G032140	Rw5G033380	125	RC5G0435000	RC5G0455600	152	-
Rw5G033390	Rw5G034100	72	RC5G0469500	RC5G0480800	84	-
Rw5G034100	Rw5G034850	76	RC5G0481000	RC5G0492900	88	-
Rw5G034860	Rw5G035730	88	RC5G0493000	RC5G0504100	94	-
Rw5G035710	Rw5G035850	15	RC5G0504600	RC5G0507000	18	-
Rw5G036180	Rw5G041520	535	RC5G0507100	RC5G0580400	595	+
Rw5G041530	Rw5G046730	521	RC5G0582800	RC5G0657800	587	+
Rw5G046760	Rw5G047230	48	RC5G0658400	RC5G0667000	61	-
Rw5G047270	Rw5G048280	102	RC5G0667000	RC5G0695000	153	+
Rw5G048080	Rw5G048540	47	RC5G0697200	RC5G0706600	59	+
Rw5G048570	Rw5G049820	126	RC5G0712700	RC5G0732500	146	+
Rw5G049120	Rw5G049320	21	RC5G0713500	RC5G0717700	27	+
Rw5G049860	Rw5G050450	60	RC5G0733200	RC5G0741900	68	+
Rw6G000160	Rw6G004710	456	RC6G0000600	RC6G0067300	527	+
Rw6G004720	Rw6G005380	67	RC6G0068000	RC6G0077800	76	-
Rw6G005390	Rw6G007010	163	RC6G0078300	RC6G0100600	182	+
Rw6G007080	Rw6G009940	287	RC6G0106100	RC6G0148100	324	+
Rw6G009760	Rw6G011680	193	RC6G0157600	RC6G0181300	199	+
Rw6G011970	Rw6G025660	1370	RC6G0182000	RC6G0367700	1501	+
Rw6G025660	Rw6G031600	595	RC6G0370000	RC6G0442700	628	+
Rw6G031610	Rw6G033060	146	RC6G0487300	RC6G0503500	150	-
Rw6G033070	Rw6G034250	119	RC6G0455500	RC6G0469000	123	+
Rw6G034250	Rw6G035270	103	RC6G0443300	RC6G0455100	108	+
Rw6G035280	Rw6G035490	22	RC6G0476700	RC6G0479700	26	-
Rw6G035500	Rw6G036000	51	RC6G0481400	RC6G0487000	52	-
Rw6G036010	Rw6G036290	29	RC6G0469300	RC6G0472400	29	-
Rw6G036280	Rw6G044950	868	RC6G0503600	RC6G0607200	921	+
Rw6G037260	Rw6G037370	12	RC6G0521300	RC6G0522500	12	+
Rw7G000010	Rw7G010110	1011	RC7G0000200	RC7G0132600	1113	+
Rw7G010190	Rw7G028290	1811	RC7G0136400	RC7G0388000	2013	+
Rw7G028520	Rw7G034160	565	RC7G0388100	RC7G0472100	651	+
Rw7G034040	Rw7G034960	93	RC7G0474300	RC7G0485800	99	+
Rw7G034830	Rw7G036390	157	RC7G0488700	RC7G0508600	165	+
Rw7G036190	Rw7G038850	267	RC7G0511400	RC7G0542800	272	+
Rw7G039060	Rw7G040820	177	RC7G0543700	RC7G0565500	185	+
Rw7G041090	Rw7G042020	94	RC7G0565700	RC7G0572100	75	+
Rw7G042090	Rw7G042680	60	RC7G0574900	RC7G0580900	58	+
Total genes		29137			32217	
Annotated genes in genome		32674			39669	
Proportion		89.17%			81.21%	

Table S24. Sequence polymorphisms between BT and Raymond's haploOB genomes.

Variations	haploOB	BT
Assembly size	515,588,973	530,067,488
Total sequences	55	1,110
Aligned sequences	55 (100%)	1,108 (99.82%)
Total aligned bases	415,501,841(80.59%)	464,311,228(87.59%)
Total SNPs	7,308,896 (1.42%)	7,308,896 (1.38%)
Total high-quality SNPs	1,655,377 (0.32%)	1,655,377 (0.31%)
Total one-nt indels	5,155,992 (1.00%)	5,155,992 (0.97%)
Total high quality one-nt indels	100,366 (0.02%)	100,366 (0.02%)

Total indels (50-500nt)	1,989 (316,032)
Total indels (500-10000nt)	1,182 (3,052,440)
Tandem expansion/contraction (50-500nt)	172 (35,091)
Tandem expansion/contraction (500-10,000nt)	286 (965,516)
Repeat expansion/contraction (50-500nt)	1,071 (249,871)
Repeat expansion/contraction (500-10,000nt)	4,075 (15,647,901)
Total variants (indels larger than 50nt, tandem and repeat expansion/contraction)	8,875 (20,266,851)
Total variants (SNPs, indels, tandem and repeat expansion/contraction	32,731,739 (6.17% of BT or 6.35% of haploOB genomes)

Table S25. Summary of structural variation events between BT and Raymond's haploOB genomes. CPL, copy number loss in OB in reference to BT genome; CPG, copy number gain in OB; INV, inversion; TRANS, translocation.

Events	Count	Length (bp)
CPG (copy gain)	8,321	11,458,799
CPL (copy loss)	1,172	2,803,338
INV (inverted region)	410	29,436,927
TRANS (Translocated region)	7,131	22,135,633
total	17,034	65,834,697

Table S26. GO enrichment for genes located in structural variation regions between BT and Raymond's haploOB genomes. MF, molecular function; CC, cellular components; BP, biological process.

Variation Type	GO ID	Count	Enrichment Fold Change	FDR	GO type	GO function
CPL	GO:0004072	1	95.98125	0.002507935	MF	aspartate kinase activity
CPL	GO:0004412	1	63.9875	0.013312029	MF	homoserine dehydrogenase activity
CPL	GO:0004753	1	95.98125	0.002507935	MF	saccharopine dehydrogenase activity
CPL	GO:0005488	8	3.763970588	0.00492305	MF	binding
CPL	GO:0005769	1	47.990625	0.046034903	CC	early endosome
CPL	GO:0007568	2	29.53269231	6.3658E-05	BP	aging
CPL	GO:0008193	1	95.98125	0.002507935	MF	tRNA guanylyltransferase activity
CPL	GO:0009067	1	63.9875	0.013312029	BP	aspartate family amino acid biosynthetic process
CPL	GO:0009888	2	15.357	0.005405296	BP	tissue development
CPL	GO:0010638	2	21.32916667	0.000924461	BP	positive regulation of organelle organization
CPL	GO:0033177	1	95.98125	0.002507935	CC	proton-transporting two-sector ATPase complex, proton-transporting domain
CPL	GO:0042276	1	95.98125	0.002507935	BP	error-prone translation synthesis
CPL	GO:0042800	1	191.9625	0.00014549	MF	histone methyltransferase activity (H3-K4 specific)
CPL	GO:0051568	1	63.9875	0.013312029	BP	histone H3-K4 methylation
CPG	GO:0003676	37	1.852708426	0.015370227	MF	nucleic acid binding
CPG	GO:0003964	7	4.019706091	0.035155003	MF	RNA-directed DNA polymerase activity
CPG	GO:0004049	3	10.12104569	0.03181788	MF	anthranilate synthase activity
CPG	GO:0004066	5	11.24560633	5.90928E-05	MF	asparagine synthase (glutamine-hydrolyzing) activity
CPG	GO:0004565	13	5.236759961	2.45644E-06	MF	beta-galactosidase activity
CPG	GO:0006259	12	4.561598059	5.90928E-05	BP	DNA metabolic process
CPG	GO:0006278	7	4.107091006	0.03181788	BP	RNA-dependent DNA biosynthetic process
CPG	GO:0006529	3	16.19367311	0.002602023	BP	asparagine biosynthetic process
CPG	GO:0008171	11	4.36594128	0.000372797	MF	O-methyltransferase activity
CPG	GO:0008237	6	5.783454682	0.005209219	MF	metallopeptidase activity
CPG	GO:0010136	4	17.99297012	1.80176E-05	BP	ureide catabolic process
CPG	GO:0010333	7	5.106113143	0.004712714	MF	terpene synthase activity
CPG	GO:0015074	25	4.16503938	1.39096E-09	BP	DNA integration
CPG	GO:0015996	8	4.593949819	0.004712714	BP	chlorophyll catabolic process
CPG	GO:0016151	3	13.49472759	0.005931069	MF	nickel cation binding
CPG	GO:0016206	2	26.98945518	0.015370227	MF	catechol O-methyltransferase activity
CPG	GO:0016798	7	4.019706091	0.035155003	MF	hydrolase activity, acting on glycosyl bonds

CPG	GO:0030246	15	3.090395632	0.003013843	MF	carbohydrate binding
CPG	GO:0047652	4	17.99297012	1.80176E-05	MF	allantoate deiminase activity
INV	GO:0004022	4	11.41360089	0.025181581	MF	alcohol dehydrogenase (NAD+) activity
INV	GO:0004097	4	13.69632107	0.013173372	MF	catechol oxidase activity
INV	GO:0004503	3	17.12040134	0.030223387	MF	monophenol monooxygenase activity
INV	GO:0042409	4	9.783086479	0.030223387	MF	caffeoyl-CoA O-methyltransferase activity
INV	GO:0046148	4	9.783086479	0.030223387	BP	pigment biosynthetic process
TRANS	GO:0003964	11	5.546591805	2.3191E-05	MF	RNA-directed DNA polymerase activity
TRANS	GO:0006278	11	5.667169887	2.3191E-05	BP	RNA-dependent DNA biosynthetic process
TRANS	GO:0010136	3	11.84953704	0.037783444	BP	ureide catabolic process
TRANS	GO:0010275	3	11.84953704	0.037783444	BP	NAD(P)H dehydrogenase complex assembly
TRANS	GO:0015074	19	2.779521033	0.006589134	BP	DNA integration
TRANS	GO:0016772	13	3.020470225	0.037783444	MF	transferase activity, transferring phosphorus-containing groups
TRANS	GO:0047652	3	11.84953704	0.037783444	MF	allantoate deiminase activity

Table S27. GO enrichment information for BT- (374 genes with GO information) and haploOB- (801 genes with GO information) specific gene families. Type: BP, biological process; MF, molecular function; CC, cellular components. Group: CON, contraction; EXP, expansion.

	GO	P value	genes in specific	Genes in background	fdr	fold change	type	function
BT specific	GO:0055114	0.000763916	13	1304	0.046130466	0.41	BP	oxidation-reduction process
	GO:0003676	0.00068912	26	539	0.046130466	1.98	MF	nucleic acid binding
	GO:0005488	1.23E-06	26	408	0.000250753	2.62	MF	binding
	GO:0003964	0.000128496	6	47	0.011234255	5.24	MF	RNA-directed DNA polymerase activity
	GO:0006278	0.000101273	6	46	0.010329844	5.36	BP	RNA-dependent DNA biosynthetic process
	GO:0015074	7.69E-17	22	162	4.70E-14	5.58	BP	DNA integration
	GO:0048513	0.000829142	4	26	0.046130466	6.32	BP	organ development
	GO:0044446	6.14E-08	5	18	1.88E-05	11.41	CC	obsolete intracellular organelle part
	GO:0055028	9.35E-06	3	8	0.001430078	15.40	CC	cortical microtubule
	GO:0019277	0.000274725	2	4	0.021016493	20.53	BP	UDP-N-acetylgalactosamine biosynthetic process
	GO:0008760	5.01E-05	2	3	0.006137923	27.37	MF	UDP-N-acetylglucosamine 1-carboxyvinyltransferase activity
**haploOB specific**	GO:0016021	0.000332368	149	4625	0.048847348	0.77	CC	integral component of membrane
	GO:0015074	0.000230561	18	171	0.048590748	2.51	BP	DNA integration
	GO:0097159	0.000405613	11	83	0.048847348	3.16	MF	organic cyclic compound binding
	GO:1901363	0.000405613	11	83	0.048847348	3.16	MF	heterocyclic compound binding
	GO:0044444	0.000131823	8	44	0.037042248	4.34	CC	obsolete cytoplasmic part
	GO:0071704	6.52E-05	6	24	0.027477977	5.97	BP	organic substance metabolic process
	GO:0006259	3.22E-06	8	33	0.002714695	5.79	BP	DNA metabolic process

Table S28. GO enrichment information for BT expanded and contracted gene families. Type: BP, biological process; MF, molecular function; CC, cellular components. Group: CON, contraction; EXP, expansion.

GO	Nb.test	Test	Nb.background	Background	FDR	Type	Function	Fold Change	Group
GO:0006468	5	32	554	14803	0.020099477	BP	protein phosphorylation	4.18	CON
GO:0004674	5	32	409	14948	0.002898321	MF	protein serine/threonine kinase activity	5.71	CON
GO:0009505	2	35	102	15255	0.037757909	CC	plant-type cell wall	8.55	CON
GO:0000165	2	35	87	15270	0.020099477	BP	MAPK cascade	10.03	CON

GO:0016706	3	34	59	15298	7.81E-08	MF	2-oxoglutarate-dependent dioxygenase activity	22.88	CON
GO:0000038	1	36	14	15343	0.043645127	BP	very long-chain fatty acid metabolic process	30.44	CON
GO:0009556	1	36	13	15344	0.035463878	BP	microsporogenesis	32.79	CON
GO:0000159	1	36	12	15345	0.026750673	CC	protein phosphatase type 2A complex	35.52	CON
GO:0009805	1	36	12	15345	0.026750673	BP	coumarin biosynthetic process	35.52	CON
GO:0009963	1	36	11	15346	0.020099477	BP	positive regulation of flavonoid biosynthetic process	38.75	CON
GO:0010025	1	36	11	15346	0.020099477	BP	wax biosynthetic process	38.75	CON
GO:0010252	1	36	10	15347	0.015551659	BP	auxin homeostasis	42.63	CON
GO:0052543	1	36	10	15347	0.015551659	BP	callose deposition in cell wall	42.63	CON
GO:0072488	1	36	9	15348	0.010299026	BP	ammonium transmembrane transport	47.37	CON
GO:0000148	1	36	8	15349	0.005821179	CC	1,3-beta-D-glucan synthase complex	53.30	CON
GO:0003843	1	36	8	15349	0.005821179	MF	1,3-beta-D-glucan synthase activity	53.30	CON
GO:0006075	1	36	8	15349	0.005821179	BP	(1->3)-beta-D-glucan biosynthetic process	53.30	CON
GO:0009922	1	36	8	15349	0.005821179	MF	fatty acid elongase activity	53.30	CON
GO:0008519	1	36	7	15350	0.003494407	MF	ammonium transmembrane transporter activity	60.91	CON
GO:0047672	1	36	7	15350	0.003494407	MF	anthranilate N-benzoyltransferase activity	60.91	CON
GO:0030497	1	36	4	15353	7.30E-05	BP	fatty acid elongation	106.62	CON
GO:0006559	1	36	3	15354	5.38E-06	BP	L-phenylalanine catabolic process	142.17	CON
GO:0034613	1	36	3	15354	5.38E-06	BP	cellular protein localization	142.17	CON
GO:0009044	1	36	2	15355	7.81E-08	MF	xylan 1,4-beta-xylosidase activity	213.26	CON
GO:0009800	1	36	2	15355	7.81E-08	BP	cinnamic acid biosynthetic process	213.26	CON
GO:0016206	1	36	2	15355	7.81E-08	MF	catechol O-methyltransferase activity	213.26	CON
GO:0045548	1	36	2	15355	7.81E-08	MF	phenylalanine ammonia-lyase activity	213.26	CON
GO:0047172	1	36	2	15355	7.81E-08	MF	shikimate O-hydroxycinnamoyltransferase activity	213.26	CON
GO:0047205	1	36	2	15355	7.81E-08	MF	quinate O-hydroxycinnamoyltransferase activity	213.26	CON
GO:0003676	26	297	539	14818	0.003799638	MF	nucleic acid binding	2.41	EXP
GO:0005488	22	301	408	14949	0.002357545	MF	binding	2.68	EXP
GO:0044238	10	313	140	15217	0.02241489	BP	primary metabolic process	3.47	EXP
GO:0015074	20	303	162	15195	7.16E-14	BP	DNA integration	6.19	EXP
GO:0043170	6	317	28	15329	1.73E-06	BP	macromolecule metabolic process	10.36	EXP

Table S29. Genomic positions for potential QTL regions (see Fig. 3 and S20).

QTL region	Chrom ID	BT Start	BT End	BT length (bp)	OB Start (Raymond et al. 2018)	OB end	OB length (bp)
**QTL1**	3	13,967,186	16,231,712	2,264,526	14,980,474	17,533,647	2,553,174
**QTL2**	7	19,033,610	20,276,089	1,242,479	21,232,966	22,338,966	1,106,001
**QTL3**	7	980,078	2,637,631	1,657,554	1,082,938	2,731,368	1,648,431

Table S30. Colocalization of QTL1 with known QTL regions related to important traits on Chr3 reported by Hibrand Saint-Oyant et al. (2018).

Traits	Chr3 Position in haploOB (Mb)	BT location *
Continuous flowering	28 ~ 33 Mb	16.9 ~ 18.5 Mb

Double flower	33 ~ 33.2 Mb	13.2 ~ 13.4 Mb
Self-incompatibility	40.5 ~ 44.5 Mb	2.2 ~ 6.6 Mb
Prickle density	**34.5 ~ 38 Mb**; 38.5 ~ 41 Mb; 42 ~ 45 Mb	**13.97 ~ 16.23 Mb #**

Notes:
1) Comparisons were based on synteny analysis of genes.
2) * The BT homologous positions for the QTL regions of continuous flowering, double flower and self-incompatibility were identified with BLAST using corresponding marker sequences from haploOB genome.
3) # Position for QTL1 in BT genome identified in this study, overlapped with the first segment in haploOB genome (in bold).

Table S31. Genes in QTL regions were under a strong purifying selection (non-parametric *Wilcoxon rank sum test*).

parameter	Region 1	Region 2	P value	Significant level
Ka/Ks	QTL1	QTL2	0.938	ns.
	QTL1	QTL3	0.565	ns.
	QTL2	QTL3	0.651	ns.
	QTL1	Rest of genome	0.051	*
	QTL2	Rest of genome	0.141	ns.
	QTL3	Rest of genome	0.120	ns.
Ks	QTL1	QTL2	0.00016	***
	QTL1	QTL3	0.17179	ns.
	QTL2	QTL3	0.00522	**
	QTL1	Rest of genome	2.5e-06	****
	QTL2	Rest of genome	0.34272	ns.
	QTL3	Rest of genome	0.00017	***

Table S32. Statistical analysis of the relative water contents in different tissues at different developmental stages in one prickly rose genotype (C220). Analysis was carried out with pairwise *Wilcoxon test* in *R*. ***, *p*<0.001; **, *p*<0.01; *ns*, not significant.

Dev.Stage	Tissue 1	Tissue 2	P value	P.adj	Significance level
I	leaf	prickle	7.6e-06	0.000	****
I	leaf	epidermis	7.6e-06	0.000	****
I	leaf	stele	7.6e-06	0.000	****
I	prickle	epidermis	1.5e-05	0.000	****
I	prickle	stele	2.3e-05	0.000	****
I	epidermis	stele	1.5e-05	0.000	****
II	leaf	prickle	7.6e-06	0.000	****
II	leaf	epidermis	7.6e-06	0.000	****
II	leaf	stele	7.6e-06	0.000	****
II	prickle	epidermis	7.6e-06	0.000	****
II	prickle	stele	0.00193	0.008	**
II	epidermis	stele	1.5e-05	0.000	****
III	leaf	prickle	7.6e-06	0.000	****
III	leaf	epidermis	7.6e-06	0.000	****
III	leaf	stele	1.5e-05	0.000	****
III	prickle	epidermis	7.6e-06	0.000	****
III	prickle	stele	0.36922	0.740	ns
III	epidermis	stele	0.00033	0.002	***
IV	leaf	prickle	7.6e-06	0.000	****
IV	leaf	epidermis	7.6e-06	0.000	****
IV	leaf	stele	0.00129	0.006	**
IV	prickle	epidermis	0.00011	0.001	***
IV	prickle	stele	0.79871	0.800	ns
IV	epidermis	stele	0.19639	0.590	ns
old	leaf	prickle	1.9e-06	0.000	****
old	leaf	epidermis	0.0032	0.003	**

old	leaf	stele	1.9e-06	0.000	****
old	prickle	epidermis	1.9e-06	0.000	****
old	prickle	stele	3.8e-06	0.000	****
old	epidermis	stele	1.9e-06	0.000	****

Table S33. Measurement of the relative water contents (RLC) in randomly selected 39 rose genotypes.

Line ID	genotype name	type	section number	prickle number	Prickle RLC	Epidermis RLC	Stele RLC
Rs021	*R. taiwanensis* Nakai.	wild rose	1	15	0.8495	0.7820	0.8990
			2	15	0.8240	0.7809	0.8935
			3	15	0.7555	0.7445	0.7196
Rs023	*R. palustris*	wild rose	1	6	0.8484	0.7425	0.8588
			2	10	0.7223	0.6971	0.7552
			3	11	0.6446	0.6702	0.6226
Rs024	*R. multiflora* Thunb. var. Carnea Thory	wild rose	1	9	0.8300	0.7638	0.8997
			2	9	0.8235	0.7525	0.8822
			3	9	0.7528	0.7214	0.7640
Rs026	*R. damascena*	wild rose	1	12	0.8590	0.7918	0.8979
			2	15	0.7916	0.7182	0.8573
			3	16	0.7745	0.7023	0.7067
Rs027	*R. banksiae* 'albo-plena'	wild rose	1		0.8009	0.7575	0.8874
			2	11	0.7620	0.6790	0.8223
			3	14	0.7421	0.6645	0.6931
Rs028	*R. longicuspis* Bertal	wild rose	1	15	0.7103	0.6866	0.8091
			2	13	0.4925	0.5930	0.6026
			3		0.4758	0.6070	0.5769
Rs033	*R. sp*	wild rose	1	12	0.7649	0.7048	0.8395
			2	12	0.7149	0.6861	0.7252
Rs034	*R. laxa* Retzius	wild rose	1	8	0.8489	0.7794	0.8872
			2	9	0.8380	0.7594	0.8550
			3	9	0.8027	0.7249	0.7320
Rs035	*R. cymosa* Trattinnick	wild rose	1	15	0.7764	0.7621	0.8913
			2	11	0.7789	0.7412	0.8726
			3	10	0.8269	0.7078	0.8637
Rs036	*R. sp*	wild rose	1	15	0.8262	0.7452	0.8779
			2	15	0.7690	0.6997	0.7024
			3	19	0.7676	0.6858	0.6581
Rs037	*R. sp*	wild rose	1	15	0.7931	0.7797	0.8934
			2	17	0.7651	0.7126	0.7913
			3	22	0.7345	0.6860	0.6544
Rs038	*R. sp*	wild rose	1	19	0.8247	0.7565	0.8829
			2	14	0.7965	0.7222	0.7599
			3	15	0.7833	0.7046	0.6400
Rs039	*R. sp*	wild rose	1	18	0.7658	0.7325	0.8663
			2	13	0.6822	0.6739	0.7620
Rs010	*R. odorata* 'Glandular Sepal'	China rose	1	9	0.8567	0.7897	0.9010

			2	8	0.8347	0.7520	0.7873
Rs032	*R. chinensis* 'Spontenea'	China rose	1	4	0.8182	0.7749	0.8873
			2	5	0.6325	0.6440	0.6343
Rs029	*R. odorata* (Andr.) Sweet var. erubescens (Focke) Yu et Ku	China rose	1	9	0.8614	0.7783	0.8942
			2	9	0.8912	0.7840	0.9043
			3	12	0.8625	0.7454	0.7792
Rs015	*R. chinensis* 'Tiebahong'	China rose	1	4	0.8652	0.7865	0.9087
			2	5	0.8471	0.7726	0.8928
			3	6	0.8060	0.7342	0.8020
Rs012	*R. odorata* 'Pink Blush'	China rose	1	5	0.8707	0.8017	0.9077
			2	7	0.7862	0.7473	0.7997
Rs042	*R. chinensis* 'Old Blush'	China rose	1	21	0.7099	0.5309	0.5675
Rs001	*R.* 'Oklahoma'	Modern rose	1	16	0.8288	0.7633	0.8646
			2	36	0.8744	0.8163	0.9074
			3	36	0.8809	0.7967	0.9075
Rs002	*R.* 'Republique de Montmartre'	Modern rose	1	5	0.8630	0.7872	0.8916
			2	6	0.8260	0.7384	0.7613
			3	8	0.7496	0.7140	0.6349
Rs003	*R.* 'Red Hat'	Modern rose	1	9	0.8818	0.8256	0.9201
			2	15	0.8630	0.7799	0.8844
			3	15	0.8078	0.7541	0.6958
Rs004	*R.* 'Crocus Rose'	Modern rose	1	6	0.8237	0.7574	0.8268
			2	9	0.8092	0.7451	0.7069
			3	9	0.7834	0.7277	0.6479
Rs005	*R.* 'Leonardo da Vinci'	Modern rose	1	9	0.8700	0.8149	0.9143
			2	8	0.8751	0.7878	0.8998
			3	12	0.8433	0.7575	0.7865
Rs006	*R.* 'Angela'	Modern rose	1	8	0.8398	0.7769	0.8989
			2	8	0.8106	0.7511	0.8800
			3	8	0.7243	0.7150	0.6519
Rs007	*R.* 'Sweet Pretty'	Modern rose	1	4	0.8180	0.7423	0.8397
			2	4	0.8100	0.7295	0.7380
			3	6	0.7818	0.7139	0.6937
Rs008	*R.* 'Angel Smile'	Modern rose	1	6	0.8448	0.7557	0.8546
			2	8	0.8247	0.7425	0.7176
			3	11	0.7899	0.7333	0.7377
Rs009	*R.* 'Carmen'	Modern rose	1	9	0.7574	0.7130	0.8305
			2	9	0.6622	0.6825	0.6667
			3	8	0.6576	0.6724	0.6201
Rs011	*R.* 'Princesse de Monaco'	Modern rose	1	8	0.8618	0.7790	0.8976
			2	14	0.8531	0.7664	0.8458
			3	23	0.8288	0.7721	0.7308
Rs013	*R.* 'Claire Austin'	Modern rose	1	4	0.7361	0.6954	0.6815
			2	5	0.7085	0.6776	0.6350
Rs014	*R.* 'Aschermittwoch'	Modern rose	1	7	0.7974	0.7400	0.8693
			2	10	0.6165	0.6912	0.7025
Rs016	*R.* 'Jasmina'	Modern rose	1	10	0.8413	0.8167	0.9031

			2	12	0.8475	0.8006	0.9026
			3	15	0.8021	0.7800	0.8442
Rs017	*R.* 'Tchaikovski'	Modern rose	1	5	0.8315	0.7539	0.8549
			2	9	0.7964	0.7577	0.7329
			3	10	0.7862	0.7605	0.7003
Rs018	*R.* 'Caramella'	Modern rose	1	8	0.8780	0.7827	0.9041
			2	6	0.8734	0.7854	0.8922
			3	6	0.8525	0.7531	0.8666
Rs019	*R.* 'Durance'	Modern rose	1	10	0.8718	0.8145	0.9463
			2		0.8280	0.7731	0.8808
			3	31	0.7572	0.7620	0.6774
Rs020	*R.* 'William Morris'	Modern rose	1	6	0.8449	0.8037	0.9019
			2	6	0.8468	0.7711	0.8620
			3	9	0.7498	0.7509	0.6616
Rs022	*R.* 'Gartendirektor Otto Linne'	Modern rose	1	6	0.8430	0.7871	0.8882
			2	3	0.8667	0.8069	0.9067
			3	4	0.8210	0.7581	0.8550
Rs025	*R.* 'Yee Chino'	Modern rose	1	6	0.7554	0.7273	0.8633
			2	5	0.7116	0.6940	0.7192
			3		0.5501	0.6590	0.5234
Rs030	*R.* 'Altissimo'	Modern rose	1	10	0.8118	0.7516	0.8702
			2	15	0.7919	0.7349	0.7160
			3	30	0.7339	0.7304	0.6606
Rs031	*R.* 'Red Mozart'	Modern rose	1	6	0.8068	0.7395	0.8605
			2		0.7114	0.7005	0.7146

Table S34. Phenotype and data resources for genotypes used in genetic divergence analysis.

Genotype	chloroplast genome ID	genome length (bp)	Prickle phenotype	references
R. arvensis		156661	prickly	this study
R. banksiae	NC042194	156575	NA	Wang et al. (2019)
R. banksiae f. *lutea*		156544	prickle-free	this study
R. chinensis 'Old Blush'	CM009590	156546	NA	Raymond et al. (2018)
R. chinensis f. *mutabilis*		156575	prickly	this study
R. chinensis var. *spontanea*		156603	prickly	this study
R. cv. Emira		156580	prickle-free	this study
R. cv. Star 'n' Strips		156504	prickle-free	this study
R. damascena		156575	prickly	this study
R. lichiangensis	KY419934	130564	NA	Zhang et al. (2017)
R. lucieae	NC040997	156506	NA	Jeon and Kim (2019)
R. majalis		157258	prickly	this study
R. maximowicziana	NC040960	156405	NA	Jeon and Kim (2019)
R. moschata		156575	prickly	this study
R. multiflora	NC039989	156592	NA	Si unpublished
R. odorata var. *gigantea*		156654	prickly	this study
R. persica	KY419918	130948	NA	Zhang et al. (2017)
R. praelucens	NC037492	157186	NA	Jian et al. (2018).
R. roxburghii	NC032038	156749	NA	Wang et al. (2018)
R. roxburghii f. *normalis*	KY419960	130607	NA	Zhang et al. (2017)
R. rugosa		157027	prickly	this study
R. wichuraiana 'Basye's Thornless'	MN689790	156500	prickle-free	Cui et al. (2020)
Rubus crataegifolius	NC039704	155714	NA	Yang et al. (2017)
Potentilla freyniana	NC041210	156381	NA	Park et al. (2019)

Table S35. List of candidate genes related to MYB, bHLH, and WRKY families in the QTL segments.

Region	Chrom ID	BT ID	OB ID	Family	Notes
QTL1	3	*Rw3G015040*	*Chr3g0470381*	MYB	*MYB38-like*
		Rw3G015190	*Chr3g0470621*	MYB	*MYB27-like*
QTL2	7	*Rw7G018230*	*Chr7g0203911*	MYB	*TT2-like*
		Rw7G018330	*Chr7g0204081*	MYB	*MYBH/KUA1-like*
QTL3	7	*Rw7G001440*	*Chr7g0178681*	MYB	*MYB308-like*
		Rw7G001450	*Chr7g0178781*	MYB	*MYBD-like*
		Rw7G001530	*Chr7g0178691*	MYB	*MYB4R1-like*
		Rw7G002410	*Chr7g0179981*	MYB	*MYBD-like*
		Rw7G002620	*Chr7g0180151*	MYB	*MYB35-like*
		Rw7G003060	*Chr7g0180741*	WRKY	*WRKY61-like*
		Rw7G002590	*Chr7g0180121*	bHLH	*bHLH79/BPE-like*
		Rw7G003170	*Chr7g0181001*	bHLH	*bHLH35/KDR-like*
		Rw7G003210	*Chr7g0180941*	bHLH	*bHLH161-like*

Table S36. Primers used for validation of candidate genes using RT-qPCR method.

Primer ID	Direction	Gene (Rc/Rw)	Sequence (5`to 3`)
R101	F	*UBC* (housekeeping) *Chr7g0195591/Rw7G013020*	GCCAGAGATTGCCCATATGTA
R102	R		TCACAGAGTCCTAGCAGCACA
R271	F	*Chr3g0468221/Rw3G013440*	CCTCAAACCCAGGAGCATC
R272	R		CAACAGCTTGATCCCTGAGAG
R317	F	*Chr1g0382931/Rw1G040930*	AAGCTAGGGTTTTCGCCTCC
R318	R		TCGTGGAATGCTCCTTGTCC
R337	F	*Chr1g0380141/Rw1G038470*	CGGGTCGAACAGCAGTAGAAT
R338	R		CACTGAAAGTTCGACGCGGA
R363	F	*Chr7g0193591/Rw7G011700*	TCCTCGGAAACAGGTGGTCT
R364	R		AGTTGTTGACCACACCGAGC
R365	F	*Chr7g0180121/Rw7G002590*	TGAGCCAGCCAAGCAAGATT
R366	R		ACCTTATTACATCCGGGAACCA
R367	F	*Chr3g0470621/Rw3G015190*	TCGGAGGTGGGATGCATTAG
R368	R		TAGTTCAACCACCGCAACCT
R369	F	*Chr3g0470611/Rw3G015180*	GGTCAAGTCACACCCGTCAA
R370	R		TAAGCTCACGTCCGATGGGT
R371	F	*Chr7g0180851/Rw7G003170*	CAAGGTTTCAGCATCGACGG
R372	R		GTCGCTCGCTTAGGTCATCC
R373	F	*Chr7g0180941/Rw7G003210*	TCTCAACCCACAGAGGACGA
R374	R		TGCTGATGCCTTTGTGTTTGG
R375	F	*Chr7g0180121/Rw7G002590*	GCTGTGAGAAGAAGACGACCA
R376	R		TTCCACAACGAAGCAACCCT
R377	F	*Chr7g0203911/Rw7G018230*	TGTTCCAGCAGACAAAGACGA
R378	R		TTGTTCACCCACTCCAACCCA
R381	F	*Chr2g0138951/Rw2G032830*	GCCTGTCTCTCCTTATGCCC
R382	R		GGTTTGCAATTGTTTGCGCC
R383	F	*Chr3g0470611/Rw3G015180*	CCACTCCCCTATTCTGCGTG
R384	R		GACCCGGAACAATGCAATCG
R385	F	*Chr3g0479101/Rw3G019920*	ATCCTTCTCTCAGCTGCCCT
R386	R		GATTCCGACTCGTGGTTGGT
